# New Application of *cyclo*Saligenyl Prodrugs Approach for the Delivery of Fosfoxacin Derivatives in Mycobacteria

**DOI:** 10.3390/molecules28237713

**Published:** 2023-11-22

**Authors:** Mathilde Munier, Denis Tritsch, Didier Lièvremont, Michel Rohmer, Catherine Grosdemange-Billiard

**Affiliations:** Laboratoire Chimie et Biochimie de Molécules Bioactives, Université de Strasbourg/CNRS, UMR 7177, Institut Le Bel, 4 Rue Blaise Pascal, 67081 Strasbourg, France

**Keywords:** isoprenoid biosynthesis, *cyclo*Saligenyl prodrug, fosfoxacin, deoxyxylulose 5-phosphate reducto-isomerase, *Mycobacterium smegmatis*

## Abstract

In this work, we implemented for the first time the *cyclo*Saligenyl prodrug strategy to increase the bioavailability of fosmidomycin phosphate analogs in bacteria. Here, we report the synthesis of 34 *cyclo*Saligenyl prodrugs of fosfoxacin and its derivatives. Among them, fifteen double prodrugs efficiently prevented the growth of the non-pathogenic, fast-growing *Mycobacterium smegmatis*.

## 1. Introduction

The overuse and misuse of antibiotics, coupled with the absence of new drug development, resulted in a rapid and alarming emergence of microbial resistance. To date, this silent pandemic is an increasingly serious threat to global public health, as recently reported by the World Health Organization, a threat exacerbated by the absence of new drug development [1]. The multidrug- and extensively drug-resistant strains of *Mycobacterium tuberculosis* are prime examples. As these strains are resistant to at least the first line of antitubercular drugs, isoniazid and rifampicin, which received their marketing authorizations in 1952 and 1972, respectively, they are responsible for tuberculosis, which is one of the top ten causes of death worldwide [2,3]. Therefore, there is an urgent need to develop new antitubercular drugs that act differently from the antibiotics currently on the market. In addition, because mycobacteria have a thick lipophilic cell wall, they are naturally resistant to known antibiotics, making the development of new antitubercular drugs a challenge [4].

**Scheme 1:**
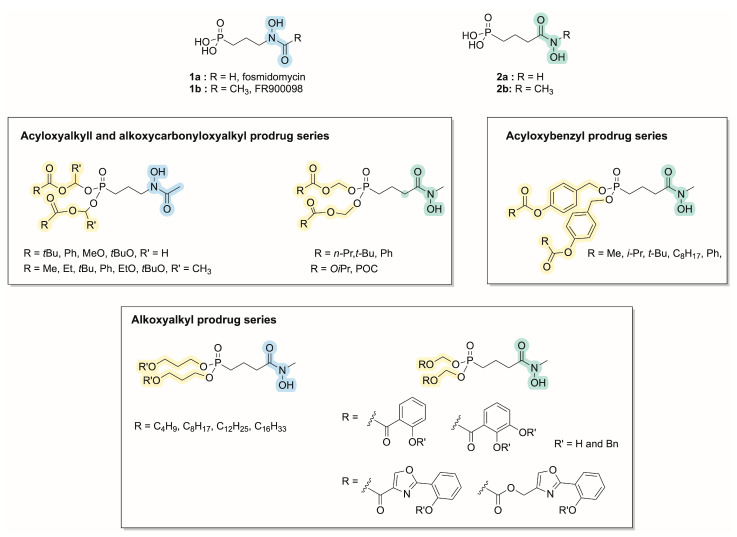
Natural and synthetic phosphonate DXR inhibitors **1**–**2** and examples of their prodrugs.

A potential target is the 1-deoxy-D-xylulose 5-phosphate reductoisomerase (DXR), the second enzyme of the 2-*C*-methyl-D-erythrirol 4-phosphate (MEP) pathway, the only source of isopentenyl diphosphate (IPP) and dimethylallyl diphosphate (DMAPP) in most pathogenic bacteria. As the MEP pathway is absent in humans, it represents a very attractive target for the development of novel antimicrobials [5]. Fosmidomycin **1a** and its close relative FR-900098 **1b** (Scheme 1), two natural phosphonate compounds [6], were found to have a strong antibacterial activity) acting as potent inhibitors of DXR [7].

**Scheme 2:**
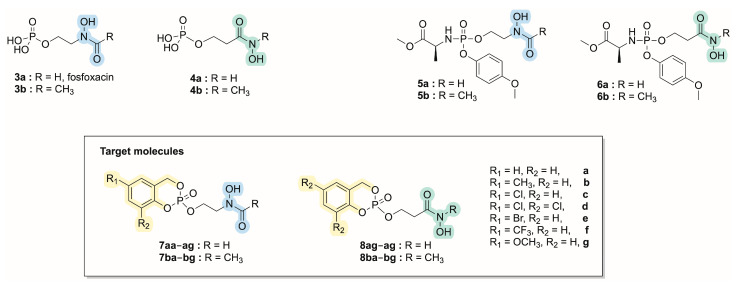
Natural and synthetic phosphate DXR inhibitors **3**–**4**, arylphosphoramidate **5**–**6** and *cyclo*Saligenyl prodrug analogues **7**–**8**.

Due to its pharmacokinetic properties, moderate oral bioavailability and fast clearance, fosmidomycin cannot, however, be used as a drug [8]. To circumvent these issues, much research has been devoted to improving the efficiency of fosmidomycin derivatives. Within this framework, we synthesized two hydroxamate phosphonic acids **2a** and **2b** [9] and the phosphate analog of fosmidomycin, fosfoxacin **3a**, and the related analogs **3b** and **4a**–**4b** [10] (Scheme 2). Even if the IC_50_ values of the *N*-methylated derivatives **2b** and **4b** against *Escherichia coli* and *Mycobacterium smegmatis* DXRs are similar to those of fosmidomycin **1a**, none of those natural and synthetic phosphonates **1**–**3** and phosphates **2**–**4** inhibits the growth of *M. smegmatis* cells [10].

In fact, in contrast to *E. coli*, the mycobacteria lack the transporters allowing the uptake of fosmidomycin derivatives (GlpT and UhpT, the glycerol 3 phosphate and glucose 6 phosphate transporters, respectively) [11]. Besides, these bacteria have a complex, highly lipophilic cell wall, limiting the uptake of diverse chemical compounds as potential antibiotics, rendering the fight against these pathogenic bacteria very difficult. To circumvent the lack of uptake and to increase the bioavailability of phosphonate and phosphate hydrophobic antibiotics, most of the research groups, including ours, focused on prodrug strategies.

The concept of prodrug was introduced in the late 1950s by A Albert and is defined as an inactive pharmaceutical derivative that can be in vivo biotransformed, enzymatically, or chemically into the active parent drug [12].

This strategy is often used to optimize the delivery and the cellular uptake of drugs containing a phosphate or phosphonate group. In fact, such functional groups are negatively charged at physiological pH, a feature restricting their cellular uptake by passive diffusion. Moreover, phosphorylated compounds are rarely developed as potential antibiotics due to their sensitivity towards the hydrolytic activity of the phosphatases. Therefore, the prodrug approach has been adopted to bypass this lack of uptake and to increase the bioavailability of these hydrophilic compounds by (i) masking the charged phosphonate moiety with acyloxymethyl esters, alkoxycarbonyloxymethyl esters, acyloxybenzyl or alkoxyalkyl esters [13,14] (Scheme 1) (ii) replacing the charged phosphate with an aryl phosphoramidate moiety developed by McGuigan to deliver monophosphorylated nucleoside analogs inside the cell (Scheme 2). This method, also called the “ProTide approach” has been largely applied to improve the pharmacological properties of antiviral and anticancer agents [15,16].

The cleavage of these prodrugs is mediated by key endogenous enzymes (esterase, phospholipase, phosphoramidase), which are present in the target microorganism and able to release the parent drug. We have recently reported that the “ProTide” approach may not be suitable for the delivery of fosfoxacin analogs, probably due to the reactivity of the prodrugs and the resulting fast abiotic or enzymatic hydrolysis [17]. Instead of using enzymatic cleavage to release the drugs into the cell from the classically used phosphate masking groups, we investigated the opportunity of using the *cyclo*Saligenyl prodrug of fosfoxacin and its derivatives to inhibit the growth of *M. smegmatis.* The *cyclo*Sal approach, developed by C. Meier for the synthesis of nucleotide prodrugs, improved antiviral activity through the successful intracellular delivery of lipophilic nucleotide derivatives [18,19]. In contrast to the classically used phosphate masking groups, which are cleaved by enzymes, the intracellular cleavage of *cyclo*Saligenyl prodrugs is based on a completely pH-driven, chemically efficient, and highly selective hydrolysis mechanism. Moreover, such a masking should also protect the inhibitors against hydrolysis by phosphatases. To our knowledge, the *cyclo*Sal prodrug approach has never been used to deliver the parent phosphate drug into bacteria.

**Scheme 3:**
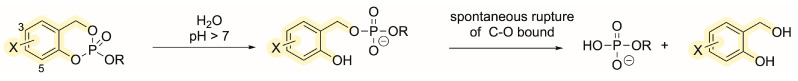
Hydrolysis mechanism of *cyclo*Sal group.

In this work, we synthesized a series of *cyclo*Saligenyl prodrugs of fosfoxacin and its analogs **7** and **8** and tested their growth inhibitory power on *E. coli* and the non-pathogenic, fast-growing *M. smegmatis*. The phosphate parent compounds are expected to be released from the prodrugs at an intracellular pH greater than 7 via a cascade reaction: selective cleavage of the most labile phenyl phosphate ester bond, followed by spontaneous rupture of the benzyl phosphate ester bond releasing the active drug and salicyl alcohol [20] (Scheme 3).

As the kinetic hydrolysis being is modulated by the substituent on the aromatic ring at C-3 or C-5 [21], we investigated the influence of electron-withdrawing (Cl, Br, CF_3_) and electron-donating (CH_3_, OCH_3_) substituents on bacterial growth inhibition tests of these *cyclo*Saligenyl prodrugs.

## 2. Results and Discussion

### 2.1. Chemistry

**Scheme 4:**
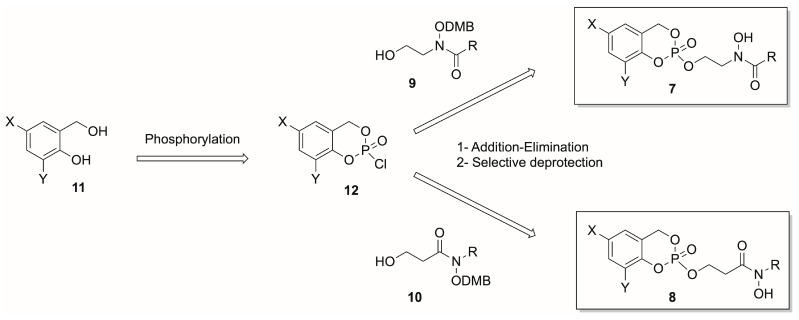
Synthetic approach of *cyclo*Saligenyl prodrugs of fosfoxacin derivatives **7** and **8**.

**Scheme 5:**
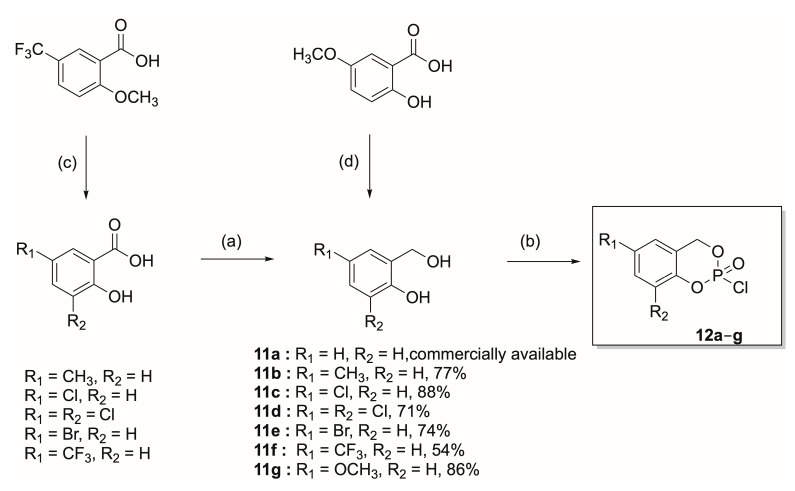
Synthesis of *Cyclo*Salphosphochloridates **12a**–**g**. Reagents and conditions: (**a**) LiAlH_4_, 0 °C to RT; (**b**) P(O)Cl_3_, TEA, THF, −78 °C to RT; (**c**) iodocyclohexane, DMF, reflux; (**d**) NaBH_4_, BF_3_.Et_2_O, THF, reflux; (**d**) NaBH_4_, THF, 0 °C to RT.

**Scheme 6:**
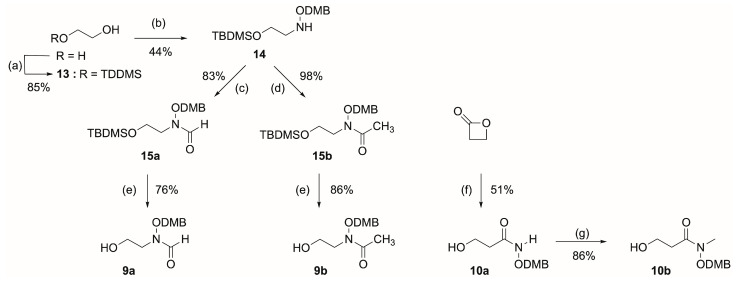
Synthesis of *O*-protected retrohydroxamic and hydroxamic acids **9** and **10.** Reagents and conditions: (**a**) NaH, TBDMSCl, THF, 0 °C to RT; (**b**) (i) Tf_2_O, 2,6-dimethylpyridine, CH_2_Cl_2_, −78 °C; (ii) H_2_NODMB, CH_2_Cl_2_, −78 °C to RT; (**c**) HCOOH/Ac_2_O (5:1), THF, RT; (**d**) Ac_2_O, pyridine, RT; (**e**) TBAF.3(H_2_O), THF, RT; (**f**) (i) H_2_NODMB, LiHMDS, THF, −78 °C; (ii) TBAF.3(H_2_O), THF, RT; (**g**) MeI, K_2_CO_3_, acetone, reflux.

**Scheme 7:**
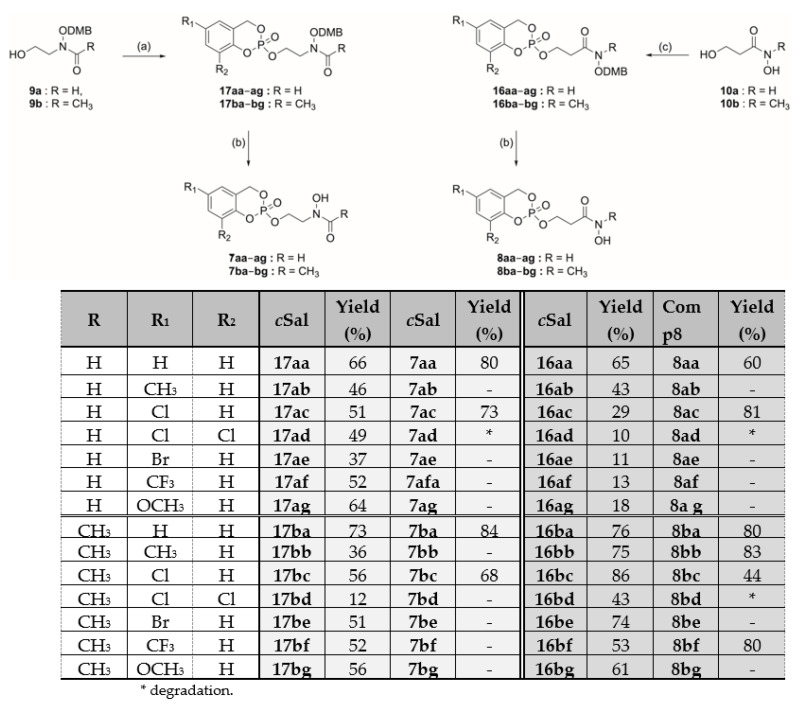
Synthesis of retrohydroxamic and hydroxamic *cyclo*Saligenyl prodrugs **7** and **8**. Reagents and reaction conditions: (**a**) TEA, DMAP, CH_2_Cl_2_, −40 °C to RT; (**b**) 2% TFA, CH_2_Cl_2_, RT. (**c**) R = H, pyridine, CH_2_Cl_2_, −40 °C to RT; R = CH_3_, TEA, DMAP, CH_2_Cl_2_, −40 °C to RT.

The strategy to synthesize the *cyclo*Sal prodrugs **7** and **8** is outlined in Scheme 4. The key step is the addition–elimination of *O*-protected retrohydroxamic and hydroxamic acids **9** and **10** on the *cyclo*Salphosphochloridate derivatives **12**, obtained by phosphorylation of the differently substituted salicyl alcohols **11** using phosphorus (V) chemistry (Scheme 4). The choice of the hydroxylamine protecting group is crucial in the synthesis of *cyclo*Saligenyl prodrugs, being labile under basic and hydrogenation conditions. Therefore, we choose to protect the hydroxylamine by a 2,4-dimethoxybenzyl (DMB) group, which can be removed in mild conditions (1–2% TFA in DCM, 15 min) [22], allowing the introduction of a masked phosphate moiety at the penultimate step of the prodrug synthesis. All synthetic steps are depicted in Scheme 5, Scheme 6 and Scheme 7.

Except for the commercially available saligenol (**11a**), most of the substituted salicyl alcohols (**11b**–**e**) have been prepared in 71–86% yield by standard reduction methods of commercially available salicylic aldehydes or acids except for the two compounds (**11f**–**g**). Salicyl alcohol substituted with trifluoromethyl group (**11g**) has been prepared in two steps from the 2-methoxy-5-(trifluoromethyl)benzoic acid. After demethylation using iodocyclohexane in DMF under reflux, the carboxylic acid is immediately reduced with LiAlH_4_ into the desired compound **11f** with 54% yield over the two steps [23] (Scheme 5).

The binary system reduction NaBH_4_/BF_3_.Et_2_O affords benzyl alcohol **11g** with an excellent yield, 86%, compared to 28% with LiAlH_4_ [24].

The reaction of a phosphorus oxychloride solution in THF at −78 °C with the salicyl alcohols **11** in the presence of trimethylamine (TEA) led to the non-stable *cyclo*Salphosphochloridates **12** (Scheme 5), which were characterized by NMR ^1^H and ^31^P. They were directly used in the next reaction, i.e., condensation with the primary alcohol of hydroxamic and retrohydroxamic acids, without purification.

The precursor **14** of retrohydoxamic acids **9** was prepared by Woo’s method, i.e., activation of alcohol **13** by triflate followed by nucleophilic substitution with the *O*-2,4-dimethoxybenzyl hydroxylamine (Scheme 6). After formylation, compound **15a** was obtained as a mixture of two conformers, *Z* and *E*, in a 30:70 ratio, respectively (determined by NMR spectroscopy), due to the restricted rotation around the C-N bond. Acetylation of **14** led to compound **15b** as the sole *E* conformer. Deprotection of the silylether gave the primary alcohols **9a** and **9b** with the same selectivity as before.

An alternative synthesis has been developed to obtain hydroxamic acids **10**. According to the previously described method, the *O*-protected derivative **10a** was prepared in one step from commercially available β-propiolactone using *O*-2,4-dimethoxybenzyl hydroxylamine in the presence of an excess of LiHMDS (Scheme 6). Compound **10a** was obtained as a mixture of two *Z* and *E* conformers in a 60:40 ratio, respectively [10]. The selective *N*-methylation versus *O*-methylation of **10a** was accomplished under basic conditions with K_2_CO_3_ and methyl iodide, leading to **10b** as a sole *E* conformer (Scheme 6).

The key coupling step of the *O*-protected retrohydroxamic and hydroxamic acids **9** and **10** with the *cyclo*Salphosphochloridate derivatives **12** was carried out in DCM at −40 °C in the presence of trimethylamine (TEA) and a catalytic amount of *N*,*N*-dimethylaminopyridine (DMAP). In these conditions, the precursors **16b** and **17** of the *cyclo*Sal prodrugs were obtained in moderate to reasonable yields with the exception of compound **17bd** (Scheme 7).

**Scheme 8:**
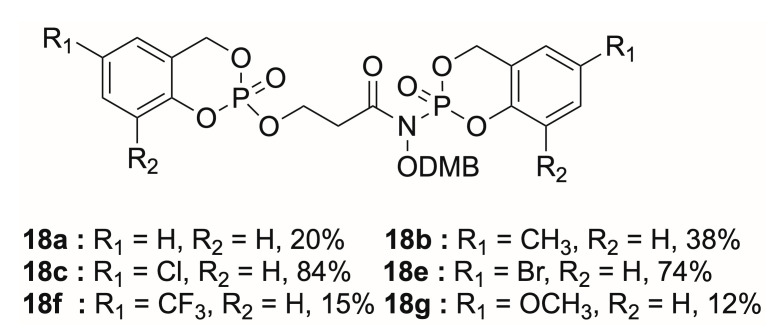
Bis(*cyclo*Sal) compounds **18**.

The coupling of *cyclo*Salphosphochloridate with the hydroxamic acid **10a** was performed in the same conditions (TEA/cat. DMAP), yielding the desired precursors **16a** as a mixture of the two *Z* and *E* conformers in a 60:40 ratio along with a by-product. This latter was identified as bis*cyclo*Sal compound **18**, resulting from the nucleophilic attack of the nitrogen atom of the hydroxamic moiety at the phosphorus atom (Scheme 8). In the presence of an excess of trimethylamine, deprotonation occurs, generating the hydroxamate anion that can react with *cyclo*Salphosphochloridate derivatives **12**, leading to the bis(*cyclo*Sal) compounds in low to modest yield except for **18c** and **18d** isolated in good yields.

The bis(*cyclo*Sal) **18d**, substituted with two chlorine atoms could not be obtained because degradation occurred during the purification of silica. To avoid the formation of **18**, an alternative synthesis has been carried out to obtain the compounds **17aa**–**ag** by replacing TEA with pyridine, a weaker base. The last step is the selective deprotection of the *O*-dimethoxybenzyl group by using 2% or 3% of TFA in DCM [25]. In these conditions, only ten *cyclo*Saligenyl prodrugs **7** and **8** were obtained (Scheme 7) and screened for growth inhibition on *E. coli* and *M. smegmatis*. It was not possible to obtain the unprotected bis(*cyclo*Sal) prodrugs without degradation of the compounds. Accordingly, the antibacterial efficiency of the *O*-protected bis(*cyclo*Sal) prodrugs **18** has been evaluated.

### 2.2. Biological Evaluation

The efficacy of the prodrugs in inhibiting the growth of *E. coli* and *M. smegmatis* was evaluated by the paper disk diffusion method at 800 nanomoles. Isoniazid (30 nanomoles) and fosmidomycin (10 nanomoles) were used as reference compounds for positive *M. smegmatis* and *E. coli* growth inhibition. The diameters of the inhibition zone are given with respect to the amount of inhibitor deposited on the disk (Table 1).

Prodrugs **7** and **8** were tested on an *E. coli* culture. among them only the two 5-Cl-*cyclo*Sal prodrugs (**7ac**, **7bc**) showed growth inhibition in *E. coli*. These two are the only ones to have both an aromatic ring substituted by a halogen and to be retrohydroxamic acids. The growth inhibition induced by prodrugs **7ac** and **7bc** is significantly less effective than the reference compound. In fact, an 80-fold higher amount of prodrugs is required to observe a 2 and 5-fold lower inhibition than fosmidomycin, the most efficient inhibitor of *E. coli* growth (Table 1). This may be due to a degradation of prodrugs **7ac** and **7bc** with the release of the parent molecules, fosfoxacin **3a** and its *N*-acetylated derivative **3b**, respectively, which enter bacteria via glycerol 3-phosphate (GlpT) and/or glucose 6-phosphate (UhpT) transporters. However, compound **7bc** was three and a half times more effective at inhibiting growth in *E. coli* than **7ac**. This result is in accordance with the results observed on *E. coli* DXR for the parent molecules fosfoxacin **3a** and its acetylated analog **3b** [10]. The absence of growth inhibition on *E. coli* of the other prodrugs **7** and **8** seemed to indicate that no release of the drugs occurred outside the bacterial cells in the incubation medium. To explain the inefficiency of this prodrug series on the *E. coli* strain, various hypotheses can be considered. The rigid aromatic ring of the *cyclo*Saligenyl group prevents penetration or at least slows it down considerably. Assuming that the prodrug entered the bacteria, if the release of the parent molecules is too slow, the inhibitor amount will be too low to inhibit the DXR. Since *E. coli* is a fast-growing bacterium, anything that slows down the release of parent inhibitors into bacterial cells should increase the bacterial resistance to these prodrugs.

Prodrugs **7** and **8** were screened for growth inhibition on *M. smegmatis* strain. Only the 5-Cl-*cyclo*Sal prodrugs **7ac**, **8ac** and **8bc** displayed a low antibacterial activity and are much less efficient than the antitubercular reference, isoniazid. To induce a 3.5-fold lower inhibition than the reference, 27-fold more amount of prodrugs is required (Table 1). In Mycobacteria, which do not possess the GlpT and UhpT transporters, the *cylo*sal prodrugs enter into the bacteria by passive diffusion to release the active parent compound. We assume that the prodrugs **7** and **8** penetrate the cell but are probably too stable to be chemically hydrolyzed and therefore deliver too low amounts of the DXR inhibitors to observe bacterial growth. The hydrolysis rate of the prodrugs could be controlled by the substituent present at the C-5 position of the salicylic moiety: an electron-withdrawing substituent such as chlorine accelerates the hydrolysis, whereas an electron-donating group stabilizes the phenol ester bond resulting in a decrease of the hydrolysis rate. The 5-Cl-*cyclo*Sal prodrugs **7ac**, **8ac** and **8bc**, having a lower hydrolytic stability, show the ability to deliver the parent molecules inside the cell. In contrast, the absence of inhibition was observed with the unsubstituted prodrug series (**7aa**–**8aa**, **7ba**–**8ba**) and 5-methyl-*cyclo*Saligenyl prodrug (**8bb**), which could be correlated with higher hydrolytic stability as compared with the 5-Cl-*cyclo*Sal prodrugs (Table 1). The lack of inhibition with the 5-CF_3_-*cyclo*Sal prodrug (**8bf**) is unclear and could be due to its inadequate stability or its short half-life resulting in hydrolysis outside the cells to give the phosphohydroxamic acid **2b**, which cannot enter into *M. smegmatis*. Degradation of the prodrug or active compound by various enzymes or expulsion of the inhibitor out of bacteria by efflux pumps may be envisaged to explain the absence or low inhibition in *M. smegmatis* growth with these *cyclo*Sal prodrugs. Making an assumption that these prodrugs are not lipophilic enough to cross the waxy wall of the mycobacteria, we evaluated the growth inhibition of the double prodrugs. All results are reported in Table 2. Remarkably, the presence of the DMB group greatly enhances the efficiency of the *cyclo*Sal prodrugs in inhibiting the growth of *M. smegmatis*. Indeed, two compounds of the unsubstituted double prodrug series, **17aa** and **16ba** displayed inhibition zones (Table 2, entries 1–4), while the *cyclo*Sal prodrug series were inactive. Compound **16ba** showed nearly the same inhibition as isoniazid but with 27-fold higher amounts of prodrugs. The 5-Cl-*cyclo*Sal double prodrug series, except for the compound **16bc**, shows a 2 to 3-fold higher inhibition compared to their unprotected analogs, demonstrating that the presence of the DMB-protecting group increases the lipophilicity of the prodrugs, thereby promoting the penetration of the DXR inhibitor into the bacteria. This is in accordance with the work reported in the literature in which the protection of (retro)hydroxamate, e.g., *O*-linked aryl/alkyl groups, also increases the lipophilicity [26,27]. The 5-CH_3_-*cyclo*Sal double prodrug series (Table 2, entries 5–8) and the 5-OCH_3_-*cyclo*Sal double prodrug series (Table 2, entries 25–28) remain inactive despite the presence of DMB. This is probably due to phosphate release rather than diffusion through the bacterial wall. In fact, these compounds with electron-donating group substituents slow down the rate of drug release, resulting in a very low concentration of the drug, preventing bacterial growth.

Compared to the isoniazid inhibition zones, the best results were obtained with the 5-halogeno-*cyclo*Sal double prodrug series (Table 2, entries 10–12 and 17–20) and the 5-CF_3_-*cyclo*Sal double prodrug series (Table 2, entries 21–24) which allow a more efficient release of the parent compounds due to the presence of the electron-withdrawing substituent in *para* position of the phenolic phosphate ester bond. The loss of activity with the double prodrugs bearing two chlorine atoms at C-3 and C-5 (Table 2, entries 14–16) could be explained by the instability of these compounds and their partial hydrolysis already outside the cell. These mechanisms release the active molecule into the extracellular medium, thereby reducing the concentration of intracellular inhibitors.

It should be noted that for each series, the double prodrugs releasing phosphohydroxamic acid **4a** were inactive (Table 2, entries 3, 11, 15, 23) or only very weakly active (Table 2, entry 19) on *M. smegmatis* growth inhibition. The prodrugs releasing *N*-methylphosphohydroxamic acid **4b** (Table 2, entries 4, 12, 16, 20, 24) displayed the best antimycobacterial activity. These results are not surprising as we previously reported that compound **4a** inhibited the DXR of *M. smegmatis* as well as the fosfoxacin **3a** with IC_50_ values in the micromolar range. In contrast, the *N*-methylated derivatives **3b** and **4b** were the most effective inhibitors with IC_50_ values in the nanomolar range.

Bis(*cyclo*Saligenyl) prodrugs **18** were also tested on *E. coli* and *M. smegmatis* strains. As anticipated, none of the compounds was able to inhibit *E. coli* growth, the consequence of a lack of uptake. Concerning *M. smegmatis*, as previously mentioned, the Bis(*cyclo*Saligenyl) prodrugs bearing an electron-withdrawing substituent at C-5 (**18c** and **18e**–**f**) displayed an antimycobacterial activity, whereas the unsubstituted one (**18a**) or with electron-donating substituents (**18a**–**b** and **18g**) were inactive (Table 3). A clear correlation was observed between the efficacy of the *M. smegmatis* growth inhibition and the substitution of the saligenyl moiety as well as it was observed with the antiviral *cyclo*Sal pronucleotides developed by C. Meier.

## 3. Experimental Section

### 3.1. Chemistry

#### 3.1.1. General Methods

All non-aqueous reactions were run in dry solvents under an argon atmosphere. Commercial-grade reagents were purchased from Sigma-Aldrich (Burlington, MA, USA) or Acros Organics (Geel, Belgium) and used without further purification. Petroleum ether 40–60 °C (Sigma-Aldrich) was used for purification. Flash chromatography was performed on silica gel 60 (230–400) mesh with the solvent system as indicated. Automated flash chromatography was performed on a Combiflash^®^ Rf™ (Serlabo Technologies, Entraigues-sur-la-Sorgue, France) or on a Puriflash^®^ 215 (Interchim, Montluçon, France). TLC plates were revealed under UV light (254 nm) and/or by spraying with an ethanolic solution of phosphomolybdic acid (20%) or an ethanolic solution of potassium permanganate followed by heating.

The NMR spectra were recorded on a BRUKER Avance 300 (^1^H-NMR: 300 MHz; ^13^C-NMR, 75.5 MHz; ^31^P-NMR 121.5 MHz; ^19^F-NMR 282.4 MHz) or a BRUKER Avance 400 (^1^H-NMR: 400 MHz; ^13^C-NMR, 100.6 MHz; ^31^P-NMR 162 MHz) or a BRUKER Avance 500 (^1^H-NMR: 500 MHz; ^13^C-NMR, 125.8 MHz). ^1^H-NMR experiments were performed in CDCl_3_, D_2_O, CD_3_OD in CDCl_3_ with CHCl_3_ (δ = 7.26 ppm), DHO (δ = 4.79 ppm), CD_2_HOD (δ = 3.31 ppm) as internal references. ^13^C-NMR experiments were performed in CDCl_3_ with CDCl_3_ (δ = 77.23 ppm), CD_2_HOD (δ = 49.0 ppm) as internal references. For ^31^P-NMR reference, the spectrometer had an external reference corresponding to 80% phosphoric acid in D_2_O (δ = 0 ppm). The chemical shifts (δ) are expressed in ppm. s, d, t, q, or bs are abbreviations for multiplicity and correspond to singlet, doublet, triplet and quadruplet or broad singlet. J-couplings are exposed in Hz.

Most of the hydroxamate are present as two *Z* and *E* conformers in equilibrium. If only one signal is described, it is common to all conformers. The evaluation of the relative amount of the conformers was made by integration of the CH_2_CO or CH_2_N or OCH_2_DMB proton signals.

Negative or positive-mode electrospray MS was performed on a Bruker Daltonics microTOF spectrometer (Bruker Daltonik GmbH, Bremen, Germany) equipped with an orthogonal electrospray (ESI) interface. Calibration was performed using a solution of 10 mM sodium formate. Sample solutions were introduced into the spectrometer source with a syringe pump (Harvard type 55–1111: Harvard Apparatus Inc., South Natick, MA, USA) with a flow rate of 5 μL min^−1^.

##### General Procedure A—Reduction of Carboxylic Acid to Alcohol

A solution of carboxylic acid (1 equiv.) in dry THF (9 mL/mmol) at 0 °C was treated with a 1 M solution of LiAlH_4_ in THF (1.2 mL/mmol). The reaction mixture was stirred overnight at room temperature. The reaction mixture was quenched by the addition of water. The solvent was evaporated under reduced pressure, and diethyl ether was added. The organic layer was washed with a 10% aqueous solution of HCl. The aqueous layer was then saturated with NaCl and washed several times with diethyl ether. The organic layers were collected, dried over anhydrous Na_2_SO_4_, filtered and evaporated to dryness under reduced pressure.

##### General Procedure B—Synthesis of *cyclo*Salphosphochloridate

A solution of the alcohol **11a**–**g** (1 equiv.) and triethylamine (2.1 equiv.) in THF (1.9 mL/mmol) was added dropwise to a stirred solution of P(O)Cl_3_ (1.1 equiv) in THF (1.4 mL/mmol) at −78 °C. The reaction mixture was stirred overnight at room temperature. The triethylammonium chloride was filtered, and the solvent was removed under reduced pressure. The crude product was not purified by flash chromatography due to the reactivity of the product.

##### General Procedure C—Deprotection of Alcohol

The silylether (1 equiv) in THF (15 mL/mmol) was treated with tetra-*N*-butylammonium fluoride (2 equiv). The reaction mixture was monitored by TLC (EtOAc). The THF was removed under reduced pressure.

##### General Procedure D—Synthesis of *cyclo*Salphosphostriester

To a solution of alcohol (1 equiv), triethylamine (1.1 equiv.), DMAP (0.5 equiv.) in dry DCM (3 mL/mmol) was treated with a solution of *cyclo*Salphosphochloridate **12a**–**g** (3 equiv.) in DCM (0.80 mL/mmol) at −40 °C. The resulting mixture was warmed up to room temperature and stirred overnight. A saturated aqueous solution of NH_4_Cl was added, and the aqueous layer was extracted with DCM. The organic layers were collected, dried over anhydrous Na_2_SO_4_ and filtered, and solvents were removed under reduced pressure.

##### General Procedure E—Synthesis of *cyclo*Salphosphostriester

The alcohol (1 eq.) was dissolved in anhydrous pyridine (2.8 mL/mmol). The mixture was cooled to −40 °C, and the *cyclo*Salphosphochloridate **12a**–**g** (2 eq.) in toluene (1.1 mL/mmol) was added dropwise. The reaction was stirred to −40 °C for 30 min and warmed up to room temperature overnight. The pyridine was evaporated under reduced pressure.

##### General Procedure F—Deprotection of Dimethoxybenzyle

The protected product (1 equiv) was stirred at room temperature in a solution at 2% or 3% of TFA in DCM (2 equiv). The reaction was monitored by TLC (EtOAc). The dichloromethane was removed under reduced pressure, and the mixture was suspended in a minimum of anhydrous methanol, filtered and evaporated under reduced pressure.

*2-(Hydroxymethyl)-4-methylphenol* (**11b**). The general procedure A was applied to synthesize the compound **11b** from the corresponding carboxylic acid (1.0 g, 6.57 mmol). The crude product was purified by flash chromatography (EtOAc/petroleum ether, 35:65 → EtOAc/petroleum ether, 75:25) to give **11b** as a colorless solid (695 mg, 77%). Rf = 0.57 (EtOAc/petroleum ether, 3:7); ^1^H-NMR (500 MHz, CDCl_3_): 2.45 (3H, s, CH_3_), 5.01 (2H, d, ^4^*J* = 4.7 Hz, OCH_2_Ph), 6.98 (1H, d, ^3^*J* = 8.0 Hz, CH_Ar_), 7.04 (1H, s, CH_Ar_), 7.20 (1H, bdd, ^3^*J* = 8.2 Hz, ^4^*J* = 1.6 Hz, CH_Ar_); ^13^C-NMR (125.8 MHz, CDCl_3_): 20.6 (CH_3_), 64.9 (OCH_2_Ph), 116.5 (CH_Ar_), 124.6 (C_Ar_), 128.6 (CH_Ar_), 129.5 (C_Ar_), 130.1 (CH_Ar_), 153.9 (C_Ar_OH).
*4-Chloro-2-(hydroxymethyl)phenol* (**11c**). The general procedure A was applied to synthesize the compound **11c** from the corresponding carboxylic acid (1.0 g, 5.79 mmol). The crude product was purified by flash chromatography (EtOAc/petroleum ether, 20:80 → EtOAc/petroleum ether, 30:70) to give **11c** as a colorless solid (1.32 g, 88%). Rf = 0.43 (EtOAc/petroleum ether, 2:8; ^1^H-NMR (500 MHz, CDCl_3_): 4.83 (2H, s, OCH_2_Ph), 6.82 (1H, d, ^3^*J* = 8.6 Hz, CH_Ar_), 7.02 (1H, d, ^4^*J* = 2.4 Hz, CH_Ar_), 7.20 (1H, dd, ^3^*J* = 8.7 Hz, ^4^*J* = 2.6 Hz, CH_Ar_); ^13^C-NMR (125.8 MHz, CDCl_3_): 64.4 (OCH_2_Ph), 118.2 (CH_Ar_), 124.9 (C_Ar_), 126.1 (C_Ar_), 127.6 (CH_Ar_), 129.9 (CH_Ar_), 154.9 (C_Ar_OH).
*2,4-Dichloro-6-(hydroxymethyl)phenol* (**11d**). The general procedure A was applied to synthesize the compound **11d** from the corresponding carboxylic acid (2.20 g, 10.6 mmol). The crude product was purified by flash chromatography (EtOAc/petroleum ether, 20:80) to give **11d** as a colorless solid (1.45 g, 71%). Rf = 0.53 (EtOAc/petroleum ether, 2:8); ^1^H-NMR (400 MHz, CDCl_3_): 4.77 (2H, s, OCH_2_Ph), 7.12 (1H, d, ^4^*J* = 2.4 Hz, CH_Ar_), 7.28 (1H, d, ^4^*J* = 2.4 Hz, CH_Ar_); ^13^C-NMR (125.8 MHz, CDCl_3_): 62.4 (OCH_2_Ph), 121.2 (C_Ar_), 125.4 (C_Ar_), 126.9 (CH_Ar_), 128.4 (CH_Ar_), 128.6 (C_Ar_), 149.1 (C_Ar_OH).
*4-Bromo-2-(hydroxymethyl)phenol* (**11e**). The general procedure A was applied to synthesize the compound **11e** from the corresponding carboxylic acid (1.76 g, 8.10 mmol). The crude product was purified by flash chromatography (EtOAc/petroleum ether, 20:80 → EtOAc/petroleum ether, 40:60) to give **11e** as a colorless solid (1.21 g, 74%). Rf = 0.47 (EtOAc/petroleum ether, 2:8); ^1^H-NMR (500 MHz, CD_3_OD): 4.60 (2H, s, OCH_2_Ph), 6.68 (1H, d, ^3^*J* = 8.6 Hz, CH_Ar_), 7.18 (1H, dd, ^3^*J* = 8.5 Hz, ^4^*J* = 2.5 Hz, CH_Ar_), 7.40 (1H, d, ^4^*J* = 2.5 Hz, CH_Ar_); ^13^C-NMR (125.8 MHz, CD_3_OD): 60.3 (OCH_2_Ph), 112.2 (C_Ar_), 117.6 (CH_Ar_), 131.5 (CH_Ar_), 131.6 (C_Ar_), 131.8 (CH_Ar_), 155.4 (C_Ar_OH).
*2-(Hydroxymethyl)-4-(trifluoromethyl)phenol* (**11f**) Step1: Iodocyclohexane (5 mL, 45.4 mmol) was added to a solution of 2-(methoxy)-5-(trifluoromethyl)benzoic acid (1 g, 4.54 mmol) in DMF (5 mL). The mixture was refluxed for 4 h, and then the DMF was evaporated under reduced pressure. The resulting oil was dissolved in DCM (40 mL) and washed with a saturated aqueous solution of NaHCO_3_ (2 × 50 mL). The aqueous layer was acidified with a 10% HCl solution until a pH = 2 was obtained. The aqueous layer was then extracted with DCM (3 × 70 mL). The organic layer was dried over anhydrous Na_2_SO_4_, filtered and evaporated to dryness under reduced pressure. The crude product (1.95 g) was not purified and directly reduced with LiAlH_4_. Rf = 0.68 (EtOAc/petroleum ether, 5:5); ^1^H-NMR (400 MHz, CD_3_OD): 7.05 (1H, d, ^3^*J* = 8.6 Hz, CH_Ar_), 7.74 (1H, dd, ^3^*J* = 8.8 Hz, ^4^*J* = 2.2 Hz, CH_Ar_), 8.13 (1H, d, ^4^*J* = 2.0 Hz, CH_Ar_); ^13^C-NMR (125.8 MHz, CD_3_OD): 114.3 (C_Ar_), 119.5 (CH_Ar_), 122.5 (q, ^2^*J*_C-F_ = 32.9 Hz, *C*_Ar_CF_3_), 125.6 (q, ^1^*J*_C-F_ = 269.4 Hz, C_Ar_*C*F_3_), 129.1 (q, ^3^*J*_C-F_ = 4.0 Hz, CH_Ar_), 133.1 (q, ^3^*J*_C-F_ = 3.0 Hz, CH_Ar_), 165.9 (C_Ar_), 172.6 (CO); ^19^F-NMR (282.4 MHz, CD_3_OD): −64.4.

Step 2: The general procedure A was applied to synthesize the compound **11f** from the freshly prepared carboxylic acid (see step 1) (935 mg, 4.54 mmol). The crude product was purified by flash chromatography (EtOAc/petroleum ether, 20:80 → EtOAc/petroleum ether, 40:60) to give **11f** as a colorless solid (473 mg, 54%). Rf = 0.47 (EtOAc/petroleum ether, 2:8); ^1^H-NMR (400 MHz, CDCl_3_): 4.94 (2H, s, OCH_2_Ph), 6.96 (1H, d, ^3^*J* = 8.5 Hz, CH_Ar_), 7.30 (1H, s, CH_Ar_), 7.47 (1H, dd, ^3^*J* = 8.5 Hz, ^4^*J* = 1.9 Hz, CH_Ar_), 7.80 (1H, d, C_Ar_OH); ^13^C-NMR (125.8 MHz, CDCl_3_): 64.7 (OCH_2_Ph), 117.2 (CH_Ar_), 122.5 (q, ^2^*J*_C-F_ = 33.1 Hz, *C*_Ar_CF_3_), 124.6 (C_4°_), 125.1 (q, ^3^*J*_C-F_ = 3.3 Hz, CH_Ar_), 127.0 (q, ^3^*J*_C-F_ = 3.6 Hz, CH_Ar_), 159.3 (C_Ar_); ^19^F-NMR (282.4 MHz, CDCl_3_): −62.5.

*2-(Hydroxymethyl)-4-methoxyphenol* (**11g**). To a solution of 2-hydroxy-5-methoxybenzoic acid (3 g, 17.8 mmol), NaBH_4_ (1.55, 41 mmol) in THF (47 mL) was added a solution of BF_3_-Et_2_O (3.3 mL, 26.7 mmol) in THF (12 mL) and refluxed overnight. The reaction mixture was cooled, poured into H_2_O (50 mL) and extracted with EtOAc (3 × 100 mL). The aqueous layer was saturated with NaCl and extracted with EtOAc (3 × 100 mL). The organic layers were collected, dried over anhydrous Na_2_SO_4_, filtered and concentrated under reduced pressure. The crude product was purified by automated chromatography (petroleum ether → EtOAc/petroleum ether, 5:5) to give a colorless solid (2.18 g, 86%). Rf = 0.38 (EtOAc/petroleum ether, 3:7); ^1^H-NMR (500 MHz, CDCl_3_): 3.75 (3H, s, OCH_3_), 4.81 (2H, s, OCH_2_Ph), 6.61 (1H, d, ^4^*J* = 2.9 Hz, CH_Ar_), 6.76 (1H, dd, ^3^*J* = 9.0 Hz, ^4^*J* = 2.7 Hz, CH_Ar_), 6.81 (1H, d, ^3^*J* = 8.7 Hz, CH_Ar_); ^13^C-NMR (125.8 MHz, CDCl_3_): 56.0 (OCH_3_), 64.9 (OCH_2_Ph), 113.6 (CH_Ar_), 114.6 (CH_Ar_), 117.4 (CH_Ar_), 125.6 (C_Ar_), 150.0 (C_Ar_), 153.3 (C_Ar_).
*2-Chloro-4H-benzo[d][1,3,2]dioxaphosphinine 2-oxide* (**12a**). The product was obtained as a colorless oil according to general procedure B. Rf = 0.91 (EtOAc/petroleum ether, 5:5); ^1^H-NMR (400 MHz, CDCl_3_): 5.44–5.53 (2H, m, OCH_2_Ph), 7.11 (2H, dd, ^3^*J* = 7.9 Hz, ^3^*J* = 7.9 Hz, CH_Ar_), 7.23 (1H, dd, ^3^*J* = 7.9 Hz, ^3^*J* = 7.7 Hz, CH_Ar_), 7.37 (1H, dd, ^3^*J* = 7.9 Hz, ^3^*J* = 7.9 Hz, CH_Ar_); ^31^P-NMR (162.0 MHz, CDCl_3_): −6.03.
*2-Chloro-6-methyl-4H-benzo[d][1,3,2]dioxaphosphinine 2-oxide* (**12b**). The product was obtained as a slightly yellow oil according to general procedure B. Rf = 0.93 (EtOAc/petroleum ether, 5:5); ^1^H-NMR (400 MHz, CDCl_3_): 2.34 (3H, s, CH_3_), 5.39–5.48 (2H, m, OCH_2_Ph), 6.91 (1H, s, CH_Ar_), 6.98 (1H, d, ^3^*J* = 8.4 Hz, CH_Ar_), 7.15 (1H, d, ^3^*J* = 8.4 Hz, CH_Ar_); ^31^P-NMR (162.0 MHz, CDCl_3_): −5.84.
*2,6-Dichloro-4H-benzo[d][1,3,2]dioxaphosphinine 2-oxide* (**12c**). The product was obtained as a colorless oil according to general procedure B. Rf = 0.88 (EtOAc/petroleum ether, 3:7); ^1^H-NMR (400 MHz, CDCl_3_): 5.40–5.50 (2H, m, OCH_2_Ph), 6.91 (1H, d, ^3^*J* = 8.7 Hz, CH_Ar_), 7.13 (1H, d, ^4^*J* = 2.4 Hz, CH_Ar_), 7.33–7.36 (1H, m, CH_Ar_); ^31^P-NMR (162.0 MHz, CDCl_3_): −6.66.
*2,6,8-Trichloro-4H-benzo[d][1,3,2]dioxaphosphinine 2-oxide* (**12d**). The product was obtained as a yellow oil according to general procedure B. Rf = 0.33 (EtOAc/petroleum ether, 15:85); ^1^H-NMR (400 MHz, CDCl_3_): 5.41–5.49 (2H, m, OCH_2_Ph), 7.05 (1H, m, CH_Ar_), 7.46 (1H, s, CH_Ar_); ^31^P-NMR (162.0 MHz, CDCl_3_): −6.81.
*6-Bromo-2-chloro-4H-benzo[d][1,3,2]dioxaphosphinine 2-oxide* (**12e**). The product was obtained as a yellow oil according to general procedure B. ^1^H-NMR (300 MHz, CDCl_3_): 5.38–5.54 (2H, m, OCH_2_Ph), 7.00 (1H, d, ^3^*J* = 8.7 Hz, CH_Ar_), 7.27–7.28 (1H, s, CH_Ar_), 7.46–7.50 (1H, m, CH_Ar_); ^31^P-NMR (121.5 MHz, CDCl_3_): −6.48.
*2-Chloro-6-(trifluoromethyl)-4H-benzo[d][1,3,2]dioxaphosphinine 2-oxide* (**12f**). The product was obtained as a yellow oil according to general procedure B. ^1^H-NMR (400 MHz, CDCl_3_): 5.49–5.58 (2H, m, OCH_2_Ph), 7.24 (1H, d, ^3^*J* = 8.6 Hz, CH_Ar_), 7.43 (1H, s, CH_Ar_), 7.66 (1H, d, ^3^*J* = 8.2 Hz, CH_Ar_); ^31^P-NMR (162.0 MHz, CDCl_3_): −6.99; ^19^F-NMR (282.4 MHz, CDCl_3_): −63.4.
*2-Chloro-6-methoxy-4H-benzo[d][1,3,2]dioxaphosphinine 2-oxide* (**12g**). The product was obtained as a yellow oil according to general procedure B. ^1^H-NMR (400 MHz, CDCl_3_): 5.40–5.50 (2H, m, OCH_2_Ph), 6.60 (1H, d, ^4^*J* = 3.3 Hz, CH_Ar_), 6.87–6.89 (1H, m, CH_Ar_), 7.03 (1H, d, ^3^*J* = 8.9 Hz, CH_Ar_); ^31^P-NMR (121.5 MHz, CDCl_3_): −5.87.
*O-(2,4-Dimethoxybenzyl)hydroxylamine* Step 1: *N*-hydroxyphtalimide (5.00 g, 29.6 mmol) and 2,4-dimethoxybenzyl alcohol (4.96 g, 30.4 mmol) were stirred in dichloromethane (220 mL) at 0 °C. Triphenyl phosphine (12.2 g, 46.5 mmol) was added, followed by diisopropylazodicarboxylate (9.0 mL, 45.7 mmol). The resulting solution was stirred at room temperature for 24 h. The dichloromethane was removed under reduced pressure, and the resulting oil was recrystallized in boiling ethanol (200 mL) to give colorless crystals (6.32 g, 68%). Rf = 0.55 (EtOAc/petroleum ether, 3:7); ^1^H-NMR (500 MHz, CDCl_3_): 3.72 (3H, s, OCH_3_), 3.79 (3H, s, OCH_3_), 5.21 (2H, s, CH_2_), 6.39–6.44 (2H, m, Ar-H), 7.31 (1H, d, ^3^*J* = 8.4 Hz, Ar-H), 7.70–7.79 (4H, m, Ar-H); ^13^C-NMR (75.5MHz, CDCl_3_): 55.4 (OCH_3_), 55.7 (OCH_3_), 74.5 (CH_2_), 98.6–134.3 (CH_Ar_ and C_Ar_), 160.1 (H_3_CO-*C*_Ar_), 162.4 (H_3_CO-*C*_Ar_), 163.7 (C=O); MS (EI)^+^: *m*/*z* calculated for C_17_H_15_NO_5_Na [M + Na]^+^ 336.08, found 336.08.

Step 2: *N*-(2,4-dimethoxybenzyloxy)phtalimide previously synthesized (3 g, 9.6 mmol) was stirred in refluxing ethanol (100 mL). *N*-methylhydrazine was added, and the resulting mixture was stirred at reflux for 1 h. The ethanol was removed under reduced pressure, and the ether was added. The mixture was stirred for 30 min. The solid was filtered, and the solvent was removed to give an oil (1.93 g) slightly contaminated by phtalimide. Rf = 0.52 (EtOAc/petroleum ether, 5:5); ^1^H-NMR (500 MHz, CDCl_3_): 3.79 (3H, s, OCH_3_), 3.80 (3H, s, OCH_3_), 4.66 (2H, s, CH_2_), 5.38 (2H, bs, NH_2_), 6.44–6.46 (2H, m, Ar-H), 7.22 (1H, d, ^3^*J* = 8.8 Hz, Ar-H); ^13^C-NMR (75.5 MHz, CDCl_3_): 55.5 (OCH_3_), 55.7 (OCH_3_), 73.1 (CH_2_), 98.8–131.8 (CH_Ar_ and C_Ar_), 159.3 (H_3_CO-*C*_Ar_), 161.3 (H_3_CO-*C*_Ar_).

*2-((t-Butyldimethylsilyl)oxy)ethan-1-ol* (**13**). Sodium hydride (475 mg, 18.2 mmol) was suspended in THF (36 mL). The resulting mixture was cooled to 0 °C, the ethylene glycol (1 mL, 17.7 mmol) was added dropwise, and the mixture was stirred for 1 h. A solution of *t*-butyldimethylsilyl chloride (2.74 g, 18.0 mmol) in THF (8 mL) was added over a period of 10 min. The resulting mixture was stirred for 4 h at room temperature. A saturated aqueous solution of NaHCO_3_ (40 mL) was added, and the mixture was extracted with EtOAc (2 × 40 mL). The aqueous layer was saturated with NaCl and extracted with EtOAc (2 × 40 mL). The collected organic layers were dried over anhydrous Na_2_SO_4_, filtered, and solvents were removed under reduced pressure. The crude was purified by flash chromatography (EtOAc/petroleum ether, 2:8) to give the compound **13** as a colorless oil (2.48 g, 80%). Rf = 0.25 (EtOAc/petroleum ether, 1:9); ^1^H-NMR (300 MHz, CDCl_3_): 0.08 (6H, s, Si-CH_3_), 0.91 (9H, s, Si-*t*-Bu), 1.99 (1H, bs, OH), 3.62–3.65 (2H, m, CH_2_OTBDMS), 3.70–3.73 (2H, m, CH_2_OH); ^13^C-NMR (75.5 MHZ, CDCl_3_): −5.1 (Si-CH_3_), 18.5 (C_4°_ of *t*-Bu), 26.1 (CH_3_ of *t*Bu), 63.9 (CH_2_OTBDMS), 64.3 (CH_2_OH).
*N-(2-((t-butyldimethylsilyl)oxy)ethyl)-O-(2,4-dimethoxybenzyl)* (**14**). To a solution of **13** (500 mg, 2.8 mmol) in DCM (35 mL), 2,6-lutidine (0.40 mL, 3.4 mmol) was added. The solution was cooled down to −78 °C, and the trifluoromethanesulfonic anhydride (470 μL, 2.8 mmol) was added dropwise. The resulting mixture was stirred at −78 °C for 1 h, and *O*-(2,4-dimethoxybenzyl)hydroxylamine (770 mg, 4.2 mmol) in DCM (20 mL) was added dropwise. The solution was stirred at −78 °C for 1 h, warmed up to room temperature, and stirred for another 2 h. The reaction mixture was diluted with DCM (30 mL) and washed with a saturated aqueous solution of NH_4_Cl (60 mL), a saturated solution of NaHCO_3_ (60 mL), water (60 mL) and brine (60 mL). The organic layer was dried over anhydrous Na_2_SO_4_ and filtered, and the solvents were removed under reduced pressure to give a pale yellow oil. The crude was purified by flash chromatography (petroleum ether → EtOAc/petroleum ether, 15:85) to give the product **14** as a colorless oil (424 mg, 44%). Rf = 0.46 (EtOAc/petroleum ether, 1:9); ^1^H-NMR (300 MHz, CDCl_3_): 0.04 (6H, s, Si-CH_3_), 0.87 (9H, s, *t*Bu), 3.03 (2H, t, ^3^*J* = 5.3 Hz, CH_2_N), 3.73 (2H, t, ^3^*J* = 5.3 Hz, CH_2_OTBDMS), 3.80 (3H, s, OCH_3_), 3.81 (3H, s, OCH_3_), 4.70 (2H, s, OCH_2_DMP), 6.45–6.48 (2H, m, CH_Ar_), 7.23 (1H, m, CH_Ar_); ^13^C-NMR (75.5 MHZ, CDCl_3_): −5.2 (Si-CH_3_), 18.5 (C_4°_ of *t*Bu), 26.1 (CH_3_ of *t*Bu), 53.9 (CH_2_N), 55.6 (OCH_3_), 55.7 (OCH_3_), 59.6 (CH_2_OTBDMS), 70.1 (OCH_2_DMP), 98.7 (CH_Ar_), 104.0 (CH_Ar_), 118.6 (C_Ar_), 131.5 (CH_Ar_), 159.1 (*C*_Ar_OCH_3_), 161.1 (*C*_Ar_OCH_3_); MS (EI)^+^: *m*/*z* calculated for C_17_H_31_NO_4_SiNa [M + Na]^+^ 364.19 found 364.19.
*N-(2-((t-Butyldimethylsilyl)oxy)ethyl)-N-((2,4-dimethoxybenzyl)oxy)formamide* (**15a**). A solution of formic acid (1.11 mL, 29.5 mmol) and acetic anhydride (0.56 mL, 5.9 mmol) was stirred at room temperature for 30 min. The solution was then cooled to 0 °C, and a solution of protected hydroxylamine **14** (200 mg, 0.59 mmol) in a minimum of THF was added dropwise. The reaction mixture was stirred at 0 °C for 10 min, allowed to warm up at room temperature, and stirred overnight. The reaction mixture was diluted with EtOAc, and the organic layer was washed twice with water and twice with a 0.1 M aqueous solution of KOH. The organic layer was dried over anhydrous Na_2_SO_4_, filtered and evaporated to dryness. The crude product was purified by flash chromatography (EtOAc/petroleum ether, 2:8) to give **15a** as a colorless oil (180 mg, 83%) and as a mixture of the two *Z* and *E* conformers in a 30:70 ratio. Rf = 0.43 (EtOAc/petroleum ether, 2:8); ^1^H-NMR (300 MHz, CDCl_3_): 0.06 (6H, s, Si-CH_3_), 0.88 (9H, s, *t*Bu), 3.44–3.83 (10H, m, OCH_2_CH_2_N and OCH_3_), 4.86 (7/10H of 2H, bs, OCH_2_DMP), 4.96 (3/10H of 2H, bs, OCH_2_DMP), 6.45–6.47 (2H, m, CH_Ar_), 7.17 (1H, d, ^2^*J* = 7.0 Hz, CH_Ar_), 7.92 (3/10H of 1H, bs, CHO), 8.19 (7/10H of 1H, bs, CHO); ^13^C-NMR (75.5 MHZ, CDCl_3_): −5.2 (Si-CH_3_), 18.5 (C_4°_ of *t*Bu), 26.1 (CH_3_ of *t*Bu), 47.4 (CH_2_N), 51.8 (CH_2_N), 55.6 (OCH_3_), 55.7 (OCH_3_), 58.8 (CH_2_OTBDMS), 59.3 (CH_2_OTBDMS), 71.4 (OCH_2_DMP), 72.9 (OCH_2_DMP), 98.8 (CH_Ar_), 104.3 (CH_Ar_), 115.4 (C_Ar_), 133.0 (CH_Ar_), 159.0 (CHO), 159.7 (*C*_Ar_OCH_3_), 162.2 (*C*_Ar_OCH_3_), 163.6 (CHO); MS (EI)^+^: *m*/*z* calculated for C_18_H_31_NO_5_SiNa [M + Na]^+^ 392.19, found 392.18.
*N-(2-((t-Butyldimethylsilyl)oxy)ethyl)-N-((2,4-dimethoxybenzyl)oxy)acetamide* (**15b**). To a solution of *N*-H hydroxylamine **14** (116 mg, 0.34 mmol) in acetic anhydride (4 mL/mmol) was added dropwise pyridine (0.08 mL, 1.02 mmol equiv). The reaction mixture was stirred at room temperature overnight. The solvent was evaporated to dryness under reduced pressure. The product **15b** was obtained without purification as a colorless oil (128 mg, 98%) and as the sole *E* conformer. Rf = 0.29 (EtOAc/petroleum ether, 1:9); ^1^H-NMR (300 MHz, CDCl_3_): 0.05 (6H, s, Si-CH_3_), 0.88 (9H, s, *t*Bu), 2.83 (3H, s, COCH_3_) 3.78–3.83 (10H, m, OCH_2_CH_2_N and OCH_3_), 4.83 (2H, s, OCH_2_DMP), 6.45–6.48 (2H, m, CH_Ar_), 7.17 (1H, d, ^2^*J* = 9.0 Hz, CH_Ar_); ^13^C-NMR (75.5 MHZ, CDCl_3_): −5.2 (Si-CH_3_), 18.5 (C_4°_ of *t*Bu), 22.4 (CO*C*H_3_), 26.1 (CH_3_ of *t*Bu), 49.1 (CH_2_N), 55.5 (OCH_3_), 55.6 (OCH_3_), 59.6 (CH_2_OTBDMS), 71.4 (OCH_2_DMP), 98.8 (CH_Ar_), 104.3 (CH_Ar_), 115.7 (C_Ar_), 132.7 (CH_Ar_), 159.6 (*C*_Ar_OCH_3_), 162.1 (*C*_Ar_OCH_3_), 166.6 (*C*OCH_3_); MS (EI)^+^: *m*/*z* calculated for C_19_H_33_NO_5_SiNa [M + Na]^+^ 406.20, found 406.20.
*N-((2,4-Dimethoxybenzyl)oxy)-N-(2-hydroxyethyl)formamide* (**9a**). The general procedure C was applied to synthesize the compound **9a** from protected alcohol **15a** (200 mg, 0.59 mmol). The crude product was purified by flash chromatography (EtOAc) to give **9a** as a colorless oil (97 mg, 76%) and as a mixture of the two *Z* and *E* conformers in a 30:70 ratio. Rf = 0.37 (EtOAc); ^1^H-NMR (500 MHz, CDCl_3_): 2.70 (1H, bs, OH), 3.42–3.84 (10H, m, OCH_2_CH_2_N and OCH_3_), 4.87 (7/10H of 2H, bs, OCH_2_DMP), 5.02 (3/10H of 2H, bs, OCH_2_DMP), 6.45–6.47 (2H, m, CH_Ar_), 7.17 (1H, d, ^2^*J* = 8.1 Hz, CH_Ar_), 7.96 (3/10H of 1H, bs, CHO), 8.22 (7/10H of 1H, bs, CHO); ^13^C-NMR (75.5 MHZ, CDCl_3_): 48.7 (CH_2_N), 53.5 (CH_2_N), 55.6 (OCH_3_), 55.7 (OCH_3_), 60.8 (CH_2_OH), 71.5 (OCH_2_DMP), 73.2 (OCH_2_DMP), 98.8 (CH_Ar_), 104.6 (CH_Ar_), 114.9 (C_Ar_), 132.2 (CH_Ar_), 159.7 (*C*_Ar_OCH_3_), 162.4 (*C*_Ar_OCH_3_), 164.2 (CHO); MS (EI)^+^: *m*/*z* calculated for C_12_H_17_NO_5_Na [M + Na]^+^ 278.0999, found 278.0980.
*N-((2,4-Dimethoxybenzyl)oxy)-N-(2-hydroxyethyl)acetamide* (**9b**). The general procedure C was applied to synthesize the compound **9b** from protected alcohol **15b** (200 mg, 0.59 mmol). The crude product was purified by flash chromatography (EtOAc) to give **9b** as a colorless oil (133 mg, 80%) and as the sole *E* conformer. Rf = 0.48 (EtOAc); ^1^H-NMR (400 MHz, CDCl_3_): 2.13 (3H, s, COCH_3_) 3.80–3.84 (10H, m, OCH_2_CH_2_N and OCH_3_), 4.83 (2H, s, OCH_2_DMP), 6.46–6.49 (2H, m, CH_Ar_), 7.19 (1H, d, ^2^*J* = 9.0 Hz, CH_Ar_); ^13^C-NMR (75.5 MHZ, CDCl_3_): 20.4 (CO*C*H_3_), 50.4 (CH_2_N), 55.6 (OCH_3_), 55.7 (OCH_3_), 61.8 (CH_2_OH), 71.9 (OCH_2_DMP), 98.9 (CH_Ar_), 104.5 (CH_Ar_), 115.1 (C_Ar_), 133.0 (CH_Ar_), 159.7 (*C*_Ar_OCH_3_), 162.4 (*C*_Ar_OCH_3_), 174.2 (*C*OCH_3_); MS (EI)^+^: *m*/*z* calculated for C_13_H_19_NO_5_Na [M + Na]^+^ 292.12, found 292.12.
*N-((2,4-Dimethoxybenzyl)oxy)-3-hydroxypropanamide* (**10a**). A stirred suspension of *O*-protected hydroxylamine (3.06 g, 16.7 mmol) in dry THF (4.5 mL/mmol) at −78 °C was treated with 1 M solution of LiHMDS (55.5 mL, 55.5 mmol). After 1 h, a solution of β-propiolactone (0.70 mL, 11.1 mmol) in a minimum of THF was added. The resulting solution was stirred overnight at room temperature. The reaction was cooled at −78 °C, quenched with a saturated aqueous solution of NH_4_Cl, warmed to room temperature and extracted several times with EtOAc. The collected organic layers were dried over anhydrous Na_2_SO_4_, filtered, and evaporated to dryness under reduced pressure. The crude product was dissolved in THF and treated with TBAF.(H_2_O)_3_ (1.5 equiv). After total consumption of the starting material, the solvent was removed under reduced pressure. The crude product was purified by flash chromatography (EtOAc → EtOAc/MeOH, 90:10) to give the product **10a** (1.45 g, 51%) as a colorless solid and as a mixture of the two *Z* and *E* conformers in a 60:40 ratio. Rf = 0.24 (EtOAc); ^1^H-NMR (500 MHz, CDCl_3_): 2.30 (6/10H of 2H, bs, CH_2_CO), 2.63 (4/10H of 2H, bs, CH_2_CO), 2.91 (1H, bs, OH), 3.79–3.84 (8H, m, OCH_3_ and CH_2_OH), 4.80 (4/10H of 2H, bs, OCH_2_DMP), 4.91 (6/10H of 2H, bs, OCH_2_DMP), 6.44–6.48 (2H, m, CH_Ar_), 7.22 (1H, m, CH_Ar_), 8.16 (4/10 of 1H, bs, NH), 8.58 (6/10 of 1H, bs, NH); ^13^C-NMR (75.5 MHz, CDCl_3_): 33.5 (*C*H_2_CO), 35.6 (*C*H_2_CO), 55.5 (OCH_3_), 55.6 (OCH_3_), 58.1 (CH_2_OH), 58.6 (CH_2_OH), 73.2 (OCH_2_DMP), 74.6 (OCH_2_DMP), 98.6 (CH_Ar_), 104.1 (CH_Ar_), 114.9 (C_Ar_), 155.9 (C_Ar_), 132.8 (CH_Ar_), 159.5 (*C*_Ar_OCH_3_), 161.8 (*C*_Ar_OCH_3_), 162.3 (*C*_Ar_OCH_3_), 169.8 (CO), 176.6 (CO); MS (EI)^+^: *m*/*z* calculated for C_12_H_17_NO_5_Na [M + Na]^+^ 278.10, found 278.10.
*N-((2,4-Dimethoxybenzyl)oxy)-3-hydroxy-N-methylpropanamide* (**10b**). To a solution of **10a** (267 mg, 1.05 mmol), anhydrous K_2_CO_3_ (290 mg, 2.1 mmol) in anhydrous acetone (10.5 mL/mmol) was added iodomethane (0.33 mL, 5.25 mmol). The resulting mixture was refluxed overnight. The mixture was filtered, and acetone was removed under reduced pressure. The resulting oil was dissolved in ether. The organic layer was washed with water, and then the aqueous layer was saturated with NaCl and extracted with EtOAc. The organic layers were collected, dried over anhydrous Na_2_SO_4_, filtered and evaporated. The crude product was purified by automated flash chromatography (EtOAc/petroleum ether, 7:3 → EtOAc) to give **10b** as a yellow oil (243 mg, 86%) as a sole *E* conformer. Rf = 0.44 (EtOAc/petroleum ether, 7:3); ^1^H-NMR (500 MHz, CDCl_3_): 2.63 (2H, t, ^3^*J* = 5.6 Hz, CH_2_CO), 3.23 (4H, ds, NCH_3_ and OH), 3.79–3.84 (8H, m, OCH_3_ and CH_2_OH), 4.80 (2H, s, OCH_2_DMP), 6.46–6.48 (2H, m, CH_Ar_), 7.18 (1H, d, ^3^*J* = 8.9 Hz, CH_Ar_); ^13^C-NMR (75.5 MHz, CDCl_3_): 33.1 (NCH_3_), 34.0 (*C*H_2_CO), 55.6 (OCH_3_), 58.8 (CH_2_OH), 71.1 (OCH_2_DMP), 98.8 (CH_Ar_), 104.4 (CH_Ar_), 115.1 (C_Ar_), 132.9 (CH_Ar_), 159.67 (*C*_Ar_OCH_3_), 162.3 (*C*_Ar_OCH_3_), 174.7 (CO); MS (EI)^+^: *m*/*z* calculated for C_13_H_19_NO_5_Na [M + Na]^+^ 292.12, found 292.12.
*N-((2,4-Dimethoxylbenzyl)oxy)-N-(2-((2-oxido-4H-benzo[d][1,3,2]dioxaphosphinin-2-yl)oxy)ethyl)formamide* (**17a**). The general procedure D was applied to synthesize the compound **17aa** from alcohol **9a** (172 mg, 0.67 mmol). The crude product was purified by flash chromatography (EtOAc/cyclohexane, 8:2) to give **17aa** as a colorless oil (187 mg, 66%) and as a mixture of the two *Z* and *E* conformers in a 20:80 ratio. Rf = 0.49 (EtOAc/cyclohexane, 8:2); ^1^H-NMR (400 MHz, CDCl_3_): 3.57–3.95 (8H, m, NCH_2_ and OCH_3_), 4.38 (2H, bs, POCH_2_), 4.80 (8/10H of 2H, bs, OCH_2_DMP), 4.96 (2/10 of 2H, bs, OCH_2_DMP), 5.25–5.39 (2H, m, ArCH_2_OP), 6.43–6.45 (2H, m, CH_Ar(DMB)_), 7.03 (2H, t, ^3^*J* = 8.2 Hz, CH_Ar(*cyclo*Sal)_), 7.11 (2H, m, CH_Ar(*cyclo*Sal)_ and CH_Ar(DMB)_), 7.28 (1H, m, CH_Ar(*cyclo*Sal)_), 7.87 (2/10H of 1H, bs, CHO), 8.06 (8/10 of 1H, bs, CHO); ^13^C-NMR (125.8 MHz, CDCl_3_): 44.6 (d, ^3^*J*_CP_ = 7.0 Hz, NCH_2_), 55.6 (OCH_3_), 55.7 (OCH_3_), 63.9 (d, ^2^*J*_CP_ = 4.4 Hz, POCH_2_), 69.0 (d, ^2^*J*_CP_ = 6.9 Hz, ArCH_2_OP), 73.2 (OCH_2_DMP), 98.8 (CH_Ar(DMB)_), 104.4 (CH_Ar(DMB)_), 114.9 (C_Ar(DMB)_), 118.9 (d, ^3^*J*_CP_ = 8.7 Hz, *C*H*_Ar_*C_Ar_OP), 120.7 (d, ^3^*J*_CP_ = 8.7 Hz, *C*_Ar_CH_2_OP), 124.5 (CH_Ar(*cyclo*Sal)_), 125.4 (CH_Ar(*cyclo*Sal)_), 129.9 (CH_Ar(*cyclo*Sal)_), 133.1 (CH_Ar(DMB)_), 150.1 (d, ^2^*J*_CP_ = 6.6 Hz, C_Ar_OP), 159.7 (*C*_Ar_OCH_3_), 162.4 (*C*_Ar_OCH_3_), 163.7 (CHO); ^31^P-NMR (162.0 MHz, CDCl_3_): −9.89; MS (EI)^+^: *m*/*z* calculated for C_19_H_22_NO_8_PNa [M + Na]^+^ 446.10, found 446.09.
*N-((2,4-Dimethoxybenzyl)oxy)-N-(2-((6-methyl-2-oxido-4H-benzo[d][1,3,2]dioxaphosphinin-2-yl)oxy)ethyl)formamide* (**17ab**). The general procedure D was applied to synthesize the compound **17ab** from alcohol **9a** (61 mg, 0.24 mmol). The crude product was purified by automated flash chromatography (EtOAc/petroleum ether, 6:4 → EtOAc) to give **17ab** as a colorless oil (30 mg, 46%) and as a mixture of two *Z* and *E* conformers in a 20:80 ratio. Rf = 0.35 (EtOAc/petroleum ether, 7:3); ^1^H-NMR (400 MHz, CDCl_3_): 2.30 (3H, s, CH_3_Ar), 3.78–3.88 (8H, m, NCH_2_ and OCH_3_), 4.35–4.38 (2H, bs, POCH_2_), 4.80 (8/10H of 2H, bs, OCH_2_DMP), 4.97 (2/10 of 2H, bs, OCH_2_DMP), 5.20–5.31 (2H, m, ArCH_2_OP), 6.42–6.46 (2H, m, CH_Ar(DMB)_), 6.83 (1H, bs, CH_Ar(*cyclo*Sal)_), 6.90 (1H, d, ^3^*J* = 8.4 Hz, CH_Ar(*cyclo*Sal)_),7.07 (1H, d, ^3^*J* = 8.4 Hz, CH_Ar(*cyclo*Sal)_), 7.12–7.15 (1H, m, CH_Ar(DMB)_), 7.86 (2/10H of 1H, bs, CHO), 8.07 (8/10 of 1H, bs, CHO); ^13^C-NMR (125.8 MHz, CDCl_3_): 20.9 (CH_3_Ar), 44.7 (d, ^3^*J*_CP_ = 5.8 Hz, NCH_2_), 55.6 (OCH_3_), 55.7 (OCH_3_), 63.9 (POCH_2_), 69.1 (d, ^2^*J*_C-P_ = 7.0 Hz, ArCH_2_OP), 73.2 (OCH_2_DMP), 98.8 (CH_Ar(DMB)_), 104.5 (CH_Ar(DMB)_), 114.9 (C_Ar(DMB)_), 118.7 (d, ^3^*J*_C-P_ = 7.9 Hz, *C*H*_Ar_*C_Ar_OP), 120.3 (d, ^3^*J*_C-P_ = 8.9 Hz, *C*_Ar_CH_2_OP), 125.7 (CH_Ar(*cyclo*Sal)_), 130.4 (CH_Ar(*cyclo*Sal)_), 133.1 (CH_Ar(DMB)_), 134.2 (C_Ar_CH_3_), 148.1 (C_Ar_OP), 159.7 (*C*_Ar_OCH_3_), 162.4 (*C*_Ar_OCH_3_), 163.8 (CHO); ^31^P-NMR (162.0 MHz, CDCl_3_): −9.7; HRMS (EI)^+^: *m*/*z* calculated for C_20_H_24_NO_8_PNa [M + Na]^+^ 460.1132, found 460.1116.
*N-(2-((6-Chloro-2-oxido-4H-benzo[d][1,3,2]dioxaphosphinin-2-yl)oxy)ethyl)-N-((2,4-dimethoxybenzyl)oxy)formamide* (**17ac**). The general procedure D was applied to synthesize compound **17ac** from alcohol **9a** (169 mg, 0.66 mmol). The crude product was purified by automated flash chromatography (EtOAc/cyclohexane, 8:2) to give **17ac** as a colorless oil (155 mg, 51%) and as a mixture of the two *Z* and *E* conformers in a 20:80 ratio. Rf = 0.51 (EtOAc/cyclohexane, 8:2); ^1^H-NMR (400 MHz, CDCl_3_): 3.82–3.88 (8H, m, NCH_2_ and OCH_3_), 4.39 (2H, bs, POCH_2_), 4.80 (8/10H of 2H, bs, OCH_2_DMP), 4.96 (2/10 of 2H, bs, OCH_2_DMP), 5.18–5.34 (2H, m, ArCH_2_OP), 6.43–6.46 (2H, m, CH_Ar(DMB)_), 6.94 (1H, d, ^3^*J* = 8.7 Hz, CH_Ar(*cyclo*Sal)_), 7.03 (1H, s, CH_Ar(*cyclo*Sal)_), 7.12 (1H, d, ^3^*J* = 6.9 Hz, CH_Ar(DMB)_), 7.25 (1H, d, ^3^*J* = 8.8 Hz, CH_Ar(*cyclo*Sal)_) 7.89 (2/10H of 1H, bs, CHO), 8.07 (8/10 of 1H, bs, CHO); ^13^C-NMR (125.8 MHz, CDCl_3_): 44.6 (d, ^3^*J*_CP_ = 6.0 Hz, NCH_2_), 55.6 (OCH_3_), 55.7 (OCH_3_), 64.2 (POCH_2_), 68.5 (d, ^2^*J*_CP_ = 6.9 Hz, ArCH_2_OP), 73.2 (OCH_2_DMP), 98.8 (CH_Ar(DMB)_), 104.5 (CH_Ar(DMB)_), 114.9 (C_Ar(DMB)_), 120.4 (d, ^3^*J*_CP_ = 9.5 Hz, *C*H*_Ar_*C_Ar_OP), 122.1 (d, ^3^*J*_CP_ = 10.2 Hz, *C*_Ar_CH_2_OP), 125.4 (CH_Ar(*cyclo*Sal)_), 129.7 (C_Ar_Cl), 129.9 (CH_Ar(*cyclo*Sal)_), 133.1 (CH_Ar(DMB)_), 147.7 (d, ^2^*J*_CP_ = 6.4 Hz, C_Ar_OP), 159.7 (*C*_Ar_OCH_3_), 162.4 (*C*_Ar_OCH_3_), 163.7 (CHO); ^31^P-NMR (162.0 MHz, CDCl_3_): −10.4; HRMS (EI)^+^: *m*/*z* calculated for C_19_H_21_ClNO_8_PNa [M + Na]^+^ 480.0556, found 480.0556.
*N-(2-((6,8-Dichloro-2-oxido-4H-benzo[d][1,3,2]dioxaphosphinin-2-yl)oxy)ethyl)-N-((2,4-dimethoxybenzyl)oxy)formamide* (**17ad**). The general procedure D was applied to synthesize compound **17ad** from alcohol **9a** (185 mg, 0.73 mmol). The crude product was purified by flash chromatography (EtOAc/cyclohexane, 8:2) to give 17ad as a colorless oil (177 mg, 49%) and as a mixture of two *Z* and *E* conformers in a 20:80 ratio. Rf = 0.51 (EtOAc/cyclohexane, 8:2); ^1^H-NMR (400 MHz, CDCl_3_): 3.82–3.90 (8H, m, NCH_2_ and OCH_3_), 4.41 (2H, bs, POCH_2_), 4.80 (8/10H of 2H, bs, OCH_2_DMP), 4.94 (2/10 of 2H, bs, OCH_2_DMP), 5.19–5.34 (2H, m, ArCH_2_OP),6.46 (2H, bs, CH_Ar(DMB)_), 6.94 (1H, bs, CH_Ar(*cyclo*Sal)_), 7.14 (1H, m, CH_Ar(DMB)_), 7.35 (1H, bs, CH_Ar(*cyclo*Sal)_) 7.91 (2/10H of 1H, bs, CHO), 8.06 (8/10 of 1H, bs, CHO); ^13^C-NMR (125.8 MHz, CDCl_3_): 44.4 (d, ^3^*J*_CP_ = 6.0 Hz, NCH_2_), 55.6 (OCH_3_), 55.7 (OCH_3_), 64.5 (POCH_2_), 68.4 (d, ^2^*J*_CP_ = 6.9 Hz, ArCH_2_OP), 73.2 (OCH_2_DMP), 98.8 (CH_Ar(DMB)_), 104.5 (CH_Ar(DMB)_), 114.8 (C_Ar(DMB)_), 123.4 (d, ^3^*J*_CP_ = 9.5 Hz, Cl*C_Ar_*C_Ar_OP), 123.7 (CH_Ar(*cyclo*Sal)_) 124.9 (*C*_Ar_CH_2_OP), 129.6 (C_Ar_Cl), 130.2 (CH_Ar(*cyclo*Sal)_), 133.1 (CH_Ar(DMB)_), 145.0 (C_Ar_OP), 159.7 (*C*_Ar_OCH_3_), 162.4 (*C*_Ar_OCH_3_), 163.7 (CHO); ^31^P-NMR (162.0 MHz, CDCl_3_): −10.9; MS (EI)^+^: *m*/*z* calculated for C_19_H_20_Cl_2_NO_8_PNa [M + Na]^+^ 514.02, found 514.02.
*N-(2-((6-Bromo-2-oxido-4H-benzo[d][1,3,2]dioxaphosphinin-2-yl)oxy)ethyl)-N-((2,4-dimethoxybenzyl)oxy)formamide* (**17ae**). The general procedure D was applied to synthesize compound **17ae** from alcohol **9a** (60 mg, 0.24 mmol). The crude product was purified by flash chromatography (EtOAc/petroleum ether, 5:5 → EtOAc/petroleum ether, 7:3) to give **17ae** as a colorless oil (44 mg, 37%) and as a mixture of the two *Z* and *E* conformers in a 20:80 ratio. Rf = 0.57 (EtOAc/petroleum ether, 7:3); ^1^H-NMR (400 MHz, CDCl_3_): 3.76–3.95 (8H, m, NCH_2_ and OCH_3_), 4.38 (2H, bs, POCH_2_), 4.80 (8/10H of 2H, bs, OCH_2_DMP), 4.95 (2/10 of 2H, bs, OCH_2_DMP), 5.22 (1H, dd, ^2^*J* = 14.7 Hz, ^3^*J*_P-H_ = 18.3 Hz, ArCH_2_OP), 5.31 (1H, dd, ^2^*J* = 14.3 Hz, ^3^*J*_P-H_ = 8.0 Hz, ArCH_2_OP), 6.44–6.46 (2H, m, CH_Ar(DMB)_), 6.89 (1H, d, ^3^*J* = 8.7 Hz, CH_Ar(*cyclo*Sal)_), 7.11–7.17 (2H, m, CH_Ar(DMB)_ and CH_Ar(*cyclo*Sal)_), 7.38 (1H, d, ^3^*J* = 8.7 Hz, CH_Ar(*cyclo*Sal)_), 7.89 (2/10H of 1H, bs, CHO), 8.06 (8/10 of 1H, bs, CHO); ^13^C-NMR (125.8 MHz, CDCl_3_): 44.5 (d, ^3^*J*_CP_ = 6.9 Hz, NCH_2_), 55.6 (OCH_3_), 55.7 (OCH_3_), 64.1 (POCH_2_), 64.2 (d, ^2^*J*_C-P_ = 4.5 Hz, POCH_2_) 68.3 (d, ^2^*J*_C-P_ = 6.7 Hz, ArCH_2_OP), 73.2 (OCH_2_DMP), 98.8 (CH_Ar(DMB)_), 104.5 (CH_Ar(DMB)_), 114.8 (C_Ar(DMB)_), 117.0 (C_Ar_Br), 120.7 (d, ^3^*J*_C-P_ = 8.6 Hz, *C*H*_Ar_*C_Ar_OP), 122.6 (d, ^3^*J*_C-P_ = 9.4 Hz, *C*_Ar_CH_2_OP), 128.3 (CH_Ar(*cyclo*Sal)_), 132.8 (CH_Ar(*cyclo*Sal)_), 133.1 (CH_Ar(DMB)_), 149.3 (d, ^2^*J*_C-P_ = 6.5 Hz, C_Ar_OP), 159.7 (*C*_Ar_OCH_3_), 162.4 (*C*_Ar_OCH_3_), 163.7 (CHO); ^31^P-NMR (162.0 MHz, CDCl_3_): −10.5; HRMS (EI)^+^: *m*/*z* calculated for C_19_H_21_BrNO_8_PNa [M + Na]^+^ 524.0080, found 524.0138.
*N-((2,4-Dimethoxybenzyl)oxy)-N-(2-((2-oxido-6-(trifluoromethyl)-4H-benzo[d][1,3,2]dioxaphosphinin-2-yl)oxy)ethyl)formamide* (**17af**). The general procedure D was applied to synthesize the compound **17af** from alcohol **9a** (96 mg, 0.38 mmol). The crude product was purified by automated flash chromatography (EtOAc/cyclohexane, 7:3 → EtOAc/cyclohexane, 8:2) to give **17af** as a colorless oil (98 mg, 52%) and as a mixture of two *Z* and *E* conformers in a 20:80 ratio. Rf = 0.50 (EtOAc/cyclohexane, 8:2); ^1^H-NMR (400 MHz, CDCl_3_): 3.76–3.94 (8H, m, NCH_2_ and OCH_3_), 4.42 (2H, bs, POCH_2_), 4.80 (8/10 of 2H, bs, OCH_2_DMP), 4.96 2/10 of 2H, bs, OCH_2_DMP), 5.31 (1H, dd, ^2^*J* = 14.4 Hz, ^3^*J*_P-H_ = 18.6 Hz, ArCH_2_OP), 5.40 (1H, dd, ^2^*J* = 14.4 Hz, ^3^*J*_P-H_ = 8.7 Hz, ArCH_2_OP), 6.44–6.46 (2H, m, CH_Ar(DMB)_), 7.10–7.13 (2H, m, CH_Ar(DMB)_ and CH_Ar(*cyclo*Sal)_), 7.33 (1H, bs, CH_Ar(*cyclo*Sal)_), 7.55 (1H, d, ^3^*J* = 8.6 Hz, CH_Ar(*cyclo*Sal)_), 7.90 (2/10 of 1H, bs, CHO), 8.06 (8/10 of 1H, bs, CHO); ^13^C-NMR (125.8 MHz, CDCl_3_): 44.5 (NCH_2_), 55.6 (OCH_3_), 55.7 (OCH_3_), 64.4 (POCH_2_), 68.6 (d, ^2^*J*_C-P_ = 6.8 Hz, ArCH_2_OP), 73.2 (OCH_2_DMP), 98.8 (CH_Ar(DMB)_), 104.5 (CH_Ar(DMB)_), 114.8 (C_Ar(DMB)_), 119.7 (d, ^3^*J*_C-P_ = 9.4 Hz, *C*H*_Ar_*C_Ar_OP), 121.3 (d, ^3^*J*_C-P_ = 9.1 Hz, *C*_Ar_CH_2_OP), 123.1 (CH_Ar(*cyclo*Sal)_), 127.2 (CH_Ar(*cyclo*Sal)_), 133.1 (CH_Ar(DMB)_), 152.6 (d, ^2^*J*_C-P_ = 6.1 Hz, C_Ar_OP), 159.7 (*C*_Ar_OCH_3_), 162.3 (*C*_Ar_OCH_3_), 163.7 (CHO); ^31^P-NMR (162.0 MHz, CDCl_3_): −10.7; ^19^F-NMR (282.4 MHz, CDCl_3_): −63.2; HRMS (EI)^+^: *m*/*z* calculated for C_20_H_21_F_3_NO_8_PNa [M + Na]^+^ 514.0849, found 514.0876.
*N-((2,4-Dimethoxybenzyl)oxy)-N-(2-((6-methoxy-2-oxido-4H-benzo[d][1,3,2]dioxaphosphinin-2-yl)oxy)ethyl)formamide* (**17ag**). The general procedure D was applied to synthesize compound **17ag** from alcohol **9a** (96 mg, 0.38 mmol). The crude product was purified by automated flash chromatography (EtOAc/cyclohexane, 7:3 → EtOAc) to give **17ag** as a colorless oil (110 mg, 64%) and as a mixture of the two *Z* and *E* conformers in a 20:80 ratio. Rf = 0.28 (EtOAc/cyclohexane, 7:3); ^1^H-NMR (500 MHz, CDCl_3_): 3.54–3.89 (11H, m, NCH_2_ and OCH_3_), 4.34 (2H, bs, POCH_2_), 4.78 (8/10H of 2H, bs, OCH_2_DMP), 4.95 (2/10 of 2H, bs, OCH_2_DMP), 5.20–5.32 (2H, m, ArCH_2_OP), 6.41–6.43 (2H, m, CH_Ar(DMB)_), 6.51 (1H, s, CH_Ar(*cyclo*Sal)_), 6.78 (1H, d, ^3^*J* = 8.6 Hz, CH_Ar(*cyclo*Sal)_), 6.92 (1H, d, ^3^*J* = 9.2 Hz, CH_Ar(*cyclo*Sal)_), 7.10 (1H, m, CH_Ar(DMB)_), 7.84 (2/10H of 1H, bs, CHO), 8.04 (8/10 of 1H, bs, CHO);^13^C-NMR (125.8 MHz, CDCl_3_): 44.7 (d, ^3^*J*_CP_ = 6.5 Hz, NCH_2_), 55.6 (OCH_3_), 55.7 (OCH_3_), 55.9 (OCH_3_), 63.9 (POCH_2_), 69.1 (d, ^2^*J*_C-P_ = 7.1 Hz, ArCH_2_OP), 73.2 (OCH_2_DMP), 98.8 (CH_Ar(DMB)_), 104.5 (CH_Ar(DMB)_), 110.1 (CH_Ar(*cyclo*Sal)_), 114.9 (C_Ar(DMB)_), 115.2 (CH_Ar(*cyclo*Sal)_), 119.8 (d, ^3^*J*_C-P_ = 8.1 Hz, *C*H*_Ar_*C_Ar_OP), 121.3 (*C*_Ar_CH_2_OP), 133.1 (CH_Ar(DMB)_), 143.9 (d, ^2^*J*_C-P_ = 7.3 Hz, C_Ar_OP), 156.2 (*C*_Ar_OCH_3_), 159.7 (*C*_Ar_OCH_3_), 162.4 (*C*_Ar_OCH_3_), 163.8 (CHO); ^31^P-NMR (162.0 MHz, CDCl_3_): −9.4.; HRMS (EI)^+^: *m*/*z* calculated for C_20_H_24_NO_9_PNa [M + Na]^+^ 476.1081, found 476.1109.
*N-((2,4-Dimethoxylbenzyl)oxy)-N-(2-((2-oxido-4H-benzo[d][1,3,2]dioxaphosphinin-2-yl)oxy)ethyl)acetamide* (**17ba**). The general procedure D was applied to synthesize compound **17ba** from alcohol **9b** (190 mg, 0.71 mmol). The crude product was purified by flash chromatography (EtOAc/cyclohexane, 8:2) to give **17ba** as a colorless oil (227 mg, 73%) and as the sole *E* conformer. Rf = 0.47 (EtOAc/cyclohexane, 8:2); ^1^H-NMR (400 MHz, CDCl_3_): 1.99 (3H, s, CH_3_CO), 3.82 (3H, s, OCH_3_), 3.83 (3H, s, OCH_3_), 3.89–3.98 (2H, m, NCH_2_), 4.34–4.41 (2H, m, POCH_2_), 4.76 (2H, s, OCH_2_DMP), 5.23–5.38 (2H, m, ArCH_2_OP), 6.43–6.47 (2H, m, CH_Ar(DMB)_), 7.01 (2H, t, ^3^*J* = 8.6 Hz, CH_Ar(*cyclo*Sal)_), 7.10 (1H, t, ^3^*J* = 7.1 Hz, CH_Ar(*cyclo*Sal)_) 7.16 (1H, d, ^3^*J* = 8.1 Hz, CH_Ar(DMB)_), 7.27 (1H, m, CH_Ar(*cyclo*Sal)_); ^13^C-NMR (125.8 MHz, CDCl_3_): 20.2 (*C*H_3_CO), 46.1 (NCH_2_), 55.6 (OCH_3_), 55.7 (OCH_3_), 64.4 (d, ^2^*J*_CP_ = 5.5 Hz, POCH_2_), 68.9 (d, ^2^*J*_CP_ = 6.9 Hz, ArCH_2_OP), 71.6 (OCH_2_DMP), 98.7 (CH_Ar(DMB)_), 104.4 (CH_Ar(DMB)_), 115.1 (C_Ar(DMB)_), 119.0 (d, ^3^*J*_CP_ = 9.1 Hz, *CH_Ar_*C_Ar_OP), 120.7 (d, ^3^*J*_CP_ = 10.0 Hz, *C*_Ar_CH_2_OP), 124.4 (CH_Ar(*cyclo*Sal)_), 125.4 (CH_Ar(*cyclo*Sal)_), 129.9 (CH_Ar(*cyclo*Sal)_), 132.9 (CH_Ar(DMB)_), 150.3 (d, ^2^*J*_CP_ = 6.9 Hz, C_Ar_OP), 159.7 (*C*_Ar_OCH_3_), 162.3 (*C*_Ar_OCH_3_), 173.4 (CO); ^31^P-NMR (162.0 MHz, CDCl_3_): −9.90; MS (EI)^+^: *m*/*z* calculated for C_20_H_24_NO_8_PNa [M + Na]^+^ 460.11, found 460.11.
*N-((2,4-Dimethoxybenzyl)oxy)-N-(2-((6-methyl-2-oxido-4H-benzo[d][1,3,2]dioxaphosphinin-2-yl)oxy)ethyl)acetamide* (**17bb**). The general procedure D was applied to synthesize compound **17bb** from alcohol **9b** (200 mg, 0.78 mmol). The crude product was purified by flash chromatography (EtOAc/cyclohexane, 7:3 → EtOAc) to give **17bb** as a colorless oil (17 mg, 36%) and as the sole *E* conformer. Rf = 0.33 (EtOAc/petroleum ether, 7:3); ^1^H-NMR (400 MHz, CDCl_3_): 2.00 (3H, s, CH_3_CO), 2.29 (3H, s, CH_3_Ar), 3.82 (OCH_3_), 3.83 (OCH_3_), 3.87–3.97 (2H, m, NCH_2_), 4.30–4.42 (2H, m, POCH_2_), 4.76 (2H, s, OCH_2_DMP), 5.18–5.33 (2H, m, ArCH_2_OP), 6.43–6.46 (2H, m, CH_Ar(DMB)_), 6.81 (1H, bs, CH_Ar(*cyclo*Sal)_), 6.89 (1H, d, ^3^*J* = 8.3 Hz, CH_Ar(*cyclo*Sal)_), 7.05 (1H, d, ^3^*J* = 7.9 Hz, CH_Ar(*cyclo*Sal)_), 7.15 (1H, d, ^3^*J* = 7.9 Hz, CH_Ar(DMB)_); ^13^C-NMR (125.8 MHz, CDCl_3_): 20.1 (CH_3_CO), 20.8 (CH_3_Ar), 46.2 (NCH_2_), 55.5 (OCH_3_), 55.6 (OCH_3_), 64.4 (d, ^2^*J*_C-P_ = 5.6 Hz, POCH_2_), 69.0 (d, ^2^*J*_C-P_ = 7.0 Hz, ArCH_2_OP), 71.6 (OCH_2_DMP), 98.7 (CH_Ar(DMB)_), 104.3 (CH_Ar(DMB)_), 115.1 (C_Ar(DMB)_), 118.6 (d, ^3^*J*_C-P_ = 9.3 Hz, *C*H*_Ar_*C_Ar_OP), 120.3 (d, ^3^*J*_C-P_ = 9.5 Hz, *C*_Ar_CH_2_OP), 125.6 (CH_Ar(*cyclo*Sal)_), 130.3 (CH_Ar(*cyclo*Sal)_), 132.9 (CH_Ar(DMB)_), 134.1 (C_Ar_CH_3_), 148.1 (d, ^2^*J*_C-P_ = 7.1 Hz, C_Ar_OP), 159.6 (*C*_Ar_OCH_3_), 162.2 (*C*_Ar_OCH_3_), 173.5 (CO); ^31^P-NMR (162.0 MHz, CDCl_3_): −9.7; HRMS (EI)^+^: *m*/*z* calculated for C_21_H_26_NO_8_PNa [M + Na]^+^ 474.1288, found 474.1247.
*N-(2-((6-Chloro-2-oxido-4H-benzo[d][1,3,2]dioxaphosphinin-2-yl)oxy)ethyl)-N-((2,4-dimethoxybenzyl)oxy)acetamide* (**17bc**). The general procedure D was applied to synthesize compound **17bc** from alcohol **9b** (180 mg, 0.67 mmol). The crude product was purified by automated flash chromatography (EtOAc/cyclohexane, 8:2) to give **17bc** as a colorless oil (178 mg, 56%) and as the sole *E* conformer. Rf = 0.40 (EtOAc/cyclohexane, 8:2); ^1^H-NMR (400 MHz, CDCl_3_): 2.00 (3H, s, CH_3_CO), 3.83 (3H, s, OCH_3_), 3.84 (3H, s, OCH_3_), 3.91–3.96 (2H, m, NCH_2_), 4.35–4.42 (2H, m, POCH_2_), 4.76 (2H, s, OCH_2_DMP), 5.16–5.33 (2H, m, ArCH_2_OP), 6.44–6.47 (2H, m, CH_Ar(DMB)_), 6.93 (1H, d, ^3^*J* = 8.1 Hz CH_Ar(*cyclo*Sal)_), 7.00 (1H, d, ^4^*J* = 2.4 Hz, CH_Ar(*cyclo*Sal)_), 7.15 (1H, d, ^3^*J* = 8.0 Hz, CH_Ar(DMB)_) 7.22 (1H, d, ^3^*J* = 8.9 Hz, CH_Ar(*cyclo*Sal)_); ^13^C-NMR (125.8 MHz, CDCl_3_): 20.2 (*C*H_3_CO), 45.9 (NCH_2_), 55.6 (OCH_3_), 55.7 (OCH_3_), 64.7 (d, ^2^*J*_CP_ = 5.6 Hz, POCH_2_), 68.4 (d, ^2^*J*_CP_ = 7.2 Hz, ArCH_2_OP), 71.7 (OCH_2_DMP), 98.8 (CH_Ar(DMB)_), 104.4 (CH_Ar(DMB)_), 115.1 (C_Ar(DMB)_), 120.4 (d, ^3^*J*_CP_ = 9.1 Hz, *CH_Ar_*C_Ar_OP), 122.3 (d, ^3^*J*_CP_ = 9.9 Hz, *C*_Ar_CH_2_OP), 125.4 (CH_Ar(*cyclo*Sal)_),129.6 (C_Ar_Cl), 129.8 (CH_Ar(*cyclo*Sal)_), 132.9 (CH_Ar(DMB)_), 148.8 (d, ^2^*J*_CP_ = 7.0 Hz, C_Ar_OP), 159.7 (*C*_Ar_OCH_3_), 162.3 (*C*_Ar_OCH_3_), 173.4 (CO); ^31^P-NMR (162.0 MHz, CDCl_3_): −10.4; HRMS (EI)^+^: *m*/*z* calculated for C_20_H_23_ClNO_8_Pna [M + Na]^+^ 494.0683, found 494.0683.
*N-(2-((6,8-Dichloro-2-oxido-4H-benzo[d][1,3,2]dioxaphosphinin-2-yl)oxy)ethyl)-N-((2,4-dimethoxybenzyl)oxy)acetamide* (**17bd**). The general procedure D was applied to synthesize compound **17bd** from alcohol **9b** (214 mg, 0.79 mmol). The crude product was purified by automated flash chromatography (DCM/MeOH, 98:2) to give **17bd** as a colorless oil (15 mg, 12%) and as the sole *E* conformer. Rf = 0.32 (DCM/MeOH, 98:2); ^1^H-NMR (400 MHz, CDCl_3_): 2.02 (3H, s, CH_3_CO), 3.82 (3H, s, OCH_3_), 3.83 (3H, s, OCH_3_), 4.27–4.45 (2H, m, NCH_2_), 4.35–4.42 (2H, m, POCH_2_) 4.76 (2H, s, OCH_2_DMP), 5.17–5.32 (2H, m, ArCH_2_OP), 6.44–6.46 (2H, m, CH_Ar(DMB)_), 6.92 (1H, CH_Ar(*cyclo*Sal)_), 7.15 (1H, d, ^3^*J* = 8.3 Hz, CH_Ar(DMB)_) 7.35 (1H, d, ^3^*J* = 8.9 Hz, CH_Ar(*cyclo*Sal)_); ^13^C-NMR (125.8 MHz, CDCl_3_): 20.1 (*C*H_3_CO), 45.8 (NCH_2_), 55.6 (OCH_3_), 55.7 (OCH_3_), 65.1 (POCH_2_), 68.3 (d, ^2^*J*_CP_ = 7.0 Hz, ArCH_2_OP), 71.7 (OCH_2_DMP), 98.7 (CH_Ar(DMB)_), 104.4 (CH_Ar(DMB)_), 115.1 (C_Ar(DMB)_), 123.4 (d, ^3^*J*_CP_ = 9.5 Hz, Cl*C_Ar_*C_Ar_OP), 123.8 (CH_Ar(*cyclo*Sal)_) 124.9 (d, ^3^*J*_CP_ = 8.7 Hz,*C*_Ar_CH_2_OP), 129.5 (C_Ar_Cl), 130.2 (CH_Ar(*cyclo*Sal)_), 132.9 (CH_Ar(DMB)_), 145.1 (d, ^2^*J*_CP_ = 5.9 Hz, C_Ar_OP), 159.7 (*C*_Ar_OCH_3_), 162.4 (*C*_Ar_OCH_3_), 173.4 (CO); ^31^P-NMR (162.0 MHz, CDCl_3_): −10.7; MS (EI)^+^: *m*/*z* calculated for C_20_H_22_Cl_2_NO_8_PNa [M + Na]^+^ 528.04, found 528.03.
*N-(2-((6-Bromo-2-oxido-4H-benzo[d][1,3,2]dioxaphosphinin-2-yl)oxy)ethyl)-N-((2,4-dimethoxybenzyl)oxy)acetamide* (**17be**). The general procedure D was applied to synthesize compound **17be** from alcohol **9b** (60 mg, 0.22 mmol). The crude product was purified by automated flash chromatography (EtOAc/petroleum ether, 5:5) to give **17be** as a colorless oil (58 mg, 51%) and as the sole *E* conformer. Rf = 0.42 (EtOAc/petroleum ether, 7:3); ^1^H-NMR (500 MHz, CDCl_3_): 2.00 (3H, s, *C*H_3_CO), 3.82 (3H, s, OCH_3_), 3.83 (3H, s, OCH_3_) 3.87–3.99 (2H, m, NCH_2_), 4.33–4.43 (2H, m, POCH_2_), 4.75 (2H, s, OCH_2_DMP), 5.20 (1H, dd, ^2^*J* = 14.4 Hz, ^3^*J*_P-H_ = 19.1 Hz, ArCH_2_OP), 5.30 (1H, dd, ^2^*J* = 14.0 Hz, ^3^*J*_P-H_ = 8.0 Hz, ArCH_2_OP), 6.44–6.47 (2H, m, CH_Ar(DMB)_), 6.87 (1H, d, ^3^*J* = 8.6 Hz, CH_Ar(*cyclo*Sal)_), 7.14–7.17 (2H, m, CH_Ar(DMB)_ and CH_Ar(*cyclo*Sal)_), 7.37 (1H, d, ^3^*J* = 9.3 Hz, CH_Ar(*cyclo*Sal)_); ^13^C-NMR (125.8 MHz, CDCl_3_): 20.1 (*C*H_3_CO), 45.9 (NCH_2_), 55.6 (OCH_3_), 55.7 (OCH_3_), 64.7 (d, ^2^*J*_C-P_ = 5.7 Hz, POCH_2_), 68.3 (d, ^2^*J*_C-P_ = 7.0 Hz, ArCH_2_OP), 71.7 (OCH_2_DMP), 98.8 (CH_Ar(DMB)_), 104.4 (CH_Ar(DMB)_), 115.0 (C_Ar(DMB)_), 116.9 (C_Ar_Br), 120.7 (d, ^3^*J*_C-P_ = 9.0 Hz, *C*H*_Ar_*C_Ar_OP), 122.7 (d, ^3^*J*_C-P_ = 10.1 Hz, *C*_Ar_CH_2_OP), 128.3 (CH_Ar(*cyclo*Sal)_), 132.8 (CH_Ar(*cyclo*Sal)_), 132.9 (CH_Ar(DMB)_), 149.4 (d, ^2^*J*_C-P_ = 7.1 Hz, C_Ar_OP), 159.7 (*C*_Ar_OCH_3_), 162.3 (*C*_Ar_OCH_3_), 173.4 (CO); ^31^P-NMR (121.5 MHz, CDCl_3_): −10.3; HRMS (EI)^+^: *m*/*z* calculated for C_20_H_24_BrNO_8_P [M + H]^+^ 516.0417, found 516.0411.
*N-((2,4-Dimethoxybenzyl)oxy)-N-(2-((2-oxido-6-(trifluoromethyl)-4H-benzo[d][1,3,2]dioxaphosphinin-2-yl)oxy)ethyl)acetamide* (**17bf**). The general procedure D was applied to synthesize compound **17bf** from alcohol **9b** (98 mg, 0.36 mmol). The crude product was purified by automated flash chromatography EtOAc/cyclohexane, 7:3 → EtOAc) to give **17bf** as a colorless oil (73 mg, 52%) and as the sole *E* conformer. Rf = 0.42 (EtOAc/cyclohexane, 8:2); ^1^H-NMR (500 MHz, CDCl_3_): 1.97 (3H, s, *C*H_3_CO), 3.81 (6H, s, OCH_3_), 3.88–3.99 (2H, m, NCH_2_), 4.38–4.45 (2H, m, POCH_2_), 4.75 (2H, bs, OCH_2_DMP), 5.28 (1H, dd, ^2^*J* = 14.2 Hz, ^3^*J*_P-H_ = 18.7 Hz, ArCH_2_OP), 5.38 (1H, dd, ^2^*J* = 14.3 Hz, ^3^*J*_P-H_ = 7.5 Hz, ArCH_2_OP), 6.44–6.47 (2H, m, CH_Ar(DMB)_), 7.08 (1H, d, ^3^*J* = 8.4 Hz, CH_Ar(*cyclo*Sal)_), 7.15 (1H, d, ^3^*J* = 7.9 Hz, CH_Ar(DMB)_), 7.30 (1H, bs, CH_Ar(*cyclo*Sal)_), 7.54 (1H, d, ^3^*J* = 8.5 Hz, CH_Ar(*cyclo*Sal)_); ^13^C-NMR (125.8 MHz, CDCl_3_): 20.1 (*C*H_3_CO), 44.8 (NCH_2_), 55.6 (OCH_3_), 55.7 (OCH_3_), 64.8 (d, ^2^*J*_C-P_ = 5.6 Hz, POCH_2_), 68.5 (d, ^2^*J*_C-P_ = 6.9 Hz, ArCH_2_OP), 71.7 (OCH_2_DMP), 98.8 (CH_Ar(DMB)_), 104.4 (CH_Ar(DMB)_), 115.0 (C_Ar(DMB)_), 119.6 (d, ^3^*J*_C-P_ = 9.4 Hz, *C*H*_Ar_*C_Ar_OP), 121.4 (d, ^3^*J*_C-P_ = 9.9 Hz, *C*_Ar_CH_2_OP), 123.9 (q, ^3^*J*_C-F_ = 2.9 Hz, CHAr_(*cyclo*Sal)_), 125.8 (q, ^1^*J*_C-F_ = 273.4 Hz, C_Ar_*C*F_3_), 126.7 (q, ^2^*J*_C-F_ = 32.9 Hz, *C*_Ar_CF_3_), 127.2 (CH_Ar(*cyclo*Sal)_), 132.9 (CH_Ar(DMB)_), 152.7 (d, ^2^*J*_C-P_ = 6.9 Hz, C_Ar_OP), 159.7 (*C*_Ar_OCH_3_), 162.4 (*C*_Ar_OCH_3_), 173.3 (CO); ^31^P-NMR (121.5 MHz, CDCl_3_): −10.5; ^19^F-NMR (282.4 MHz, CDCl_3_): −63.2; HRMS (EI)^+^: *m*/*z* calculated for C_21_H_23_F_3_NO_8_PNa [M + Na]^+^ 528.1006, found 528.1001.
*N-((2,4-Dimethoxybenzyl)oxy)-N-(2-((6-methoxy-2-oxido-4H-benzo[d][1,3,2]dioxaphosphinin-2-yl)oxy)ethyl)acetamide* (**17bg**). The general procedure D was applied to synthesize compound **17bg** from alcohol **9b** (92 mg, 0.36 mmol). The crude product was purified by automated flash chromatography (EtOAc/cyclohexane, 7:3 → EtOAc) to give **17bg** as a colorless oil (94 mg, 56%) and as the sole *E* conformer. Rf = 0.27 (EtOAc/cyclohexane, 7:3); ^1^H-NMR (500 MHz, CDCl_3_): 2.00 (3H, s, *C*H_3_CO), 3.76 (3H, s, OCH_3_), 3.82 (3H, s, OCH_3_), 3.83 (3H, s, OCH_3_), 3.88–3.97 (2H, m, NCH_2_), 4.31–4.41 (2H, bs, POCH_2_), 4.75 (2H, bs, OCH_2_DMP), 5.19–5.33 (2H, m, ArCH_2_OP), 6.43–6.46 (2H, m, CH_Ar(DMB)_), 6.52 (1H, d, ^4^*J* = 2.9 Hz, CH_Ar(*cyclo*Sal)_), 6.79 (1H, dd, ^3^*J* = 8.9 Hz, ^4^*J* = 2.5 Hz, CH_Ar(*cyclo*Sal)_), 6.92 (1H, d, ^3^*J* = 9.1 Hz, CH_Ar(*cyclo*Sal)_), 7.16 (1H, d, ^3^*J* = 8.1 Hz, CH_Ar(DMB)_); ^13^C-NMR (125.8 MHz, CDCl_3_): 20.2 (*C*H_3_CO), 46.1 (NCH_2_), 55.6 (OCH_3_), 55.7 (OCH_3_), 55.9 (OCH_3_), 64.4 (d, ^2^*J*_C-P_ = 5.6 Hz, POCH_2_), 69.0 (d, ^2^*J*_C-P_ = 6.8 Hz, ArCH_2_OP), 71.7 (OCH_2_DMP), 98.7 (CH_Ar(DMB)_), 104.4 (CH_Ar(DMB)_), 110.1 (CH_Ar(*cyclo*Sal)_), 115.1 (CH_Ar(*cyclo*Sal)_), 119.8 (d, ^3^*J*_C-P_ = 8.8 Hz, *C*H*_Ar_*C_Ar_OP), 121.1 (d, ^3^*J*_C-P_ = 9.4 Hz, *C*_Ar_CH_2_OP), 132.9 (CH_Ar(DMB)_), 144.0 (d, ^2^*J*_C-P_ = 6.8 Hz, C_Ar_OP), 156.1 (*C*_Ar_OCH_3_), 159.7 (*C*_Ar_OCH_3_), 162.3 (*C*_Ar_OCH_3_), 173.5 (CO); ^31^P-NMR (121.5 MHz, CDCl_3_): −9.7; HRMS (EI)^+^: *m*/*z* calculated for C_21_H_26_NO_9_PNa [M + Na]^+^ 490.1237, found 490.1250.
*N-((2,4-Dimethoxybenzyl)oxy)-3-((2-oxido-4H-benzo[d][1,3,2]dioxaphosphinin-2-yl)oxy)propanamide* (**16aa**). The general procedure E was applied to synthesize compound **16aa** from alcohol **10a** (153 mg, 0.60 mmol). The crude product was purified by automated flash chromatography (EtOAc/petroleum ether, 8:2) to give **16aa** as a colorless oil (205 mg, 65%) and as a mixture of the two *Z* and *E* conformers in a 60:40 ratio. Rf = 0.38 (EtOAc/cyclohexane, 9:1); ^1^H-NMR (400 MHz, CDCl_3_): 2.48 (6/10H of 2H, bs, CH_2_CO), 2.83 (4/10H of 2H, bs, CH_2_CO), 3.81 (6H, s, OCH_3_), 4.40–4.49 (2H, m, POCH_2_), 4.77 (4/10 of 2H, bs, OCH_2_DMP), 4.85 (6/10 of 2H, bs, OCH_2_DMP), 5.34 (2H, m, ArCH_2_OP), 6.45 (2H, m, CH_Ar(DMP)_), 7.05 (2H, m, CH_Ar(*cyclo*Sal)_), 7.11 (1H, pseudo-t, ^3^*J* = 7.4 Hz, CH_Ar(*cyclo*Sal)_), 7.17 (1H, m, CH_Ar(DMP)_), 7.29 (1H, pseudo-t, ^3^*J* = 7.8 Hz, CH_Ar(*cyclo*Sal)_), 7.88 (4/10H of 1H, bs, NH), 8.35 (6/10H of 1H, bs, NH); ^13^C-NMR (125.8 MHz, CDCl_3_): 32.5 (d, ^3^*J*_C-P_ = 5.9 Hz, *C*H_2_CO), 34.6 (d, ^3^*J*_C-P_ = 5.1 Hz, *C*H_2_CO), 55.6 (OCH_3_), 55.7 (OCH_3_), 63.6 (POCH_2_), 64.4 (POCH_2_), 68.8 (ArCH_2_OP), 68.9 (ArCH_2_OP), 73.5 (OCH_2_DMP), 74.9 (OCH_2_DMP), 98.8 (CH_Ar(DMP)_), 104.2 (CH_Ar(DMP)_), 104.4 (CH_Ar(DMP)_), 114.9 (C_Ar(DMP)_), 115.9 (C_Ar(DMP)_), 118.9 (d, ^3^*J*_C-P_ = 9.0 Hz, *C*H_Ar_C_Ar_OP), 120.7 (*C*_Ar_CH_2_OP), 124.4 (CH_Ar(*cyclo*Sal)_) 124.6 (CH_Ar(*cyclo*Sal)_), 125.5 (CH_Ar(*cyclo*Sal)_), 129.9 (CH_Ar(DMP)_), 130.0 (CH_Ar(DMP)_), 132.9 (CH_Ar(*cyclo*Sal)_), 133.1 (CH_Ar(*cyclo*Sal)_), 150.2 (C_Ar_OP), 159.6 (*C*_Ar_OCH_3_), 159.7 (*C*_Ar_OCH_3_), 161.9 (*C*_Ar_OCH_3_), 162.3 (*C*_Ar_OCH_3_), 166.7 (CO), 173. (CO); ^31^P-NMR (121.5 MHz, CDCl_3_): −9.8, −9.9; MS (EI)^+^: *m*/*z* calculated for C_19_H_22_NO_8_Pna [M + Na]^+^ 446.10, found 446.10.
*N-((2,4-Dimethoxybenzyl)oxy)-3-((6-methyl-2-oxido-4H-benzo[d][1,3,2]dioxaphosphinin-2-yl)oxy)propanamide* (**16ab**). The general procedure E was applied to synthesize compound **16ab** from alcohol **10a** (200 mg, 0.78 mmol). The crude product was purified by automated flash chromatography (EtOAc/cyclohexane, 7:3 → EtOAc) to give **16ab** as a colorless oil (146 mg, 43%) and as a mixture of the two *Z* and *E* conformers in a 60:40 ratio. Rf = 0.36 (EtOAc/cyclohexane, 7:3); ^1^H-NMR (500 MHz, CDCl_3_): 2.26 (3H, s, CH_3_Ar), 2.42–2.51 (6/10 of 2H, m, CH_2_CO), 2.80–2.86 (4/10 of 2H, CH_2_CO), 3.79 (3H, s, OCH_3_), 3.80 (3H, s, OCH_3_), 4.39–4.45 (2H, m, POCH_2_), 4.75 (4/10 of 2H, s, OCH_2_DMP), 4.80–4.85 (6/10 of 2H, s, OCH_2_DMP), 5.21–5.31 (2H, m, ArCH_2_OP), 6.42–6.44 (2H, m, CH_Ar(DMP)_), 6.82 (1H, s, CH_Ar(*cyclo*Sal)_), 6.90 (1H, d, ^3^*J* = 8.3 Hz, CH_Ar(*cyclo*Sal)_), 7.05 (1H, d, 7.18 (^3^*J* = 8.5 Hz, CH_Ar(*cyclo*Sal)_) 7.13–7.17 (1H, m, CH_Ar(DMP)_), 7.85 (4/10H of 1H, bs, NH), 8.32 (6/10H of 1H, bs, NH); ^13^C-NMR (125.8 MHz, CDCl_3_): 20.9 (CH_3_Ar), 32.5 (*C*H_2_CO), 34.7 (*C*H_2_CO), 55.6 (OCH_3_), 55.8 (OCH_3_), 63.6 (POCH_2_), 64.3 (POCH_2_), 68.9 (d, ^2^*J*_C-P_ = 5.3 Hz ArCH_2_OP), 69.1 (d, ^2^*J*_C-P_ = 6.5 Hz ArCH_2_OP), 73.5 (OCH_2_DMP), 75.0 (OCH_2_DMP), 98.8 (CH_Ar(DMP)_), 104.3 (CH_Ar(DMP)_), 104.4 (CH_Ar(DMP)_), 116.0 (C_Ar(DMP)_), 118.6 (d, ^3^*J*_C-P_ = 8.8 Hz, *C*H_Ar_C_Ar_OP), 120.3 (*C*_Ar_CH_2_OP), 125.7 (CH_Ar(*cyclo*Sal)_), 130.4 (CH_Ar(*cyclo*Sal)_), 130.5 (CH_Ar(*cyclo*Sal)_), 132.9 (CH_Ar(DMP)_), 133.1 (CH_Ar(DMP)_), 134.3 (*C*_Ar_CH_3_), 148.0 (C_Ar_OP), 148.2 (C_Ar_OP), 159.6 (*C*_Ar_OCH_3_), 162.0 (*C*_Ar_OCH_3_), 162.3 (*C*_Ar_OCH_3_), 166.8 (*C*_Ar_OCH_3_), 171.4 (CO), 173.4 (CO); ^31^P-NMR (162.0 MHz, CDCl_3_): −9.3, −9.5; MS (EI)^+^: *m*/*z* calculated for C_20_H_24_NO_8_PNa [M + Na]^+^ 460.1132, found 460.1166.
*3-((6-Chloro-2-oxido-4H-benzo[d][1,3,2]dioxaphosphinin-2-yl)oxy)-N-((2,4-dimethoxybenzyl)oxy)propanamide* (**16ac**). The general procedure E was applied to synthesize compound **16ac** from alcohol **10a** (200 mg, 0.78 mmol). The crude product was purified by automated flash chromatography (EtOAc) to give **16ac** as a colorless oil (103 mg, 29%) and as a mixture of the two *Z* and *E* conformers in a 60:40 ratio. Rf = 0.34 (EtOAc); ^1^H-NMR (400 MHz, CDCl_3_): 2.49 (6/10 of 2H, m, CH_2_CO), 2.83 (4/10 of 2H, CH_2_CO), 3.82 (3H, s, OCH_3_), 3.83 (3H, s, OCH_3_), 4.42–4.51 (2H, m, POCH_2_), 4.78 (4/10 of 2H, s, OCH_2_DMP), 4.86 (6/10 of 2H, s, OCH_2_DMP), 5.22–5.38 (2H, m, ArCH_2_OP), 6.47 (2H, m, CH_Ar(DMP)_), 6.98 (1H, pseudo-d, ^3^*J* = 8.7 Hz, CH_Ar(*cyclo*Sal)_), 7.05 (1H, t, ^3^*J* = 2.1 Hz, CH_Ar(*cyclo*Sal)_), 7.18 (1H, m, CH_Ar(DMP)_), 7.24 (1H, bs, CH_Ar(*cyclo*Sal)_), 7.84 (4/10H of 1H, bs, NH), 8.29 (4/10H of 1H, bs, NH); ^13^C-NMR (125.8 MHz, CDCl_3_): 32.5 (*C*H_2_CO), 34.5 (*C*H_2_CO), 55.6 (OCH_3_), 55.8 (OCH_3_), 63.8 (POCH_2_), 64.6 (POCH_2_), 68.3 (ArCH_2_OP), 73.6 (OCH_2_DMP), 75.0 (OCH_2_DMP), 98.8 (CH_Ar(DMP)_), 104.3 (CH_Ar(DMP)_), 104.4 (CH_Ar(DMP)_), 114.9 (C_Ar(DMP)_), 115.9 (C_Ar(DMP)_), 120.3 (d, ^3^*J*_C-P_ = 9.4 Hz, *C*H_Ar_C_Ar_OP), 122.2 (*C*_Ar_CH_2_OP), 125.5 (CH_Ar(*cyclo*Sal)_), 130.3 (CH_Ar(*cyclo*Sal)_), 132.9 (CH_Ar(DMP)_), 148.7 (C_Ar_OP), 159.7 (*C*_Ar_OCH_3_), 166.6 (*C*_Ar_OCH_3_), 173.3 (CO); ^31^P-NMR (162.0 MHz, CDCl_3_): −10.3, −10.4; MS (EI)^+^: *m*/*z* calculated for C_19_H_21_ClNO_8_PNa [M + Na]^+^ 480.0586, found 480.0600.
*3-((6,8-Dichloro-2-oxido-4H-benzo[d][1,3,2]dioxaphosphinin-2-yl)oxy)-N-((2,4-dimethoxybenzyl)oxy)propanamide* (**16ad**). The general procedure E was applied to synthesize compound **16ad** from alcohol **10a** (250 mg, 0.98 mmol). The crude product was purified by flash chromatography (EtOAc/petroleum ether, 9:1) to give **16ad** as a colorless oil (49 mg, 10%) and as a mixture of the two *Z* and *E* conformers in a 60:40 ratio. Rf = 0.47 (EtOAc/cyclohexane, 9:1); ^1^H-NMR (500 MHz, CDCl_3_): 2.50 (6/10 of 2H, m, CH_2_CO), 2.83 (4/10 of 2H, CH_2_CO), 3.81–3.83 (6H, m, OCH_3_), 4.42–4.55 (2H, m, POCH_2_), 4.77 (4/10 of 2H, s, OCH_2_DMP), 4.84 (6/10 of 2H, s, OCH_2_DMP), 5.23–5.37 (2H, m, ArCH_2_OP), 6.44 (2H, pseudo-s, CH_Ar(DMP)_), 6.97 (1H, s, CH_Ar(*cyclo*Sal)_), 7.17 (1H, m, CH_Ar(*cyclo*Sal)_), 7.37 (1H, s, CH_Ar(DMP)_), 8.14 (4/10H of 1H, bs, NH), 8.64 (6/10H of 1H, bs, NH); ^13^C-NMR (125.8 MHz, CDCl_3_): 32.4 (*C*H_2_CO), 34.3 (*C*H_2_CO), 55.6 (OCH_3_), 55.7 (OCH_3_), 64.2 (POCH_2_), 65.0 (POCH_2_), 68.1 (ArCH_2_OP), 68.2 (ArCH_2_OP), 73.5 (OCH_2_DMP), 74.9 (OCH_2_DMP), 98.7 (CH_Ar(DMP)_), 104.3 (CH_Ar(DMP)_), 104.4 (CH_Ar(DMP)_), 114.9 (C_Ar(DMP)_), 115.9 (C_Ar(DMP)_), 123.5 (Cl*C*_Ar_C_Ar_OP), 123.9 (CH_Ar(*cyclo*Sal)_), 124.0 (CH_Ar(*cyclo*Sal)_), 124.8 (*C*_Ar_CH_2_OP), 129.5 (C_Ar_Cl), 129.8 (C_Ar_Cl), 130.3 (CH_Ar(*cyclo*Sal)_), 132.9 (CH_Ar(DMP)_), 133.1 (CH_Ar(DMP)_), 144.9 (C_Ar_OP), 145.1 (C_Ar_OP), 159.6 (*C*_Ar_OCH_3_), 159.7 (*C*_Ar_OCH_3_), 161.9 (*C*_Ar_OCH_3_), 162.3 (*C*_Ar_OCH_3_), 166.5 (CO), 173.2 (CO); ^31^P-NMR (162.0 MHz, CDCl_3_): −10.3, −10.4; MS (EI)^+^: *m*/*z* calculated for C_19_H_21_Cl_2_NO_8_PNa [M + Na]^+^ 492.04, found 492.04.
*3-((6-Bromo-2-oxido-4H-benzo[d][1,3,2]dioxaphosphinin-2-yl)oxy)-N-((2,4-dimethoxybenzyl)oxy)propanamide* (**16ae**). The general procedure E was applied to synthesize compound **16ae** from alcohol **10a** (139 mg, 0.54 mmol). The crude product was purified by flash chromatography (EtOAc/cyclohexane, 6:4 → EtOAc) to give **16ae** as a colorless oil (29 mg, 11%) and as a mixture of two *Z* and *E* conformers in a 60:40 ratio. Rf = 0.37 (EtOAc/cyclohexane, 7:3); ^1^H-NMR (500 MHz, CDCl_3_): 2.48 (6/10 of 2H, bs, CH_2_CO), 2.81 (4/10 of 2H, bs, CH_2_CO), 3.81 (6H, s, OCH_3_), 4.37–4.54 (2H, m, POCH_2_), 4.76 (4/10 of 2H, bs, OCH_2_DMP), 4.85 (6/10 of 2H, bs, OCH_2_DMP), 5.24 (1H, dd, ^2^*J* = 14.5 Hz, ^3^*J*_P-H_ = 18.0 Hz, ArCH_2_OP), 5.34 (1H, dd, ^2^*J* = 14.3 Hz, ^3^*J*_P-H_ = 9.5 Hz, ArCH_2_OP), 6.43–6.45 (2H, m, CH_Ar(DMP)_), 6.92 (1H, d, ^3^*J* = 8.7 Hz, CH_Ar(*cyclo*Sal)_), 7.11–7.19 (2H, m, CH_Ar(DMP)_ and CH_Ar(*cyclo*Sal)_), 7.39 (1H, d, ^3^*J* = 8.2 Hz, CH_Ar(*cyclo*Sal)_). 8.11 (4/10 of AH, bs, NH), 8.63 (6/10 of 1H, bs, NH); ^13^C-NMR (125.8 MHz, CDCl_3_): 32.4 (*C*H_2_CO), 34.4 (*C*H_2_CO), 55.6 (OCH_3_), 55.7 (OCH_3_), 63.8 (POCH_2_), 64.7 (POCH_2_), 68.1 (d, ^2^*J*_C-P_ = 6.7 Hz, ArCH_2_OP), 68.2 (d, ^2^*J*_C-P_ = 6.3 Hz, ArCH_2_OP), 73.5 (OCH_2_DMP), 74.6 (OCH_2_DMP), 98.8 (CH_Ar(DMP)_), 104.3 (CH_Ar(DMP)_), 104.4 (CH_Ar(DMP)_), 114.9 (C_Ar(DMP)_), 116.0 (C_Ar(DMP)_), 116.9 (C_Ar_Br), 117.1 (C_Ar_Br), 120.6 (d, ^3^*J*_C-P_ = 9.1 Hz, *C*H_Ar_C_Ar_OP), 122.5 (d, ^3^*J*_C-P_ = 9.5 Hz, *C*_Ar_CH_2_OP), 122.7 (d, ^3^*J*_C-P_ = 10.3 Hz, *C*_Ar_CH_2_OP), 128.3 (CH_Ar(*cyclo*Sal)_), 128.4 (CH_Ar(*cyclo*Sal)_), 132.9–133.1 (CH_Ar(*cyclo*Sal)_ and CH_Ar(DMP)_), 149.2 (d, ^2^*J*_C-P_ = 6.4 Hz, C_Ar_OP), 149.4 (d, ^2^*J*_C-P_ = 6.4 Hz, C_Ar_OP), 159.6 (*C*_Ar_OCH_3_), 159.7 (*C*_Ar_OCH_3_), 161.9 (*C*_Ar_OCH_3_), 162.3 (*C*_Ar_OCH_3_), 166.6 (CO), 173.2 (CO); ^31^P-NMR (121.5 MHz, CDCl_3_): −10.1, −10.2; HRMS (EI)^+^: *m*/*z* calculated for C_19_H_22_BrNO_8_PNa [M + Na]^+^ 502.0261, found 502.0331.
*N-((2,4-Dimethoxybenzyl)oxy)-3-((2-oxido-6-(trifluoromethyl)-4H-benzo[d][1,3,2]dioxaphosphinin-2-yl)oxy)propanamide* (**16af**). The general procedure E was applied to synthesize compound **16af** from alcohol **10a** (152 mg, 0.60 mmol). The crude product was purified by automated flash chromatography (EtOAc/cyclohexane, 3:7 → EtOAc) to give **16af** as a colorless oil (38 mg, 13%) and as a mixture of the two *Z* and *E* conformers in a 60:40 ratio. Rf = 0.33 (EtOAc/cyclohexane, 7:3); ^1^H-NMR (500 MHz, CDCl_3_): 2.47–2.49 (6/10 of 2H, m, CH_2_CO), 2.76–2.88 (4/10 of 2H, m, CH_2_CO), 3.80 (6H, s, OCH_3_), 4.42–4.54 (2H, m, POCH_2_), 4.76 (4/10 of 2H, bs, OCH_2_DMP), 4.84 (6/10 of 2H, s, OCH_2_DMP), 5.27–5.43 (2H, m, ArCH_2_OP), 6.24–6.44 (2H, m, CH_Ar(DMP)_), 7.13–7.16 (2H, m, CH_Ar(*cyclo*Sal)_ and CH_Ar(DMP)_), 7.33 (1H, s, CH_Ar(*cyclo*Sal)_), 7.55 (1H, d, ^3^*J* = 6.3 Hz, CH_Ar(*cyclo*Sal)_), 7.83 (4/10 of 1H, bs, NH), 8.28 (6/10 of 1H, bs, NH); ^13^C-NMR (125.8 MHz, CDCl_3_): 32.5 (*C*H_2_CO), 34.5 (*C*H_2_CO), 55.6 (OCH_3_), 55.8 (OCH_3_), 64.0 (POCH_2_), 64.7 (POCH_2_), 68.3–68.5 (ArCH_2_OP), 73.6 (OCH_2_DMP), 75.0 (OCH_2_DMP), 98.8 (CH_Ar(DMP)_), 104.3 (CH_Ar(DMP)_), 104.5 (CH_Ar(DMP)_), 114.9 (C_Ar(DMP)_), 115.2 (C_Ar(DMP)_), 119.6 (d, ^3^*J*_C-P_ = 9.5 Hz, *C*H_Ar_C_Ar_OP), 121.4 (*C*_Ar_CH_2_OP), 123.2 (CH_Ar(*cyclo*Sal)_), 127.4 (CH_Ar(*cyclo*Sal)_), 132.9 (CH_Ar(DMP)_) 133.1 (CH_Ar(DMP)_), 152.7 (C_Ar_OP), 159.7 (*C*_Ar_OCH_3_), 162.0 (*C*_Ar_OCH_3_), 162.4 (*C*_Ar_OCH_3_), 166.5 (*C*_Ar_OCH_3_), 171.4 (CO), 173.2 (CO); ^31^P-NMR (121.5 MHz, CDCl_3_): −10.4, −10.5; ^19^F-NMR (282.4 MHz, CDCl_3_): −63.2, −63.3; HRMS (EI)^+^: *m*/*z* calculated for C_20_H_22_NO_8_P [M + H]^+^ 492.1030, found 492.1011.
*N-((2,4-Dimethoxybenzyl)oxy)-3-((6-methoxy-2-oxido-4H-benzo[d][1,3,2]dioxaphosphinin-2-yl)oxy)propanamide* (**16ag**). The general procedure E was applied to synthesize compound **16ag** from alcohol **10a** (175 mg, 0.69 mmol). The crude product was purified by flash chromatography (EtOAc/cyclohexane, 3:7 → EtOAc) to give **16ag** as a colorless oil (49 mg, 18%) and as a mixture of two *Z* and *E* conformers in a 60:40 ratio. Rf = 0.46 (EtOAc); ^1^H-NMR (300 MHz, CDCl_3_): 2.48–2.49 (6/10 of 2H, m, CH_2_CO), 2.82–2.86 (4/10 of 2H, m, CH_2_CO), 3.75 (3H, s, OCH_3_), 3.81 (3H, s, OCH_3_), 3.82 (3H, s, OCH_3_), 4.40–4.47 (2H, m, POCH_2_), 4.77 (4/10 of 2H, bs, OCH_2_DMP), 4.85 (6/10 of 2H, s, OCH_2_DMP), 5.21–5.33 (2H, m, ArCH_2_OP), 6.43–6.46 (2H, m, CH_Ar(DMP)_), 6.55 (1H, d, ^4^*J* = 2.8 Hz CH_Ar(*cyclo*Sal)_), 6.80 (1H, dd, ^3^*J* = 9.1 Hz, ^4^*J* = 1.8 Hz, CH_Ar(DMP)_), 6.96 (1H, d, ^3^*J* = 8.9 Hz, CH_Ar(DMP)_), 7.15–7.20 (1H, m, CH_Ar(*cyclo*Sal)_), 7.91 (4/10 of 1H, bs, NH), 8.40 (6/10 of 1H, bs, NH); ^13^C-NMR (125.8 MHz, CDCl_3_): 32.8 (*C*H_2_CO), 34.7 (*C*H_2_CO), 55.6 (OCH_3_), 55.7 (OCH_3_), 55.9 (OCH_3_), 63.6 (POCH_2_), 64.4 (POCH_2_), 68.8–69.0 (ArCH_2_OP), 73.5 (OCH_2_DMP), 75.0 (OCH_2_DMP), 98.8 (CH_Ar(DMP)_), 104.3 (CH_Ar(DMP)_), 110.2 (CH_Ar(*cyclo*Sal)_), 115.3 (CH_Ar(*cyclo*Sal)_), 116.0 (C_Ar(DMP)_), 119.7 (d, ^3^*J*_C-P_ = 8.8 Hz, *C*H_Ar_C_Ar_OP), 121.3–121.5 (*C*_Ar_CH_2_OP), 132.9–133.1 (CH_Ar(DMP)_), 143.8–144.0 (C_Ar_OP), 156.1 (*C*_Ar_OCH_3_), 156.3 (*C*_Ar_OCH_3_), 159.6 (*C*_Ar_OCH_3_), 162.0 (*C*_Ar_OCH_3_), 162.3 (*C*_Ar_OCH_3_), 166.8 (CO), 171.1 (CO); ^31^P-NMR (121.5 MHz, CDCl_3_): −9.3, −9.5; HRMS (EI): *m*/*z* calculated for C_20_H_24_NO_9_PNa [M + Na]^+^ 476.1081, found 476.1102.
*N-((2,4-Dimethoxybenzyl)oxy)-N-methyl-3-((2-oxido-4H-benzo[d][1,3,2]dioxaphosphinin-2-yl)oxy)propanamide* (**16ba**). The general procedure D was applied to synthesize compound **16ba** from alcohol **10b** (153 mg, 0.57 mmol). The crude product was purified by flash chromatography (EtOAc/cyclohexane, 8:2) to give **16ba** as a colorless oil (148 mg, 76%) and as the sole *E* conformer. Rf = 0.25 (EtOAc/cyclohexane, 8:2); ^1^H-NMR (300 MHz, CDCl_3_): 2.79–2.92 (2H, m, CH_2_CO), 3.18 (3H, s, NCH_3_), 3.82 (3H, s, OCH_3_), 3.83 (3H, s, OCH_3_), 4.39–4.51 (2H, m, POCH_2_), 4.77 (2H, s, OCH_2_DMP), 5.27–5.42 (2H, m, ArCH_2_OP), 6.47 (2H, m, CH_Ar(DMP)_), 7.02–7.06 (2H, m, CH_Ar(*cyclo*Sal)_), 7.11 (1H, t, ^3^*J* = 7.5 Hz, CH_Ar(*cyclo*Sal)_), 7.17 (1H, d, ^3^*J* = 8.9 Hz, CH_Ar(DMP)_), 7.28 (1H, t, ^3^*J* = 7.6 Hz, CH_Ar(*cyclo*Sal)_); ^13^C-NMR (75.5 MHz, CDCl_3_): 32.9 (d, ^3^*J*_C-P_ = 7.0 Hz, *C*H_2_CO), 33.3 (NCH_3_), 55.6 (OCH_3_), 55.7 (OCH_3_), 64.4 (d, ^2^*J*_C-P_ = 5.3 Hz, POCH_2_), 66.7 (d, ^2^*J*_C-P_ = 6.8 Hz, ArCH_2_OP), 71.3 (OCH_2_DMP), 98.8 (CH_Ar(DMP)_), 104.4 (CH_Ar(DMP)_), 115.0 (C_Ar(DMP)_), 118.9 (d, ^3^*J*_C-P_ = 9.0 Hz, *C*H_Ar_C_Ar_OP), 120.9 (d, ^3^*J*_C-P_ = 9.8 Hz, *C*_Ar_CH_2_OP), 124.3 (CH_Ar(*cyclo*Sal)_), 125.3 ((CH_Ar(*cyclo*Sal)_), 129.7 (CH_Ar(*cyclo*Sal)_), 132.8 (CH_Ar(DMP)_), 150.4 (d, ^2^*J*_C-P_ = 6.9 Hz, C_Ar_OP), 159.7 (*C*_Ar_OCH_3_), 162.3 (*C*_Ar_OCH_3_), 171.2 (CO); ^31^P-NMR (121.5 MHz, CDCl_3_): −9.5; MS (EI)^+^: *m*/*z* calculated for C_20_H_24_NO_8_PNa [M + Na]^+^ 460.11, found 460.11.
*N-((2,4-Dimethoxybenzyl)oxy)-N-methyl-3-((6-methyl-2-oxido-4H-benzo[d][1,3,2]dioxaphosphinin-2-yl)oxy)propanamide* (**16bb**). The general procedure D was applied to synthesize compound **16bb** from alcohol **10b** (150 mg, 0.56 mmol). The crude product was purified by automated flash chromatography (EtOAc/cyclohexane, 5:5 → EtOAc) to give **16bb** as a colorless oil (190 mg, 75%) and as the sole *E* conformer. Rf = 0.47 (EtOAc/cyclohexane, 7:3); ^1^H-NMR (500 MHz, CDCl_3_): 2.30 (3H, s, CH_3_Ar), 2.79–2.92 (2H, m, CH_2_CO), 3.18 (3H, s, NCH_3_), 3.82 (3H, s, OCH_3_), 3.83 (3H, s, OCH_3_), 4.37–4.50 (2H, m, POCH_2_), 4.77 (2H, s, OCH_2_DMP), 5.23–5.379 (2H, m, ArCH_2_OP), 6.45–6.47 (2H, m, CH_Ar(DMP)_), 6.87 (1H, bs, CH_Ar(*cyclo*Sal)_), 6.91 (1H, d, ^3^*J* = 8.5 Hz, CH_Ar(DMP)_), 7.07 (1H, d, ^3^*J* = 8.0 Hz, CH_Ar(DMP)_), 7.18 (1H, d, ^3^*J* = 8.9 Hz, CH_Ar(*cyclo*Sal)_); ^13^C-NMR (125.8 MHz, CDCl_3_): 20.9 (CH_3_Ar), 33.0 (d, ^3^*J*_C-P_ = 7.1 Hz, *C*H_2_CO), 33.3 (NCH_3_), 55.6 (OCH_3_), 55.7 (OCH_3_), 64.4 (d, ^2^*J*_C-P_ = 5.4 Hz, POCH_2_), 68.8 (d, ^2^*J*_C-P_ = 6.5 Hz, ArCH_2_OP), 71.3 (OCH_2_DMP), 98.8 (CH_Ar(DMP)_), 104.4 (CH_Ar(DMP)_), 115.0 (C_Ar(DMP)_), 118.6 (d, ^3^*J*_C-P_ = 9.1 Hz, *C*H_Ar_C_Ar_OP), 120.5 (d, ^3^*J*_C-P_ = 9.6 Hz, *C*_Ar_CH_2_OP), 125.7 (CH_Ar(*cyclo*Sal)_), 130.3 (CH_Ar(*cyclo*Sal)_), 133.0 (CH_Ar(DMP)_), 134.0 (*C*_Ar_CH_3_), 148.3 (d, ^2^*J*_C-P_ = 6.4 Hz, C_Ar_OP), 159.7 (*C*_Ar_OCH_3_), 162.3 (*C*_Ar_OCH_3_), 171.2 (CO); ^31^P-NMR (121.5 MHz, CDCl_3_): −9.3; HRMS (EI)^+^: *m*/*z* calculated for C_21_H_27_NO_8_P [M + H]^+^ 452.1469, found 452.1508.
*3-((6-Chloro-2-oxido-4H-benzo[d][1,3,2]dioxaphosphinin-2-yl)oxy)-N-((2,4-dimethoxybenzyl)oxy)-N-methylpropanamide* (**16bc**). The general procedure D was applied to synthesize compound **16bc** from alcohol **10b** (195 mg, 0.72 mmol). The crude product was purified by flash chromatography (EtOAc/cyclohexane, 9:1) to give **16bc** as a colorless oil (292 mg, 86%) and as the sole *E* conformer. Rf = 0.51 (EtOAc/cyclohexane, 9:1); ^1^H-NMR (300 MHz, CDCl_3_): 2.78–2.90 (2H, m, CH_2_CO), 3.18 (3H, s, NCH_3_), 3.82 (3H, s, OCH_3_), 3.84 (3H, s, OCH_3_), 4.39–4.51 (2H, m, POCH_2_), 4.77 (2H, s, OCH_2_DMP), 5.21–5.39 (2H, m, ArCH_2_OP), 6.47 (2H, m, CH_Ar(DMP)_), 6.97 (1H, d, ^3^*J* = 8.9 Hz, CH_Ar(*cyclo*Sal)_), 7.05 (1H, t, ^3^*J* = 2.4 Hz, CH_Ar(*cyclo*Sal)_), 7.17 (1H, d, ^3^*J* = 8.6 Hz, CH_Ar(DMP)_), 7.24 (1H, bs, CH_Ar(*cyclo*Sal)_); ^13^C-NMR (75.5 MHz, CDCl_3_): 32.9 (d, ^3^*J*_C-P_ = 7.3 Hz, *C*H_2_CO), 33.3 (NCH_3_), 55.6 (OCH_3_), 55.7 (OCH_3_), 64.4 (d, ^2^*J*_C-P_ = 5.2 Hz, POCH_2_), 68.1 (d, ^2^*J*_C-P_ = 6.8 Hz, ArCH_2_OP), 71.3 (OCH_2_DMP), 98.8 (CH_Ar(DMP)_), 104.5 (CH_Ar(DMP)_), 115.0 (C_Ar(DMP)_), 118.9 (d, ^3^*J*_C-P_ = 9.0 Hz, *C*H_Ar_C_Ar_OP), 122.3 (d, ^3^*J*_C-P_ = 9.1 Hz, *C*_Ar_CH_2_OP), 125.4 (CH_Ar(*cyclo*Sal)_), 129.5 (C_Ar_Cl), 129.9 (CH_Ar(*cyclo*Sal)_), 133.0 (CH_Ar(DMP)_), 148.5 (d, ^2^*J*_C-P_ = 6.5 Hz, C_Ar_OP), 159.7 (*C*_Ar_OCH_3_), 162.3 (*C*_Ar_OCH_3_), 171.1 (CO); ^31^P-NMR (121.5 MHz, CDCl_3_): −10.0; MS (EI)^+^: *m*/*z* calculated for C_20_H_23_ClNO_8_PNa [M + Na]^+^ 494.0742, found 494.0626.
*3-((6,8-Dichloro-2-oxido-4H-benzo[d][1,3,2]dioxaphosphinin-2-yl)oxy)-N-((2,4-dimethoxybenzyl)oxy)-N-methylpropanamide* (**16bd**). The general procedure D was applied to synthesize compound **16bd** from alcohol **10b** (241 mg, 0.89 mmol). The crude product was purified by flash chromatography (EtOAc/cyclohexane, 8:2) to give **16bd** as a colorless oil (175 mg, 43%) and as the sole *E* conformer. Rf = 0.40 (EtOAc/petroleum ether, 8:2); ^1^H-NMR (400 MHz, CDCl_3_): 2.84–2.89 (2H, m, CH_2_CO), 3.19 (3H, s, NCH_3_), 3.82 (3H, s, OCH_3_), 3.85 (3H, s, OCH_3_), 4.41–4.62 (2H, m, POCH_2_), 4.78 (2H, s, OCH_2_DMP), 5.22–5.39 (2H, m, ArCH_2_OP), 6.47 (2H, m, CH_Ar(DMP)_), 6.97 (1H, d, ^3^*J* = 2.3 Hz, CH_Ar(*cyclo*Sal)_), 7.20 (1H, t, ^3^*J* = 8.9 Hz, CH_Ar(DMP)_), 7.38 (1H, bs, CH_Ar(*cyclo*Sal)_); ^13^C-NMR (125.8 MHz, CDCl_3_): 32.7 (d, ^3^*J*_C-P_ = 6.9 Hz, *C*H_2_CO), 33.1 (NCH_3_), 55.4 (OCH_3_), 55.5 (OCH_3_), 64.7 (d, ^2^*J*_C-P_ = 5.5 Hz, POCH_2_), 67.9 (d, ^2^*J*_C-P_ = 6.9 Hz, ArCH_2_OP), 71.2 (OCH_2_DMP), 98.6 (CH_Ar(DMP)_), 104.3 (CH_Ar(DMP)_), 114.7 (C_Ar(DMP)_), 123.4 (d, ^3^*J*_C-P_ = 9.5 Hz, Cl*C*_Ar_C_Ar_OP), 123.7 (CH_Ar(*cyclo*Sal)_), 124.7 (d, ^3^*J*_C-P_ = 8.8 Hz, *C*_Ar_CH_2_OP) 129.3 (C_Ar_Cl), 130.0 (CH_Ar(*cyclo*Sal)_), 132.8 (CH_Ar(DMP)_), 145.1 (d, ^2^*J*_C-P_ = 5.9 Hz, C_Ar_OP), 159.5 (*C*_Ar_OCH_3_), 162.2 (*C*_Ar_OCH_3_), 171.2 (CO); ^31^P-NMR (162.0 MHz, CDCl_3_): −10.5; MS (EI)^+^: *m*/*z* calculated for C_20_H_22_Cl_2_NO_8_PNa [M + Na]^+^ 528.04, found 528.04.
*3-((6-Bromo-2-oxido-4H-benzo[d][1,3,2]dioxaphosphinin-2-yl)oxy)-N-((2,4-dimethoxybenzyl)oxy)-N-methylpropanamide* (**16be**). The general procedure D was applied to synthesize compound **16be** from alcohol **10b** (150 mg, 0.56 mmol). The crude product was purified by automated flash chromatography (EtOAc/cyclohexane, 5:5 → EtOAc) to give **16be** as a colorless oil (215 mg, 74%) and as the sole *E* conformer. Rf = 0.47 (EtOAc/cyclohexane, 7:3); ^1^H-NMR (500 MHz, CDCl_3_): 2.76–2.91 (2H, m, CH_2_CO), 3.18 (3H, s, NCH_3_), 3.82 (3H, s, OCH_3_), 3.83 (3H, s, OCH_3_), 4.38–4.52 (2H, m, POCH_2_), 4.77 (2H, s, OCH_2_DMP), 5.24 (1H, dd, ^2^*J* = 14.2 Hz, ^3^*J*_P-H_ = 17.9 Hz, ArCH_2_OP), 5.36 (1H, dd, ^2^*J* = 14.1 Hz, ^3^*J*_P-H_ = 8.7 Hz, ArCH_2_OP), 6.45–6.47 (2H, m, CH_Ar(DMP)_), 6.91 (1H, d, ^3^*J* = 8.8 Hz, CH_Ar(*cyclo*Sal)_), 7.16–7.20 (2H, m, CH_Ar(DMP)_ and CH_Ar(*cyclo*Sal)_), 7. 38 (1H, d, ^3^*J* = 8.7 Hz, CH_Ar(*cyclo*Sal)_); ^13^C-NMR (125.8 MHz, CDCl_3_): 32.9 (d, ^3^*J*_C-P_ = 7.0 Hz, *C*H_2_CO), 33.3 (NCH_3_), 55.6 (OCH_3_), 55.7 (OCH_3_), 64.5 (d, ^2^*J*_C-P_ = 5.2 Hz, POCH_2_), 68.0 (d, ^2^*J*_C-P_ = 6.9 Hz, ArCH_2_OP), 71.3 (OCH_2_DMP), 98.8 (CH_Ar(DMP)_), 104.5 (CH_Ar(DMP)_), 114.9 (C_Ar(DMP)_), 116.8 (C_Ar_Br), 120.7 (d, ^3^*J*_C-P_ = 9.2 Hz, *C*H_Ar_C_Ar_OP), 122.8 (d, ^3^*J*_C-P_ = 9.8 Hz, *C*_Ar_CH_2_OP), 128.3 (CH_Ar(*cyclo*Sal)_), 132.8 (CH_Ar(*cyclo*Sal)_), 133.0 (CH_Ar(DMP)_), 149.5 (d, ^2^*J*_C-P_ = 6.6 Hz, C_Ar_OP), 159.7 (*C*_Ar_OCH_3_), 162.3 (*C*_Ar_OCH_3_), 171.1 (CO); ^31^P-NMR (162.0 MHz, CDCl_3_): −10.3; HRMS (EI)^+^: *m*/*z* calculated for C_20_H_23_BrNO_8_PNa [M + Na]^+^ 538.0237, found 538.0255.
*3-((6-((Difluoro-λ^3^-methyl)-λ^2^-fluoranyl)-2-oxido-4H-benzo[d][1,3,2]dioxaphosphinin-2-yl)oxy)-N-((2,4-dimethoxybenzyl)oxy)-N-methylpropanamide* (**16bf**). The general procedure D was applied to synthesize the compound **16bf** from alcohol **10b** (150 mg, 0.56 mmol). The crude product was purified by automated flash chromatography (EtOAc/cyclohexane, 5 5 → EtOAc) to give **16bf** as a colorless oil 149 mg, 53%) and as the sole *E* conformer. Rf = 0.44 (EtOAc/cyclohexane, 7:3); ^1^H-NMR (500 MHz, CDCl_3_): 2.78–2.91 (2H, m, CH_2_CO), 3.18 (3H, s, NCH_3_), 3.82 (3H, s, OCH_3_), 3.84 (3H, s, OCH_3_), 4.41–4.54 (2H, m, POCH_2_), 4.77 (2H, s, OCH_2_DMP), 5.33 (1H, dd, ^2^*J* = 14.4 Hz, ^3^*J*_P-H_ = 18.5 Hz, ArCH_2_OP), 5.45 (1H, dd, ^2^*J* = 14.3 Hz, ^3^*J*_P-H_ = 8.4 Hz, ArCH_2_OP), 6.45–6.47 (2H, m, CH_Ar(DMP)_), 7.14 (1H, d, ^3^*J* = 8.7 Hz CH_Ar(*cyclo*Sal)_), 7.17 (1H, d, ^3^*J* = 8.6 Hz, CH_Ar(DMP)_), 7.35 (1H, s, CH_Ar(*cyclo*Sal)_), 7.57 (1H, d, ^3^*J* = 8.4 Hz, CH_Ar(*cyclo*Sal)_); ^13^C-NMR (125.8 MHz, CDCl_3_): 32.9 (d, ^3^*J*_C-P_ = 7.0 Hz, *C*H_2_CO), 33.3 (NCH_3_), 55.6 (OCH_3_), 55.7 (OCH_3_), 64.7 (d, ^2^*J*_C-P_ = 5.3 Hz, POCH_2_), 68.3 (d, ^2^*J*_C-P_ = 6.7 Hz, ArCH_2_OP), 71.3 (OCH_2_DMP), 98.8 (CH_Ar(DMP)_), 104.7 (CH_Ar(DMP)_), 114.9 (C_Ar(DMP)_), 119.6 (d, ^3^*J*_C-P_ = 9.3 Hz, *C*H_Ar_C_Ar_OP), 121.5 (d, ^3^*J*_C-P_ = 9.7 Hz, *C*_Ar_CH_2_OP), 123.1 (q, ^3^*J*_C-F_ = 3.2 Hz, CH_Ar(*cyclo*Sal)_), 123.7 (q, ^1^*J*_C-F_ = 271.1 Hz, C_Ar_*C*F_3_), 126.8 (q, ^2^*J*_C-F_ = 33 Hz, *C*_Ar_CF_3_), 127.2 (q, ^3^*J*_C-F_ = 3.6 Hz, CH_Ar(*cyclo*Sal)_), 133.0 (CH_Ar(DMP)_), 152.9 (d, ^2^*J*_C-P_ = 6.4 Hz, C_Ar_OP), 159.7 (*C*_Ar_OCH_3_), 162.4 (*C*_Ar_OCH_3_), 171.1 (CO); ^31^P-NMR (121.5 MHz, CDCl_3_): −10.3; ^19^F-NMR (282.4 MHz, CDCl_3_): −63.2; HRMS (EI)^+^: *m*/*z* calculated for C_21_H_23_F_3_NO_8_PNa [M + Na]^+^ 528.1006, found 528.1005.
*N-((2,4-Dimethoxybenzyl)oxy)-3-((6-methoxy-2-oxido-4H-benzo[d][1,3,2]dioxaphosphinin-2-yl)oxy)-N-methylpropanamide* (**16bg**). The general procedure D was applied to synthesize compound **16bg** from alcohol **10b** (150 mg, 0.56 mmol). The crude product was purified by flash chromatography (EtOAc/petroleum ether, 5:5 → EtOAc) to give **16bg** as a colorless oil (160 mg, 61%) and as the sole *E* conformer. Rf = 0.5 (EtOAc/cyclohexane, 8:2); ^1^H-NMR (500 MHz, CDCl_3_): 2.79–2.92 (2H, m, CH_2_CO), 3.18 (8/10 of 3H, s, NCH_3_), 3.23 (2/10 of 3H, s, NCH_3_), 3.76 (3H, s, OCH_3_), 3.82 (3H, s, OCH_3_), 3.83 (3H, s, OCH_3_), 4.37–4.50 (2H, m, POCH_2_), 4.77 (8/10 of 2H, s, OCH_2_DMP), 4.80 (2/10 of 2H, s, OCH_2_DMP), 5.23–5.37 (2H, m, ArCH_2_OP), 6.45–6.47 (2H, m, CH_Ar(DMP)_), 6.55 (1H, d, ^4^*J* = 2.8 Hz CH_Ar(*cyclo*Sal)_), 6.80 (1H, dd, ^3^*J* = 8.8 Hz, ^4^*J* = 1.9 Hz, CH_Ar(*cyclo*Sal)_), 6.95 (1H, d, ^3^*J* = 8.9 Hz, CH_Ar(DMP)_), 7.18 (1H, d, ^3^*J* = 8.5 Hz, CH_Ar(*cyclo*Sal)_); ^13^C-NMR (125.8 MHz, CDCl_3_): 32.8 (d, ^3^*J*_C-P_ = 7.1 Hz, *C*H_2_CO), 33.1 (NCH_3_), 55.4 (OCH_3_), 55.5 (OCH_3_), 55.8 (OCH_3_), 64.4 (d, ^2^*J*_C-P_ = 5.4 Hz, POCH_2_), 68.6 (d, ^2^*J*_C-P_ = 6.7 Hz, ArCH_2_OP), 71.1 (OCH_2_DMP), 98.6 (CH_Ar(DMP)_), 104.3 (CH_Ar(DMP)_), 110.1 (CH_Ar(*cyclo*Sal)_), 114.8 (C_Ar(DMP)_), 114.9 (CH_Ar(*cyclo*Sal)_), 119.6 (d, ^3^*J*_C-P_ = 9.2 Hz, *C*H_Ar_C_Ar_OP), 121.4 (d, ^3^*J*_C-P_ = 9.6 Hz, *C*_Ar_CH_2_OP), 132.8 (CH_Ar(DMP)_), 143.9 (d, ^2^*J*_C-P_ = 6.7 Hz, C_Ar_OP), 155.9 (*C*_Ar_OCH_3_), 159.5 (*C*_Ar_OCH_3_), 162.1 (*C*_Ar_OCH_3_), 171.1 (CO); ^31^P-NMR (121.5 MHz, CDCl_3_): −9.3; HRMS (EI)^+^: *m*/*z* calculated for C_21_H_26_NO_9_PNa [M + Na]^+^ 490.1237, found 490.1277.
*N-((2,4-Dimethoxybenzyl)oxy)-N-(2-oxido-4H-benzo[d][1,3,2]dioxaphosphinin-2-yl)-3-((2-oxido-4H-benzo[d][1,3,2]dioxaphosphinin-2-yl)oxy)propanamide* (**18a**). When general procedure D was applied to synthesize compound 16aa from alcohol **10a** (200 mg, 0.78 mmol), a byproduct **18a** was obtained. The crude product was purified by flash chromatography (EtOAc/petroleum ether, 5:5 → EtOAc) to give **18a** as a colorless oil (90 mg, 20%) and as a mixture of two diastereoisomers. Rf = 0.38 (EtOAc/cyclohexane, 5:5); ^1^H-NMR (500 MHz, CDCl_3_): 2.88 (2H, t, ^3^*J* = 6.3 Hz, CH_2_CO), 3.78 (3H, s, OCH_3_), 3.82 (3H, s, OCH_3_), 4.37–4.51 (2H, m, POCH_2_), 4.90–4.97 (2H, m, OCH_2_DMP), 5.11–5.27 (2H, m, ArCH_2_OP), 5.33–5.38 (1H, m, ArCH_2_OP), 5.44–5.50 (1H, m, ArCH_2_OP) 6.42–6.44 (2H, m, CH_Ar(DMP)_), 6.88 (5/10 of 1H, d ^3^*J* = 8.4 Hz, CH_Ar(*cyclo*Sal)_), 6.90 (5/10 of 1H, d, ^3^*J* = 8.5 Hz, CH_Ar(*cyclo*Sal)_), 6.96 (1H, d, ^3^*J* = 7.1 Hz, CH_Ar(*cyclo*Sal)_), 7.03 (2H, t, ^3^*J* = 7.5 Hz, CH_Ar(*cyclo*Sal)_), 7.10 (2H, t, ^3^*J* = 7.5 Hz, CH_Ar(*cyclo*Sal)_), 7.17 (1H, dd, ^3^*J* = 8.9 Hz, ^4^*J*_P-H_ = 2.0 Hz, CH_Ar(DMP)_), 7.28 (2H, m, CH_Ar(*cyclo*Sal)_); ^13^C-NMR (125.8 MHz, CDCl_3_): 32.9 (d, ^3^*J*_C-P_ = 7.1 Hz, *C*H_2_CO), 55.6 (OCH_3_), 55.7 (OCH_3_), 63.7 (POCH_2_), 68.9 (d, ^2^*J*_C-P_ = 6.9 Hz, ArCH_2_OP), 69.3 (d, ^2^*J*_C-P_ = 7.1 Hz, ArCH_2_OP), 69.4 (d, ^2^*J*_C-P_ = 7.3 Hz, ArCH_2_OP), 71.8 (OCH_2_DMP), 98.5 (CH_Ar(DMP)_), 104.1 (CH_Ar(DMP)_), 117.6 (C_Ar(DMP)_), 118.9 (d, ^3^*J*_C-P_ = 8.5 Hz, *C*H_Ar_C_Ar_OP), 119.0 (d, ^3^*J*_C-P_ = 8.8 Hz, *C*H_Ar_C_Ar_OP), 120.5 (d, ^3^*J*_C-P_ = 10.2 Hz, *C*_Ar_CH_2_OP), 120.8 (d, ^3^*J*_C-P_ = 9.8 Hz, *C*_Ar_CH_2_OP), 124.4 (CH_Ar(*cyclo*Sal)_), 124.7 (CH_Ar(*cyclo*Sal)_), 125.3 (CH_Ar(*cyclo*Sal)_), 125.4 (CH_Ar(*cyclo*Sal)_), 129.9 (CH_Ar(*cyclo*Sal)_), 131.6 (CH_Ar(DMP)_), 143.0 (d, ^2^*J*_C-P_ = 9.5 Hz, C_Ar_OP), 143.1 (d, ^2^*J*_C-P_ = 9.7 Hz, C_Ar_OP), 150.1 (d, ^2^*J*_C-P_ = 7.2 Hz, C_Ar_OP), 150.3 (d, ^2^*J*_C-P_ = 7.6 Hz, C_Ar_OP), 159.0 (*C*_Ar_OCH_3_), 161.3 (*C*_Ar_OCH_3_), 171.4 (CO); ^31^P-NMR (121.5 MHz, CDCl_3_): −9.6, −9.7, −18.7; HRMS (EI)^+^: *m*/*z* calculated for C_26_H_27_NO_11_P_2_Na [M + Na]^+^ 614.0952, found 614.0951.
*N-((2,4-Dimethoxybenzyl)oxy)-N-(6-methyl-2-oxido-4H-benzo[d][1,3,2]dioxaphosphinin-2-yl)-3-((6-methyl-2-oxido-4H-benzo[d][1,3,2]dioxaphosphinin-2-yl)oxy)propanamide* (**18b**). When general procedure D was applied to synthesize compound **16ab** from alcohol **10a** (200 mg, 0.78 mmol), a byproduct **18b** was obtained. The crude product was purified by flash chromatography (EtOAc/petroleum ether, 5:5 → EtOAc) to give **18b** as a colorless oil 92 mg, 38%) and as a mixture of two diastereoisomers. Rf = 0.72 (EtOAc/petroleum ether, 7:3); ^1^H-NMR (500 MHz, CDCl_3_): 2.27 (6H, s, CH_3_Ar), 2.83 (2H, t, ^3^*J* = 6.2 Hz, CH_2_CO), 3.76 (3H, s, OCH_3_), 3.80 (3H, s, OCH_3_), 4.33–4.46 (2H, m, POCH_2_), 4.88–4.94 (2H, m, OCH_2_DMP), 5.05–5.21 (2H, m, ArCH_2_OP), 5.29 (1H, d, ^2^*J* = 13.9 Hz, ^3^*J*_C-P_ = 8.1 Hz, ArCH_2_OP), 5.38–5.44 (1H, m, ArCH_2_OP) 6.40–6.42 (2H, m, CH_Ar(DMP)_), 6.72–6.79 (3H, m, CH_Ar_), 6.88 (5/10 of 1H, d, ^3^*J* = 6.0 Hz, CH_Ar(*cyclo*Sal)_), 6.90 (5/10 of 1H, d, ^3^*J* = 6.1 Hz, CH_Ar(*cyclo*Sal)_), 7.00–7.05 (2H, m, CH_Ar_), 7.14 (5/10 of 1H, d, ^3^*J* = 7.9 Hz, CH_Ar_), 7.15 (5/10 of 1H, d, ^3^*J* = 7.9 Hz, CH_Ar_); ^13^C-NMR (125.8 MHz, CDCl_3_): 20.8 (CH_3_Ar), 20.9 (CH_3_Ar), 32.9 (d, ^3^*J*_C-P_ = 6.7 Hz, *C*H_2_CO), 55.6 (OCH_3_), 55.7 (OCH_3_), 63.6 (POCH_2_), 68.9 (d, ^2^*J*_C-P_ = 6.6 Hz, ArCH_2_OP), 69.4 (d, ^2^*J*_C-P_ = 7.3 Hz, ArCH_2_OP), 69.5 (d, ^2^*J*_C-P_ = 7.1 Hz, ArCH_2_OP), 71.8 (OCH_2_DMP), 98.5 (CH_Ar(DMP)_), 104.1 (CH_Ar(DMP)_), 117.6 (C_Ar(DMP)_), 118.6 (*C*H_Ar_C_Ar_OP), 120.0 (d, ^3^*J*_C-P_ = 10.0 Hz, *C*_Ar_CH_2_OP), 120.4 (d, ^3^*J*_C-P_ = 10.1 Hz, *C*_Ar_CH_2_OP), 125.5 (CH_Ar(*cyclo*Sal)_), 125.7 (CH_Ar(*cyclo*Sal)_), 130.4 (CH_Ar(*cyclo*Sal)_), 131.5 (CH_Ar(DMP)_), 134.0 (*C*_Ar_CH_3_), 134.4 (*C*_Ar_CH_3_), 143.1 (d, ^2^*J*_C-P_ = 9.2 Hz, C_Ar_OP), 143.2 (d, ^2^*J*_C-P_ = 9.7 Hz, C_Ar_OP), 147.9 (d, ^2^*J*_C-P_ = 7.2 Hz, C_Ar_OP), 148.2 (d, ^2^*J*_C-P_ = 5.7 Hz, C_Ar_OP), 158.9 (*C*_Ar_OCH_3_), 161.2 (*C*_Ar_OCH_3_), 171.4 (CO); ^31^P-NMR (121.5 MHz, CDCl_3_): −9.4, −9.5, −18.5; HRMS (EI)^+^: *m*/*z* calculated for C_28_H_31_NO_11_P_2_Na [M + Na]^+^ 642.1265, found 642.1335.
*N-(6-Chloro-2-oxido-4H-benzo[d][1,3,2]dioxaphosphinin-2-yl)-3-((6-chloro-2-oxido-4H-benzo[d][1,3,2]ioxaphosphinine-2-yl)oxy)-N-((2,4-dimethoxybenzyl)oxy)propanamide* (**18c**). When general procedure D was applied to synthesize compound **16ac** from alcohol **10a** (212 mg, 0.78 mmol), a byproduct 18c was obtained. The crude product was purified by flash chromatography (EtOAc/petroleum ether, 5:5 → EtOAc) to give **18c** as a colorless oil 3 (24 mg, 84%) and as a mixture of two diastereoisomers. Rf = 0.83 (EtOAc); ^1^H-NMR (500 MHz, CDCl_3_): 2.88 (2H, t, ^3^*J* = 6.3 Hz, CH_2_CO), 3.77 (3H, s, OCH_3_), 3.83 (3H, s, OCH_3_), 4.35–4.54 (2H, m, POCH_2_), 4.87–4.97 (2H, m, OCH_2_DMP), 5.03–5.23 (2H, m, ArCH_2_OP), 5.27–5.34 (1H, m, ArCH_2_OP), 5.38–5.46 (1H, m, ArCH_2_OP), 6.43–6.46 (2H, m, CH_Ar(DMP)_), 6.79 (5/10 of 1H, d, ^3^*J* = 6.1 Hz, CH_Ar(*cyclo*Sal)_), 6.82 (5/10 of 1H, d, ^3^*J* = 6.3 Hz, CH_Ar(*cyclo*Sal)_), 6.92–7.02 (3H, m, CH_Ar(*cyclo*Sal)_), 7.14 (1H, dd, ^3^*J* = 8.8 Hz, ^4^*J* = 2.4 Hz, CH_Ar(*cyclo*Sal)_), 7.19–7.22 (2H, m, CH_Ar_); ^13^C-NMR (125.8 MHz, CDCl_3_): 32.7 (d, ^3^*J*_C-P_ = 7.0 Hz, *C*H_2_CO), 55.4 (OCH_3_), 55.5 (OCH_3_), 63.7 (d, ^2^*J*_C-P_ = 4.1 Hz, POCH_2_), 63.7 (d, ^2^*J*_C-P_ = 4.6 Hz, POCH_2_), 68.0 (d, ^2^*J*_C-P_ = 6.6 Hz, ArCH_2_OP), 68.1 (d, ^2^*J*_C-P_ = 6.6 Hz, ArCH_2_OP), 68.6 (d, ^2^*J*_C-P_ = 7.1 Hz, ArCH_2_OP), 68.7 (d, ^2^*J*_C-P_ = 7.1 Hz, ArCH_2_OP), 71.8 (OCH_2_DMP), 98.4 (CH_Ar(DMP)_), 103.9 (CH_Ar(DMP)_), 117.0 (C_Ar(DMP)_), 120.1 (*C*H_Ar_C_Ar_OP), 120.2 (*C*H_Ar_C_Ar_OP), 121.7 (d, ^3^*J*_C-P_ = 9.1 Hz, *C*_Ar_CH_2_OP), 122.1 (d, ^3^*J*_C-P_ = 9.0 Hz, *C*_Ar_CH_2_OP), 125.0 (CH_Ar(*cyclo*Sal)_), 125.2 (CH_Ar(*cyclo*Sal)_), 129.4 (C_Ar_Cl), 129.5 (C_Ar_Cl), 129.7 (CH_Ar(*cyclo*Sal)_), 129.8 (CH_Ar(*cyclo*Sal)_), 131.5 (CH_Ar(DMP)_), 131.6 (CH_Ar(DMP)_), 142.6 (d, ^2^*J*_C-P_ = 9.5 Hz, C_Ar_OP), 142.7 (d, ^2^*J*_C-P_ = 10.1 Hz, C_Ar_OP), 148.3 (d, ^2^*J*_C-P_ = 7.8 Hz, C_Ar_OP), 148.2 (d, ^2^*J*_C-P_ = 6.0 Hz, C_Ar_OP), 158.9 (*C*_Ar_OCH_3_), 161.3 (*C*_Ar_OCH_3_); ^31^P-NMR (121.5 MHz, CDCl_3_): −10.1, −10.2, −19.4, −19.5; HRMS (EI)^+^: *m*/*z* calculated for C_26_H_25_Cl_2_NO_11_P_2_Na [M + Na]^+^ 682.0172, found 682.0168.
*N-(6-Bromo-2-oxido-4H-benzo[d][1,3,2]ioxaphosphinin-2-yl)-3-((6-bromo-2-oxido-4H-benzo[d][1,3,2]dioxaphosphinin-2-yl)oxy)-N-((2,4-dimethoxybenzyl)oxy)propanamide* (**18e**). When general procedure D was applied to synthesize compound **16ae** from alcohol 10a (200 mg, 0.78 mmol), a byproduct **18e** was obtained. The crude product was purified by flash chromatography (EtOAc/petroleum ether, 5:5 → EtOAc/petroleum ether, 7:3) to give **18e** as a colorless oil (432 mg, 74%) and as a mixture of two diastereoisomers. Rf = 0.34 (EtOAc/petroleum ether, 5:5); ^1^H-NMR (400 MHz, CDCl_3_): 2.87 (2H, t, ^3^*J* = 6.1 Hz, CH_2_CO), 3.79 (3H, s, OCH_3_), 3.83 (3H, s, OCH_3_), 4.37–4.51 (2H, m, POCH_2_), 4.88–4.96 (2H, m, OCH_2_DMP), 5.04–5.22 (2H, m, ArCH_2_OP), 5.28–5.33 (1H, m, ArCH_2_OP), 5.38–5.45 (1H, m, ArCH_2_OP), 6.43–6.46 (2H, m, CH_Ar(DMP)_), 6.73 (5/10 of 1H, d, ^3^*J* = 8.3 Hz, CH_Ar(*cyclo*Sal)_), 6.75 (5/10 of 1H, d, ^3^*J* = 8.1 Hz, CH_Ar(*cyclo*Sal)_), 6.90 (5/10 of 1H, d, ^3^*J* = 8.1 Hz, CH_Ar(*cyclo*Sal)_), 6.92 (5/10 of 1H, d, ^3^*J* = 8.3 Hz, CH_Ar(*cyclo*Sal)_) 7.08–7.18 (3H, m, CH_Ar(*cyclo*Sal)_ and CH_Ar(DMP)_), 7.34–7.41 (2H, m, CH_Ar_); ^13^C-NMR (125.8 MHz, CDCl_3_): 32.8 (d, ^3^*J*_C-P_ = 6.4 Hz, *C*H_2_CO), 55.6 (OCH_3_), 55.7 (OCH_3_), 63.8 (d, ^2^*J*_C-P_ = 4.7 Hz, POCH_2_), 63.9 (d, ^2^*J*_C-P_ = 4.2 Hz, POCH_2_), 68.1 (d, ^2^*J*_C-P_ = 6.7 Hz, ArCH_2_OP), 68.2 (d, ^2^*J*_C-P_ = 6.7 Hz, ArCH_2_OP), 68.7 (d, ^2^*J*_C-P_ = 7.3 Hz, ArCH_2_OP), 68.8 (d, ^2^*J*_C-P_ = 7.6 Hz, ArCH_2_OP), 71.9 (OCH_2_DMP), 98.6 (CH_Ar(DMP)_), 104.1 (CH_Ar(DMP)_), 116.9 (C_Ar(DMP)_), 117.0 (C_Ar(DMP)_), 117.2 (C_Ar_Br), 117.3 (C_Ar_Br), 120.6 (*C*H_Ar_C_Ar_OP), 120.7 (*C*H_Ar_C_Ar_OP), 122.3 (d, ^3^*J*_C-P_ = 9.2 Hz, *C*_Ar_CH_2_OP), 122.7 (d, ^3^*J*_C-P_ = 9.9 Hz, *C*_Ar_CH_2_OP), 128.1 (CH_Ar_), 128.2 (CH_Ar_), 128.3 (CH_Ar_), 131.7 (CH_Ar_), 131.8 (CH_Ar_), 132.8 (CH_Ar_), 142.8 (d, ^2^*J*_C-P_ = 9.7 Hz, C_Ar_OP), 142.9 (d, ^2^*J*_C-P_ = 9.7 Hz, C_Ar_OP), 149.1 (d, ^2^*J*_C-P_ = 7.2 Hz, C_Ar_OP), 149.4 (d, ^2^*J*_C-P_ = 6.7 Hz, C_Ar_OP), 149.5 (d, ^2^*J*_C-P_ = 6.7 Hz, C_Ar_OP), 159.1 (*C*_Ar_OCH_3_), 161.4 (*C*_Ar_OCH_3_); ^31^P-NMR (162.0 MHz, CDCl_3_): −10.4, −10.5, −19.7, −19.8; HRMS (EI)^+^: *m*/*z* calculated for C_26_H_26_Br_2_NO_11_P_2_ [M + H]^+^ 749.9324, found 749.9361.
*N-((2,4-Dimethoxybenzyl)oxy)-N-(2-oxido-6-(trifluoromethyl)-4H-benzo[d][1,3,2]dioxaphosphinin-2-yl)-3-((2-oxido-6-(trifluoromethyl)-4H-benzo[d][1,3,2]dioxaphosphinin-2-yl)oxy)propanamide* (**18f**). When general procedure D was applied to synthesize compound **16af** from alcohol **10a** (152 mg, 0.60 mmol), a byproduct **18f** was obtained. The crude product was purified by automated flash chromatography (EtOAc/petroleum ether, 5:5 → EtOAc/petroleum ether, 7:3) to give **18f** as a colorless oil (59 mg, 15%) and as a mixture of two diastereoisomers. Rf = 0.90 (EtOAc/petroleum ether, 7:3); ^1^H-NMR (300 MHz, CDCl_3_): 2.90 (2H, t, ^3^*J* = 6.2 Hz, CH_2_CO), 3.77 (3H, s, OCH_3_), 3.82 (3H, s, OCH_3_), 4.20–4.55 (2H, m, POCH_2_), 4.88–4.95 (2H, m, OCH_2_DMP), 5.13–5.31 (2H, m, ArCH_2_OP), 5.39 (1H, d, ^2^*J* = 14.5 Hz, ^3^*J* = 7.7 Hz, ArCH_2_OP), 5.47–5.54 (1H, m, ArCH_2_OP), 6.41–6.50 (2H, m, CH_Ar(DMP)_), 6.93–6.97 (1H, m, CH_Ar_), 7.13–7.16 (1H, m, CH_Ar_), 7.23 (1H, s, CH_Ar_), 7.28–7.35 (2H, m, CH_Ar_), 7.45–7.58 (2H, m, CH_Ar_); ^13^C-NMR (125.8 MHz, CDCl_3_): 32.9 (d, ^3^*J*_C-P_ = 8.0 Hz, *C*H_2_CO), 55.5 (OCH_3_), 55.7 (OCH_3_), 64.1 (d, ^2^*J*_C-P_ = 5.6 Hz, POCH_2_), 68.4 (d, ^2^*J*_C-P_ = 6.6 Hz, ArCH_2_OP), 69.0 (d, ^2^*J*_C-P_ = 7.1 Hz, ArCH_2_OP), 72.0 (OCH_2_DMP), 98.6 (CH_Ar(DMP)_), 104.1 (CH_Ar(DMP)_), 117.1 (C_Ar(DMP)_), 119.6 (d, ^3^*J*_C-P_ = 3.7 Hz, *C*H_Ar_C_Ar_OP), 119.7 (d, ^3^*J*_C-P_ = 3.9 Hz, *C*H_Ar_C_Ar_OP), 121.0 (d, ^3^*J*_C-P_ = 10.4 Hz, *C*_Ar_CH_2_OP), 121.4 (d, ^3^*J*_C-P_ = 10.4 Hz, *C*_Ar_CH_2_OP), 122.8 (CH_Ar_), 123.1 (CH_Ar_), 127. (CH_Ar_), 131.9 (CH_Ar(DMP)_), 159.1 (*C*_Ar_OCH_3_), 161.5 (*C*_Ar_OCH_3_); ^31^P-NMR (121.5 MHz, CDCl_3_): −10.4, −10.5, −19.7, −19.8; ^19^F-NMR (282.4 MHz, CDCl_3_): −63.15, −63.16, −63.20, −63.21; HRMS (EI)^+^: *m*/*z* calculated for C_28_H_25_F_6_NO_11_P_2_Na [M + Na]^+^ 750.0699, found 750.0650.
*N-((2,4-Dimethoxybenzyl)oxy)-N-(6-methoxy-2-oxido-4H-benzo[d][1,3,2]dioxaphosphinin-2-yl)-3-((6-methoxy-2-oxido-4H-benzo[d][1,3,2]dioxaphosphinin-2-yl)oxy)propanamide* (**18g**). When general procedure D was applied to synthesize compound **16ag** from alcohol **10a** (175 mg, 0.69 mmol), a byproduct of **18g** was obtained. The crude product was purified by automated flash chromatography (EtOAc/petroleum ether, 3:7 → EtOAc) to give **18g** as a colorless oil (45 mg, 12%) and as a mixture of two diastereoisomers. Rf = 0.60 (EtOAc/petroleum ether, 7:3); ^1^H-NMR (300 MHz, CDCl_3_): 2.86 (2H, t, ^3^*J* = 6.3 Hz, CH_2_CO), 3.75 (6H, s, OCH_3_), 3.78 (3H, s, OCH_3_), 3.81 (3H, s, OCH_3_), 4.32–4.49 (2H, m, POCH_2_), 4.89–4.98 (2H, m, OCH_2_DMP), 5.05–5.20 (2H, m, ArCH_2_OP), 5.24–3.34 (1H, m, ArCH_2_OP), 5.38–5.46 (1H, m, ArCH_2_OP), 6.41–6.45 (3H, m, CH_Ar(DMP)_ and CH_Ar_), 6.52 (5/10 of 1H, s, CH_Ar_), 6.53 (5/10 of 1H, s, CH_Ar_), 6.74–6.84 (3H, m, CH_Ar_), 6.93–6.98 (1H, m, CH_Ar_), 7.16 (1H, dd, ^3^*J* = 8.8 Hz, ^4^*J* = 1.5 Hz, CH_Ar_); ^13^C-NMR (125.8 MHz, CDCl_3_): 32.9 (d, ^3^*J*_C-P_ = 6.9 Hz, *C*H_2_CO), 55.6 (OCH_3_), 55.7 (OCH_3_), 55.9 (OCH_3_), 63.6 (POCH_2_), 63.7 (POCH_2_), 68.8 (d, ^2^*J*_C-P_ = 6.6 Hz, ArCH_2_OP), 69.4 (d, ^2^*J*_C-P_ = 6.8 Hz, ArCH_2_OP), 69.5 (d, ^2^*J*_C-P_ = 6.8 Hz, ArCH_2_OP), 71.8 (OCH_2_DMP), 98.5 (CH_Ar(DMP)_), 104.1 (CH_Ar(DMP)_), 110.1 (CH_Ar_), 110.2 (CH_Ar_), 115.0 (CH_Ar_), 115.1 (CH_Ar_), 117.6 (C_Ar(DMP)_), 119.7 (*C*H_Ar_C_Ar_OP), 119.8 (*C*H_Ar_C_Ar_OP), 121.1 (d, ^3^*J*_C-P_ = 9.4 Hz, *C*_Ar_CH_2_OP), 121.4 (d, ^3^*J*_C-P_ = 9.4 Hz, *C*_Ar_CH_2_OP), 131.5 (CH_Ar(DMP)_), 143.1 (d, ^2^*J*_C-P_ = 9.6 Hz, C_Ar_OP), 143.2 (d, ^2^*J*_C-P_ = 9.6 Hz, C_Ar_OP), 143.7 (d, ^2^*J*_C-P_ = 7.3 Hz, C_Ar_OP), 144.0 (d, ^2^*J*_C-P_ = 6.7 Hz, C_Ar_OP), 156.1 (*C*_Ar_OCH_3_), 156.3 (*C*_Ar_OCH_3_), 158.9 (*C*_Ar_OCH_3_), 161.2 (*C*_Ar_OCH_3_), 171.4 (CO); ^31^P-NMR (121.5 MHz, CDCl_3_): −9.4, −9.5, −18.5; HRMS (EI)^+^: *m*/*z* calculated for C_28_H_31_NO_13_P_2_Na [M + Na]^+^ 674.1163, found 674.1134.
*N-Hydroxy-N-(2-((2-oxido-4H-benzo[d][1,3,2]dioxaphosphinin-2-yl)oxy)ethyl)formamide* (**7aa**). The general procedure F was applied to synthesize compound **7aa** from *cyclo*Sal **17aa** (93 mg, 0.22 mmol) and 2% TFA in DCM. The product **7aa** was obtained without purification as an orange oil (48 mg, 80%) and as a mixture of the three *Z*, *E*, and another conformer in a 20:20:60 ratio, respectively. Rf = 0.33 (EtOAc); ^1^H-NMR (500 MHz, CD_3_OD): 3.57–3.61 (2/10 of 2H, m, NCH_2_), 3.76–3.80 (6/10 of 2H, m, NCH_2_), 3.85–3.89 (2/10 of 2H, m, NCH_2_), 4.35–4.42 (8/10 of 2H, m, POCH_2_), 4.48–4.59 (2/10H of 2H, bs, POCH_2_), 5.39–5.56 (2H, m, ArCH_2_OP), 7.11 (1H, m, CH_Ar(*cyclo*Sal)_), 7.22 (2H, m, CH_Ar(*cyclo*Sal_), 7.38 (1H, m, CH_Ar(*cyclo*Sal)_), 7.86 (6/10 of 1H, bs, CHO), 7.91 (2/10 of 1H, bs, CHO), 8.19 (2/10 of 1H, bs, CHO); ^13^C-NMR (125.8 MHz, CD_3_OD): 47.8 (d, ^3^*J*_CP_ = 6.9 Hz, NCH_2_), 51.6 (d, ^3^*J*_CP_ = 7.2 Hz, NCH_2_), 52.0 (d, ^3^*J*_CP_ = 8.1 Hz, NCH_2_), 62.4 (d, ^2^*J*_CP_ = 4.8 Hz, POCH_2_), 64.9 (d, ^2^*J*_CP_ = 5.6 Hz, POCH_2_), 65.2 (d, ^2^*J*_CP_ = 5.6 Hz, POCH_2_), 70.3 (ArCH_2_OP), 70.4 (ArCH_2_OP), 70.5 (ArCH_2_OP), 119.6 (*CH_Ar_*C_Ar_OP), 119.7 (*CH_Ar_*C_Ar_OP), 119.8 (*CH_Ar_*C_Ar_OP), 122.3 (*C*_Ar_CH_2_OP), 122.4 (*C*_Ar_CH_2_OP), 122.5 (*C*_Ar_CH_2_OP), 125.9 (CH_Ar_), 126.1 (CH_Ar_), 126.2 (CH_Ar_), 126.9 (CH_Ar_), 127.1 (CH_Ar_), 127.2 (CH_Ar_), 131.1 (CH_Ar_), 131.2 (CH_Ar_), 131.4 (CH_Ar_), 151.3 (d, ^2^*J*_CP_ = 6.8 Hz, C_Ar_OP), 166.3 (CHO), 164.9 (CHO); ^31^P-NMR (121.5 MHz, CD_3_OD): −10.4; −10.5; −10.6; HRMS (EI)^+^: *m*/*z* calculated for C_10_H_12_NO_6_PNa [M + Na]^+^ 296.0294, found 296.0304.
*N-(2-((6-Chloro-2-oxido-4H-benzo[d][1,3,2]dioxaphosphinin-2-yl)oxy)ethyl)-N-hydroxyformamide* (**7ac**). The general procedure F was applied to synthesize compound **7ac** from *cyclo*Sal **17ac** (77 mg, 0.17 mmol) and 2% TFA in DCM. The product **7ac** was obtained without purification as an orange oil (38 mg, 73%) and as a mixture of the three *Z*, *E* and another conformer in a 30:40:30 ratio, respectively. Rf = 0.33 (EtOAc); ^1^H-NMR (400 MHz, CDCl_3_): 3.58 (3/10 of 2H, bs, NCH_2_), 3.81 (3/10 of 2H, bs, NCH_2_), 3.91 (4/10 of 2H, m, NCH_2_), 4.41–4.50 (8/10 of 2H, m, POCH_2_), 4.58–4.63 (2/10 of 2H, m, POCH_2_), 5.30–5.42 (2H, m, ArCH_2_OP), 7.03 (1H, m, CH_Ar_), 7.10 (1H, d, ^4^*J* = 2.2 Hz, CH_Ar_), 7.31 (1H, d, ^3^*J* = 8.6 Hz, CH_Ar_), 7.93 (3/10 of 1H, bs, CHO), 8.07 (1/10 of 1H, bs, CHO), 8.54 (4/10 of 1H, bs, CHO); ^13^C-NMR (125.8 MHz CDCl_3_): 46.4 (NCH_2_), 63.2 (d, ^2^*J* = 6.0 Hz, POCH_2_), 64.2 (d, ^2^*J* = 6.6 Hz, POCH_2_), 68.6 (d, ^2^*J*_CP_ = 6.9 Hz, ArCH_2_OP), 68.7 (d, ^2^*J*_CP_ = 6.9 Hz, ArCH_2_OP), 69.0 (d, ^2^*J*_CP_ = 6.9 Hz, ArCH_2_OP), 120.5 (d, ^3^*J*_CP_ = 9.2 Hz, *C*H*_Ar_*C_Ar_OP), 122.0 (d, ^3^*J*_CP_ = 10.1 Hz, *C*_Ar_CH_2_OP), 125.4 (d, ^4^*J* = 14.2 Hz CH_Ar(*cyclo*Sal)_), 130.4 (CH_Ar(*cyclo*Sal)_), 148.5 (C_Ar_OP), 164.8 (CHO); ^31^P-NMR (162.0 MHz, CDCl_3_): −6.71, −9.07, −9.63; HRMS (EI)^+^: *m*/*z* calculated for C_10_H_12_ClNO_6_PNa [M + Na]^+^ 308.0043, found 308.0085.
*N-Hydroxy-N-(2-((2-oxido-4H-benzo[d][1,3,2]dioxaphosphinin-2-yl)oxy)ethyl)acetamide* (**7ba**). The general procedure F was applied to synthesize compound **7ba** from *cyclo*Sal **17ba** (73 mg, 0.17 mmol) and 2% TFA in DCM. The product **7ba** was obtained without purification as an orange oil (41 mg, 84%). Rf = 0.35 (EtOAc); ^1^H-NMR (500 MHz, CDCl_3_): 1.99 (3H, s, CH_3_CO), 3.83–3.94 (2H, m, CH_2_N), 4.35–4.40 (2H, m, POCH_2_), 5.39–5.51 (2H, m, ArCH_2_OP), 7.10 (1H, m, CH_Ar(*cyclo*Sal)_), 7.22 (2H, m, CH_Ar(*cyclo*Sal)_), 7.37 (1H, t, ^3^*J* = 7.6 Hz, CH_Ar(*cyclo*Sal)_); ^13^C-NMR (125.8 MHz, CDCl_3_): 20.3 (CH_3_CO), 48.9 (d, ^3^*J* = 6.9 Hz, NCH_2_), 65.6 (d, ^2^*J* = 6.9 Hz, POCH_2_), 70.4 (d, ^2^*J*_CP_ = 6.9 Hz, ArCH_2_OP), 119.7 (d, ^3^*J*_CP_ = 9.1 Hz, *CH_Ar_*C_Ar_OP), 122.4 (d, ^3^*J*_CP_ = 9.9 Hz, *C*_Ar_CH_2_OP), 124.2 (CH_Ar_), 125.9 (CH_Ar_), 126.6 (CH_Ar_), 127.0 (CH_Ar_), 130.2 (CH_Ar_), 131.1 (CH_Ar_), 151.3 (d, ^2^*J*_CP_ = 6.8 Hz, C_Ar_OP), 172.3 (CO), 174.7 (CO); ^31^P-NMR (162.0 MHz, CDCl_3_): −8.9, −10.5; HRMS (EI)^+^: *m*/*z* calculated for C_11_H_14_NO_6_PNa [M + Na]^+^ 310.0451, found 310.0441.
*N-(2-((6-Chloro-2-oxido-4H-benzo[d][1,3,2]dioxaphosphinin-2-yl)oxy)ethyl)-N-hydroxyacetamide* (**7bc**). The general procedure F was applied to synthesize compound **7bc** from *cyclo*Sal **17bc** (71 mg, 0.15 mmol) 2% TFA in DCM. The product **200c** was obtained without purification as an orange oil (33 mg, 68%). Rf = 0.43 (EtOAc); ^1^H-NMR (400 MHz, CDCl_3_): 2.12–2.28 (3H, s, CH_3_CO), 3.58 (3/10 of 2H, bs, NCH_2_), 3.93–4.01 (2H, m, NCH_2_), 4.41–4.48 (2H, m, POCH_2_), 5.30–5.34 (2H, m, ArCH_2_OP), 7.03 (1H, d, ^3^*J* = 8.7 Hz CH_Ar_), 7.10 (1H, d, ^4^*J* = 2.2 Hz, CH_Ar_), 7.31 (1H, d, ^3^*J* = 8.1 Hz, CH_Ar_); ^13^C-NMR (125.8 MHz, CDCl_3_): 20.6 (CH_3_CO), 20.7 (CH_3_CO), 47.2 (NCH_2_), 65.2 (d, ^2^*J* = 6.9 Hz, POCH_2_), 68.7 (d, ^2^*J*_CP_ = 6.9 Hz, ArCH_2_OP), 120.2 (d, ^3^*J*_CP_ = 8.0 Hz, *C*H*_Ar_*C_Ar_OP), 120.5 (d, ^3^*J*_CP_ = 8.2 Hz, *C*H*_Ar_*C_Ar_OP), 122.7 (*C*_Ar_CH_2_OP), 125.4 (CH_Ar(*cyclo*Sal)_), 125.5 (CH_Ar(*cyclo*Sal)_), 128.7 (C_Ar_Cl), 129.5 (CH_Ar(*cyclo*Sal)_), 130.4 (CH_Ar(*cyclo*Sal)_);^31^P-NMR (162.0 MHz, CDCl_3_): −6.71; HRMS (EI)^+^: *m*/*z* calculated for C_11_H_14_ClNO_6_PNa [M + Na]^+^ 322.0242, found 322.0202.
*N-Hydroxy-3-((2-oxido-4H-benzo[d][1,3,2]dioxaphosphinin-2-yl)oxy)propanamide* (**8aa**). The general procedure F was applied to synthesize compound **8aa** from *cyclo*Sal **16aa** (80 mg, 0.19 mmol) and 3% TFA in DCM. The crude product was purified by preparative TLC (MeOH/EtOAc, 5:95 *v*/*v*) to give **8aa** as a colorless oil (31 mg, 60%) and as a sole *Z* conformer. Rf = 0.39 (EtOAc/methanol, 95:5);^1^H-NMR (400 MHz, CD_3_OD): 2.49 (2H, m, *C*H_2_CO), 4.37–4.51 (2H, m, POCH_2_), 5.38–5.48 (ArCH_2_OP), 7.10 (1H, pseudo-d, ^3^*J* = 7.9 Hz, CH_Ar(*cyclo*Sal)_), 7.17–7.23 (2H, m, CH_Ar(*cyclo*Sal)_), 7.37 (1H, pseudo-t, ^3^*J* = 7.6 Hz, CH_Ar_); ^13^C-NMR (125.8 MHz, CD_3_OD): 34.8 (d, ^3^*J*_C-P_ = 7.5 Hz, *C*H_2_CO), 65.9 (d, ^2^*J*_C-P_ = 5.3 Hz, POCH_2_), 70.2 (d, ^2^*J*_C-P_ = 6.9 Hz, ArCH_2_OP), 119.5 (d, ^3^*J*_C-P_ = 9.0 Hz, *C*H_Ar_C_Ar_OP), 122.4 (d, ^3^*J*_C-P_ = 9.8 Hz, *C*_Ar_CH_2_OP), 125.9 (CH_Ar(*cyclo*Sal)_), 127.1 (CH_Ar(*cyclo*Sal)_), 131.2 (CH_Ar(*cyclo*Sal)_), 151.3 (d, ^2^*J*_C-P_ = 6.8 Hz, C_Ar_OP), 169.0 (CO); ^31^P-NMR (162 MHz, CD_3_OD): −9.3; HRMS (EI)^+^: *m*/*z* calculated for C_10_H_12_NO_6_PNa [M + Na]^+^ 296.0294, found 296.0266.
*3-((6-Chloro-2-oxido-4H-benzo[d][1,3,2]dioxaphosphinin-2-yl)oxy)-N-hydroxypropanamide* (**8ac**). The general procedure F was applied to synthesize compound **8ac** from *cyclo*Sal **16ac** (93 mg, 0.20 mmol) and 2% TFA in DCM. The product **201c** was obtained without purification as a colorless oil (50 mg, 81%) and as a mixture of two *Z* and *E* conformers in a 60:40 ratio. Rf = 0.18 (EtOAc); ^1^H-NMR (500 MHz, CDCl_3_): 2.61 (6/10 of 2H, bs, CH_2_CO), 2.93 (4/10 of 2H, bs, CH_2_CO), 4.42–4.47 (2H, m, POCH_2_), 5.24–5.35 (2H, m, ArCH_2_OP), 6.97 (1H, pseudo-d, ^3^*J* = 8.4 Hz, CH_Ar(*cyclo*Sal)_), 7.05 (1H, pseudo-s, CH_Ar(*cyclo*Sal)_), 7.24 (1H, pseudo-s, CH_Ar(*cyclo*Sal)_); ^13^C-NMR (125.8 MHz, CDCl_3_): 29.9 (*C*H_2_CO), 64.9 (POCH_2_), 68.5 (d, ^2^*J*_C-P_ = 6.5 Hz, ArCH_2_OP), 123.5 (d, ^3^*J*_C-P_ = 9.4 Hz, Cl*C*_Ar_C_Ar_OP), 124.1 (CH_Ar(*cyclo*Sal)_), 124.2 (CH_Ar(*cyclo*Sal)_), 124.5 (*C*_Ar_CH_2_OP), 125.6 (CH_Ar(*cyclo*Sal)_), 130.0 (C_Ar_Cl), 130.3 (CH_Ar(*cyclo*Sal)_), 148.5 (d, ^2^*J*_C-P_ = 5.2 Hz, C_Ar_OP); ^31^P-NMR (162.0 MHz, CDCl_3_): −10.3; HRMS (EI)^+^: *m*/*z* calculated for C_11_H_12_ClNO_6_P [M + H]^+^ 308.0085, found 308.0110.
*N-Hydroxy-N-methyl-3-((2-oxido-4H-benzo[d][1,3,2]dioxaphosphinin-2-yl)oxy)propanamide* (**8ba**). The general procedure F was applied to synthesize the compound **8ba** from *cyclo*Sal **16ba** (20 mg, 0.05 mmol) and 2% TFA in DCM. The product **8ba** was obtained without purification as a colorless oil (11 mg, 80%) and as a mixture of two *Z* and *E* conformers in a 30:70 ratio. Rf = 0.30 (EtOAc); ^1^H-NMR (400 MHz, CDCl_3_): 2.76 (30/100H of 2H, bs, CH_2_CO), 2.87–3.06 (70/100H of 2H, m, CH_2_CO), 3.24 (70/100H of 3H, s, NCH_3_), 3.33 (30/100H of 3H, s, NCH_3_), 4.43–4.53 (2H, m, POCH_2_), 5.30–5.44 (2H, m, ArCH_2_OP), 6.84 (1H, bs, OH), 7.05–7.09 (2H, m, CH_Ar(*cyclo*Sal)_), 7.15 (1H, t, ^3^*J* = 7.6 Hz, CH_Ar(*cyclo*Sal)_), 7.32 (1H, t, ^3^*J* = 7.8 Hz, CH_Ar(*cyclo*Sal)_); ^13^C-NMR (125.8 MHz, CDCl_3_): 32.3 (*C*H_2_CO), 33.3 (*C*H_2_CO), 36.3 (NCH_3_), 64.4 (POCH_2_), 65.3 (d, ^2^*J*_C-P_ = 5.3 Hz, POCH_2_), 69.0 (d, ^2^*J*_C-P_ = 6.8 Hz, ArCH_2_OP), 118.9 (d, ^3^*J*_C-P_ = 9.1 Hz, *C*H_Ar_C_Ar_OP), 120.6 (d, ^3^*J*_C-P_ = 10.1 Hz, *C*_Ar_CH_2_OP), 124.8 (CH_Ar(*cyclo*Sal)_), 125.6 (CH_Ar(*cyclo*Sal)_), 130.2 (CH_Ar(*cyclo*Sal)_), 150.0 (d, ^2^*J*_C-P_ = 6.1 Hz, C_Ar_OP), 170.4 (CO); ^31^P-NMR (162.0 MHz, CDCl_3_): −9.7; MS (EI)^+^: *m*/*z* calculated for C_11_H_14_NO_6_PNa [M + Na]^+^ 310.043, found 310.043.
*N-Hydroxy-N-methyl-3-((6-methyl-2-oxido-4H-benzo[d][1,3,2]dioxaphosphinin-2-yl)oxy)propanamide* (**8bb**). The general procedure F was applied to synthesize the compound **8bb** from *cyclo*Sal **16bb** (20 mg, 44 μmol) in 2% TFA in DCM. The product **202b** was obtained without purification as a colorless oil (11 mg, 83%) and as a mixture of two *Z* and *E* conformers in a 30:70 ratio, respectively. Rf = 0.24 (EtOAc); ^1^H-NMR (500 MHz, CDCl_3_): 2.31 (3H, s, CH_3_), 2.77–3.07 (3H, m, CH_2_CO and OH), 3.26 (7/10 of 3H, s, NCH_3_), 3.34 (3/10 of 3H, s, NCH_3_), 4.47 (2H, bs, POCH_2_), 5.27–5.39 (2H, m, ArCH_2_OP), 6.86 (1H, s, CH_Ar(*cyclo*Sal)_), 6.93–6.96 (1H, m, CH_Ar(*cyclo*Sal)_), 7.10 (1H, d, ^3^*J* = 8.3 Hz, CH_Ar(*cyclo*Sal)_); ^13^C-NMR (125.8 MHz, CDCl_3_): 20.9 (CH_3_), 33.3 (*C*H_2_CO), 36.2 (NCH_3_), 65.6 (d, ^2^*J*_C-P_ = 6.4 Hz, POCH_2_), 69.3 (d, ^2^*J*_C-P_ = 6.6 Hz, ArCH_2_OP), 118.6 (d, ^3^*J*_C-P_ = 8.9 Hz, *C*H_Ar_C_Ar_OP), 120.1 (d, ^3^*J*_C-P_ = 9.7 Hz, *C*_Ar_CH_2_OP), 125.8 (CH_Ar(*cyclo*Sal)_), 130.6 (CH_Ar(*cyclo*Sal)_), 134.6 (*C*_Ar_CH_3_), 147.8 (d, ^2^*J*_C-P_ = 7.1 Hz, C_Ar_OP); ^31^P-NMR (162.0 MHz, CDCl_3_): −9.2; MS (EI)^+^: *m*/*z* calculated for C_12_H_16_NO_6_P [M + H]^+^ 302.0788, found 302.077.
*3-((6-Chloro-2-oxido-4H-benzo[d][1,3,2]dioxaphosphinin-2-yl)oxy)-N-hydroxy-N-methylpropanamide* (**8bc**). The general procedure F was applied to synthesize compound **8bc** from *cyclo*Sal **16bc** (87 mg, 0.18 mmol) and 2% TFA in DCM. The product **8bcc** was obtained without purification as a colorless oil (25 mg, 44%) and as a mixture of two *Z* and *E* conformers in a 30:70 ratio. Rf = 0.31 (EtOAc); ^1^H-NMR (500 MHz, CDCl_3_): 2.80 (3/10 of 2H, bs, CH_2_CO), 2.94–3.09 (7/10 of 2H, bs, CH_2_CO), 3.29 (7/10 of 3H, bs, NCH_3_), 3.37 (3/10 of 3H, bs, NCH_3_), 4.51 (2H, bs, POCH_2_), 5.28–5.41 (2H, m, ArCH_2_OP), 7.02 (1H, pseudo-d, ^3^*J* = 8.7 Hz, CH_Ar(*cyclo*Sal)_), 7.09 (1H, pseudo-d, ^3^*J* = 2.3 Hz, CH_Ar(*cyclo*Sal)_), 7.30 (1H, pseudo-d, ^3^*J* = 8.7 Hz, CH_Ar(*cyclo*Sal)_); ^13^C-NMR (125.8 MHz, CDCl_3_): 29.9 (*C*H_2_CO), 32.3 (*C*H_2_CO), 36.2 (NCH_3_), 36.4 (NCH_3_), 64.5 (POCH_2_), 65.6 (d, ^2^*J*_C-P_ = 5.3 Hz, POCH_2_) 68.4 (ArCH_2_OP), 68.6 (d, ^2^*J*_C-P_ = 6.1 Hz, ArCH_2_OP), 120.3 (d, ^3^*J*_C-P_ = 8.8 Hz, *C*H_Ar_C_Ar_OP), 121.9 (*C*_Ar_CH_2_OP), 125.5 (CH_Ar(*cyclo*Sal)_), 130.3 (CH_Ar(*cyclo*Sal)_), 148.5 (C_Ar_OP); ^31^P-NMR (162.0 MHz, CDCl_3_): −10.2; HRMS (EI)^+^: *m*/*z* calculated for C_11_H_13_ClNO_6_PNa [M + Na]^+^ 344.0061, found 344.0030.
*N-Hydroxy-N-methyl-3-((2-oxido-6-(trifluoromethyl)-4H-benzo[d][1,3,2]dioxaphosphinin-2-yl)oxy)propanamide* (**8bf**). The general procedure F was applied to synthesize compound **8bf** from *cyclo*Sal **16bf** (20 mg, 40 μmol) and 2% TFA in DCM. The product **8bf** was obtained without purification as a colorless oil (11 mg, 80%) and as a mixture of two *Z* and *E* conformers in a 40:60 ratio. Rf = 0.57 (EtOAc); ^1^H-NMR (500 MHz, CDCl_3_): 2.77–3.06 (3H, m, CH_2_CO and OH), 3.26 (6/10 of 3H, s, NCH_3_), 3.36 (4/10 of 3H, s, NCH_3_), 4.63 (2H, m, POCH_2_), 5.35–5.49 (2H, m, ArCH_2_OP), 7.16–7.20 (1H, m, CH_Ar(*cyclo*Sal)_), 7.38 (1H, s, CH_Ar(*cyclo*Sal)_), 7.61 (1H, d, ^3^*J* = 8.5 Hz, CH_Ar(*cyclo*Sal)_); ^13^C-NMR (125.8 MHz, CDCl_3_): 31.9 (*C*H_2_CO), 33.3 (*C*H_2_CO), 36.0 (NCH_3_), 36.3 (NCH_3_), 64.5 (POCH_2_), 65.8 (d, ^2^*J*_C-P_ = 6.2 Hz, POCH_2_), 68.7 (d, ^2^*J*_C-P_ = 7.0 Hz, ArCH_2_OP), 119.7 (d, ^3^*J*_C-P_ = 9.2 Hz, *CH*_Ar_C_Ar_OP), 121.2 (d, ^3^*J*_C-P_ = 9.8 Hz, *C*_Ar_CH_2_OP), 123.2 (CH_Ar(*cyclo*Sal)_), 127.5 (CH_Ar(*cyclo*Sal)_), 152.4 (d, ^2^*J*_C-P_ = 7.3 Hz, C_Ar_OP), 170.3 (CO); ^31^P-NMR (162.0 MHz, CDCl_3_): −10.3; ^19^F-NMR (282.4 MHz, CDCl_3_): −63.3; HRMS (EI)^+^: *m*/*z* calculated for C_12_H_13_F_3_NO_6_PNa [M + Na]^+^ 378.0325, found 378.0331.

### 3.2. Bacterial Growth Inhibition

The antimicrobial activity of all *cyclo*Sal prodrugs (**7** and **8**), double prodrugs (**16** and **17**) and Bis(*cyclo*Sal) prodrugs (**18**) against *E. coli* and *M. smegmatis*, was determined using the paper disc diffusion method. A bacterial suspension (200 μL, mid-exponential phase) was spread on agar plates (9 cm diameter) using glass beads. Agar plates contained Luria-Bertani medium for *E. coli* and MS medium for *M. smegmatis* [17]. Paper discs (Durieux no. 268, diameter 6 mm) impregnated with a volume ≤ 8 µL of double prodrugs, *cyclo*Sal prodrugs or Bis(*cyclo*sal) prodrugs were placed on petri dishes. Growth inhibition was examined after a 24 h incubation period at 37 °C. Isoniazid (30 nmoles) and fosmidomycin (10 nmoles) are used as reference compounds for *M. smegmatis* and *E. coli*, respectively.

## 4. Conclusions

The *cyclo*Sal approach has largely been applied to various nucleosides to improve their biological activity in antiviral and cancer therapy. This technique allows an efficient intracellular nucleotide delivery from the pronucleotides via a specific pH-driven mechanism. Even if the *cyclo*Sal strategy might be useful to deliver non-nucleotide phosphorylated molecules, it has not been widely used in the past. We implemented this ProTide strategy to synthesize prodrugs of fosfoxacin and its analogs, inhibitors of the DXR. The synthesized prodrugs were shown to prevent the growth of *M. smegmatis*, with the best candidates being prodrugs with an electron-withdrawing substituent on the *cyclo*Sal moiety and a DMB-protecting group on the hydroxamate. The presence of the DMB, combined with the presence of a *cyclo*Sal prodrug, appears to increase the molecule lipophilicity and, thus, its penetration into the cell. However, two questions still remain: (i) Is the DMB hydrolyzed or supported by an enzyme to liberate the inhibitor? (ii) are the O-DMB-protected inhibitors of the DXR capable of binding in the DXR active site? Further work is currently in progress in our laboratory to answer these questions. Nevertheless, this first report of the use of *cyclo*saligenyl prodrugs on bacteria provided very interesting candidates. They offer insights and new perspectives for the development of antimycobacterial prodrugs.

## Figures and Tables

**Table 1:** *E. coli* and *M. smegmatis* growth inhibition with *cyclo*Sal prodrugs **7** and **8** (800 nmoles) and the reference compounds, fosmidomycin (10 nmoles) and isoniazid (30 nmoles).

*E. coli*		*M. smegmatis*	
Compound	Diameter (mm)	Compound	Diameter (mm)
Fosmidomycin	40–45	Isoniazide	30–35
**7aa**	-	**7aa**	-
**7ba**	-	**7ba**	-
**8aa**	-	**8aa**	-
**8ba**	-	**8ba**	-
**8bb**	-	**8bb**	-
**7ac**	8	**7ac**	8
**7bc**	23	**7bc**	-
**8ac**	-	**8ac**	10
**8bc**	-	**8bc**	8
**8bf**	-	**8bf**	-

**Table 2:** *M. smegmatis* growth inhibition with double prodrugs **16**–**17** (800 nmoles).

Entry	R_1_	R_2_	Prodrugs	Prodrugs Inhibition Zone (mm)	Isoniazide Inhibition Zone (mm)
1	H	H	**16aa**	-	33
2			**16ba**	26	
3			**17aa**	19	31
4			**17ba**	-	
5	CH_3_	H	**16ab**	-	28
6			**16bb**	-	
7			**17ab**	-	
8			**17bb**	-	
9	Cl	H	**16ac**	-	36
10			**16bc**	33	
11			**17ac**	25	35
12			**17bc**	22	
13	Cl	Cl	**16ad**	-	35
14			**16bd**	16	
15			**17ad**	11	
16			**17bd**	12	
17	Br	H	**16ae**	8	42
18			**16be**	31	
19			**17ae**	20	
20			**17be**	21	
21	CF_3_	H	**16af**	-	24
22			**16bf**	25	
23			**17af**	19	
24			**17bf**	17	
25	OCH_3_	H	**16ag**	-	27
26			**16bg**	-	
27			**17ag**	-	
28			**17bg**	-	

**Table 3:** *M. smegmatis* growth inhibition with Bis(*cyclo*sal) prodrugs (800 nmoles) and isoniazide (30 nmoles).

Entry	Bis(*cyclo*sal)	Bis(*cyclo*sal) Inhibition Zone (mm)	Isoniazide Inhibition Zone (mm)
1	**18a**	-	35
2	**18b**	-	35
3	**18c**	31	35
4	**18e**	10	35
5	**18f**	8	24
6	**18g**	-	24

## Data Availability

Data are contained within the article.
